# Fecal microbiota transplantation alters gut phage communities in a clinical trial for obesity

**DOI:** 10.1186/s40168-024-01833-w

**Published:** 2024-07-06

**Authors:** Michele Zuppi, Tommi Vatanen, Brooke C. Wilson, Evgeniia Golovina, Theo Portlock, Wayne S. Cutfield, Mark H. Vickers, Justin M. O’Sullivan

**Affiliations:** 1 Liggins Institute, The University of Auckland, Auckland, New Zealand; 2 Institute of Biotechnology, Helsinki Institute of Life Science, University of Helsinki, Helsinki, Finland; 3 Research Program for Clinical and Molecular Metabolism, Faculty of Medicine, University of Helsinki, Helsinki, Finland; 4 Broad Institute of MIT and Harvard, Cambridge, MA USA; 5 A Better Start - National Science Challenge, University of Auckland, Auckland, New Zealand; 6 The Maurice Wilkins Centre, The University of Auckland, Auckland, New Zealand; 7 MRC Lifecourse Epidemiology Unit, University of Southampton, Southampton, UK; 8 Australian Parkinson’s Mission, Garvan Institute of Medical Research, Sydney, NSW Australia; 9 A*STAR Singapore Institute for Clinical Sciences, Singapore, Singapore; 10 Garvan Institute of Medical Research, Sydney, NSW Australia

**Keywords:** Fecal microbiota transplantation (FMT), Phageome composition, Donor microbiome diversity, Microbial engraftment, Microbial population dynamics

## Abstract

**Background:**

Fecal microbiota transplantation (FMT) is a therapeutic intervention used to treat diseases associated with the gut microbiome. In the human gut microbiome, phages have been implicated in influencing human health, with successful engraftment of donor phages correlated with FMT treatment efficacy. The impact that gastrointestinal phages exert on human health has primarily been connected to their ability to modulate the bacterial communities in the gut. Nonetheless, how FMT affects recipients’ phage populations, and in turn, how this influences the gut environment, is not yet fully understood. In this study, we investigated the effects of FMT on the phageome composition of participants within the Gut Bugs Trial (GBT), a double-blind, randomized, placebo-controlled trial that investigated the efficacy of FMT in treating obesity and comorbidities in adolescents. Stool samples collected from donors at the time of treatment and recipients at four time points (i.e., baseline and 6 weeks, 12 weeks, and 26 weeks post-intervention), underwent shotgun metagenomic sequencing. Phage sequences were identified and characterized in silico to examine evidence of phage engraftment and to assess the extent of FMT-induced alterations in the recipients’ phageome composition.

**Results:**

Donor phages engrafted stably in recipients following FMT, composing a significant proportion of their phageome for the entire course of the study (33.8 ± 1.2% in females and 33.9 ± 3.7% in males). Phage engraftment varied between donors and donor engraftment efficacy was positively correlated with their phageome alpha diversity. FMT caused a shift in recipients’ phageome toward the donors’ composition and increased phageome alpha diversity and variability over time.

**Conclusions:**

FMT significantly altered recipients' phage and, overall, microbial populations. The increase in microbial diversity and variability is consistent with a shift in microbial population dynamics. This proposes that phages play a critical role in modulating the gut environment and suggests novel approaches to understanding the efficacy of FMT in altering the recipient’s microbiome.

**Trial registration:**

The Gut Bugs Trial was registered with the Australian New Zealand Clinical Trials Registry (ACTR N12615001351505). Trial protocol: the trial protocol is available at https://bmjopen.bmj.com/content/9/4/e026174.

Video Abstract

**Supplementary Information:**

The online version contains supplementary material available at 10.1186/s40168-024-01833-w.

## Introduction

Fecal microbiota transplantation (FMT) is a therapeutic approach currently being investigated for the treatment of numerous diseases associated with the gut microbiome, including recurrent *Clostridium difficile* infections (rCDI) [1], inflammatory bowel disease (IBD) [2], and obesity [3]. The rationale behind FMT is to restore a healthy gut environment through the engraftment of gut microbes from a healthy donor. Although bacteria are usually the focus of FMT studies, the contributing role of gastrointestinal phages in the efficacy of FMT has been gaining attention. The transfer and successful engraftment of donor phages has been observed in FMT clinical trials for the treatment of multiple diseases (i.e., CDI [4], metabolic syndrome [5], and autism spectrum disorder [6]). Furthermore, a shift in the virome composition of recipients toward that of the donor(s) was positively correlated with FMT efficacy in CDI [4] and metabolic syndrome [5]. Of note, fecal filtrate (FFT) or fecal virome transplantation (FVT), namely the transplantation of fecal material from which bacterial cells have been removed [7], proved effective in treating rCDI in humans [8] in a manner comparable to the transplantation of the whole gut microbiome [9]. FVT has also been used to effectively treat necrotizing enterocolitis in piglets [10] and decrease the symptoms of type 2 diabetes in mice [11]. While other components, such as bacterial-free DNA, vesicles, and metabolites, might be contributing to the efficacy of FVT, phages are likely to play a major role [7].

The impact that phages have on the environments they inhabit is often correlated with their ability to modulate bacterial communities [12–15]. This modulation is strictly dependent on the life cycle followed by the phages (e.g., lytic or lysogenic), which can lead to different modalities of interactions with the bacteria they infect [16]. In the gastrointestinal tract, phage predatory activity has been suggested to affect human health, with both detrimental and beneficial effects [17, 18]. Phage-mediated lysis of bacterial cells has been suggested to contribute to gastrointestinal inflammation in IBD patients [17]. Conversely, the induction of temperate *Klebsiella*-infecting phages has been associated with the depletion of multidrug-resistant *Klebsiella* spp in humans [18]. Despite the potential for gut phages to affect human health, many aspects of their importance in the gut microbiome and FMT are yet to be fully characterized.

In the present study, we utilized previously published metagenomic sequencing data [19] to characterize the gut phageome composition of participants in an FMT trial for adolescent obesity (the Gut Bugs Trial, GBT) [20]. Specifically, we assessed the efficacy of FMT in promoting donor phage engraftment and examined its effects on the resident phage population. The findings were also analyzed in the context of the data previously reported on bacterial populations in the GBT participants [19] to examine the impact of FMT on interactions between phages and bacteria.

Methods.

### The Gut Bugs Trial

**Fig. 1:**
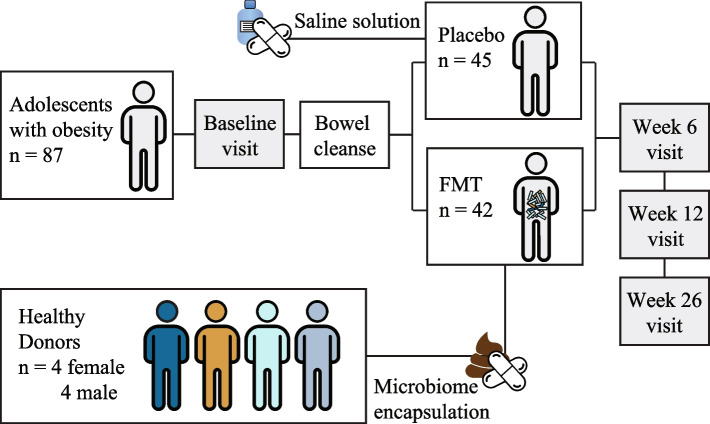
Schematic representation of the Gut Bugs Trial. Whole stools were collected from 8 healthy lean donors (4 females and 4 males). Following the first donation, a single male donor (DM05) was replaced by another (DM12). Donor stool and saline solution were administered in a double-blind manner to FMT (*n* = 42) and placebo (*n* = 45) recipients, respectively. Recipient stool samples were collected at baseline and at 6, 12, and 26 weeks post-intervention to investigate the effects of FMT on recipient microbial populations

The GBT was a double-blind, randomized, placebo-controlled clinical trial designed to test the efficacy of FMT in treating obesity-related conditions in adolescents [20, 21] (Fig. 1). Ethical approvals were obtained from the Northern A Health and Disability Ethics Committee of New Zealand (16/NTA/172).

Stool samples were collected from healthy lean donors (BMI 22.7 ± 1.9 kg/m^2^ (SD), total body fat 18.4 ± 3.2% (SD)) aged between 19 and 27 years old [20]. The participants were 87 adolescents with obesity (age 14–19, BMI 30.2 ± 5.3 kg/m^2^); 51 females and 36 males [21].

The fecal microbiota transplantation was performed as described previously [19]. Donor gut microbiota was encapsulated in acid-resistant DR Caps™ (Capsugel, USA) capsules (0.25 g per capsule) after suspension in a cryoprotective saline solution (0.5 ml 0.9% NaCl, 15% glycerol). Placebo capsules were prepared with only the cryoprotective saline solution. The participants were given either FMT or placebo capsules following a bowel cleanse and an overnight fast in a double-blind fashion. The capsules were administered in a sex-specific manner (males to males and females to females), and FMT recipients ingested 28 capsules in total (7 from each donor) during the course of 2 days (16 the first day and 12 the second day).

### Metagenomic sequencing

Shotgun metagenomic sequencing was conducted as described previously [19] on recipient stool samples collected at 4 time points (baseline (before bowel cleansing), 6, 12, and 26 weeks post-treatment), and donor samples collected at each donation round. Sequencing was performed using Illumina’s 150 bp paired-end read technology, generating 23 ± 3 million read pairs/sample (mean ± SD). Raw sequencing reads were pre-processed with KneadData (*v*0.7.4) [22] to trim and remove low-quality reads and those mapping to the human genome. Post-processed reads and accompanying metadata are available on NCBI’s SRA database (BioProject PRJNA637785).

### Identification of viral contigs and construction of viral operational taxonomic units

Contigs were assembled from post-processed reads using the de novo assembler MegaHIT (*v*1.1.4) [23] under default parameters. Putative viral contigs were identified using a modified version of a Standard Operating Procedure (SOP) proposed previously [24]. Briefly, the assembled contigs were analyzed using VirSorter2 (*v*2.2.3), [23, 25] and subsequently analyzed using CheckV (*v*0.7.0) [26, 27]. Contigs were considered putative viral contigs if they contained at least one viral gene predicted by CheckV or matched at least one of the following requirements: (1) no host genes, (2) VirSorter2 score ≥ 0.95, or (3) three or more hallmark genes. Hallmark genes, identified by VirSorter2, are genes that are commonly associated with viral genomes [23]. Putative viral contigs were considered false positives if the predicted number of host genes was > 4 or the ratio of host-to-viral genes (HVR) was > 0.386. The specific host genes and HVR cut-off values were chosen accordingly to the methods described in Additional file 1. Putative viral contigs were thereafter referred to as uncultivated virus genomes (UViGs) [28].

UViGs were clustered into vOTUs (viral operational taxonomic unit) using a custom script according to MIUViG (Minimum Information about an Uncultivated Virus Genome) standards (i.e., average nucleotide identity (ANI) ≥ 95% over alignment fraction (AF) ≥ 85% of the smaller contig [28]). The ANI and AF were calculated with FastANI (*v*1.33) [29], using the protocol adapted from Kauffman et al. [30] to meet the MIUViG standards. The longest UViG in each vOTU was used as the representative of that vOTU. All the UViGs clustering with the vOTU-representative UViG were considered as part of the vOTU. Only the vOTUs represented by a UViG with completeness ≥ 90% (i.e., representing a high-quality draft genome [28]) were used in downstream analyses to maximize the prediction accuracy of the software employed. The metadata requested by MIUViG standards for the vOTU-representative UViGs is reported (Additional Table S1) and is publicly available on Figshare (https://doi.org/10.17608/k6.auckland.24654891).

### Identification of clonal UViGs and engrafted phages

Two UViGs belonging to the same vOTU were considered clones based on sequence similarities (ANI ≥ 99.4% over AF ≥ 85%). The specific ANI and AF cut-off values were chosen according to the methods described in Additional file 1. A donor phage was considered engrafted whenever a clonal UViG could be identified in a recipient following (and not prior) the transplantation (i.e., from 6 weeks forward).

### UViGs characterization

For each UViG, taxonomy, host specificity, and lifestyle were analyzed. UViGs taxonomy was determined using vContact2 (*v*0.9.19) [31, 32]. Additionally, Hidden Markov Models (HMM) profile similarities with the viral orthologous gene database ViPhOGs (29/06/2021) [33] were employed to further determine the taxonomy of the UViGs, as described previously [34]. Host specificity was predicted with VirHostMatcher-Net (*v*1.0) [35] using the in-built Human Microbiome Project list of bacterial genomes, retaining only the first hit [36]. UViGs lifestyle (i.e., virulent or temperate) was predicted using Bacphlip *(v*1.0) with default parameters [37, 38].

### UViGs relative abundance and individual phageome composition

Open reading frames (ORFs) were initially predicted using Prodigal (*v*2.6.3) [39], and clustered using CD-HIT (*v*4.6.6) [40] at 95% nucleotide identity over 85% of the alignment fraction (*cd-hit -c 0.95 -n 5 -aS 0.85 -M 6000*), to produce a non-redundant gene catalog. Lastly, gene cluster abundance was estimated by aligning the quality-controlled sequencing reads against the cluster centroids, using Burrows-Wheeler Aligner (*v*0.7.17) [41] with the BWA-MEM algorithm (*bwa mem*) using a custom script. The number of reads clustering against each gene cluster centroid was calculated and normalized by gene length, obtaining the Reads Per Kilobase (RPK) value. Gene RPK values were normalized by the total number of RPK sequenced from each individual (to account for different sequencing depths) and multiplied by 1,000,000 to obtain the measure of copies per million (CPM). UViGs relative abundances were estimated by calculating the median CPM value across all genes present on the UViG. vOTU relative abundances were calculated by summing the abundances of all clustering UViGs specific to that vOTU for each sample. The proportion of total reads aligned against the non-redundant catalog of viral genes was estimated using the flagstat command from SAMtools (*v*1.10) [42].

Phageome composition was based on vOTUs relative abundance profiles. The Shannon Diversity Index was used to estimate the alpha diversity of vOTU profiles using the ‘diversity()’ function from the vegan package (*v*2.6.2) [43] in R (*v*4.0.3). Bray–Curtis dissimilarities between vOTU profiles, where zero indicates identical profiles and one completely different profile, were used as a beta diversity metric and were calculated using vegan’s ‘vegdist()’ function.

### Statistical analyses

Statistical analyses were performed in R (*v*4.0.3). The *p* values were adjusted using the false discovery rate (FDR) correction whenever multiple comparisons were performed.

The methods are described in more detail in Additional file 1.

## Results

A total of 25,805 vOTUS were obtained from the full dataset (381 samples), and 6.8% of these (*n* = 1761) were represented by UViGs with predicted completeness ≥ 90% and were used for the analysis. The Illumina sequencing produced on average 22.5 ± 2.9 million reads per sample, 17.96 ± 3.32% of which mapped against the UViGs (Additional file 2, Additional Figure S4).

### 282 donor phages engrafted in the recipients following FMT

**Fig. 2:**
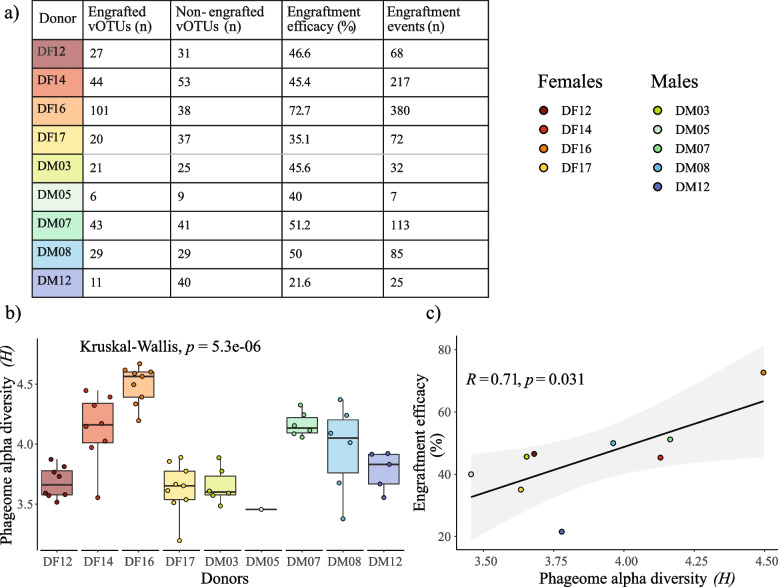
Donor phage engraftment was associated with phageome alpha diversity. **a** Donors differed in the number of engrafted vOTUs donated and in engraftment efficacy. **b** Donors differed in phageome alpha diversity (Shannon diversity). Each dot represents a different donation. **c** Positive correlation between the donor’s phageome alpha diversity and phage engraftment efficacy (assessed by Pearson correlation)

To evaluate the efficacy of FMT in promoting the engraftment of donor phages, the sequence similarities between donor and recipient UViGs were assessed. Two hundred eighty-two unique vOTUs were predicted to have been engrafted across a total of 999 engraftment events (Fig. 2a).

Engraftment efficacy, defined as the proportion of donor vOTUs that engrafted relative to the total number of vOTUs identified in the donor, differed between donors. (Pearson’s chi-squared (*χ*^2^) test, *p* = 1.5e^−8^). The majority of the engrafted vOTUs were donated by female donors (*n* = 192, 68.1%), specifically DF14 and DF16, which donated 44 and 101 unique vOTUs, respectively (Fig. 2a). With respect to the male donors, the majority of the vOTUs were donated by donor DM07, with 43 unique vOTUs out of a total of 110 (40.9%). It is important to note that for 20 engrafted vOTUs, it was not possible to determine which donor they originated from, as they were identified in multiple donors. As a result, these vOTUs were considered as if donated by multiple donors. DF16 had significantly higher engraftment efficacy compared to other donors (post hoc analysis, *p* = 1.8e^−8^). In contrast, DM12 had the lowest engraftment efficacy of the donors (post hoc analysis, *p* = 4.2e^−4^). Despite inter-donor differences, the majority of the total number of unique vOTUs identified across all the donors were engrafted (63%, 282 out of 451).

Successful engraftment of phages was related to the donor’s phageome alpha diversity (Fig. 2b), with the most diverse donors (i.e., DF14, DF16, and DM07) contributing the most engrafted vOTUs (Pearson correlation, *p* = 0.03) (Fig. 2c).

Engrafted vOTUs did not differ significantly from the non-engrafted vOTUs with respect to taxonomy, host specificity, lifestyle, and relative abundance (Additional file 2). However, a bacterial host could be predicted more frequently for the engrafted vOTUs than non-engrafted vOTUs (post hoc analysis on *χ*^2^ test, *p* = 1.3e^−8^). The higher frequency of host predictions for engrafted phage could indicate that these vOTUs are commonly identified in the human gut microbiome and have, therefore, a higher representation in databases. These observations suggest that the engraftment efficacy of a phage was not dependent on specific phage characteristics but rather influenced by donors’ gut microbial diversity.

### The phageome composition of the recipients converged toward specific donor profiles

**Fig. 3:**
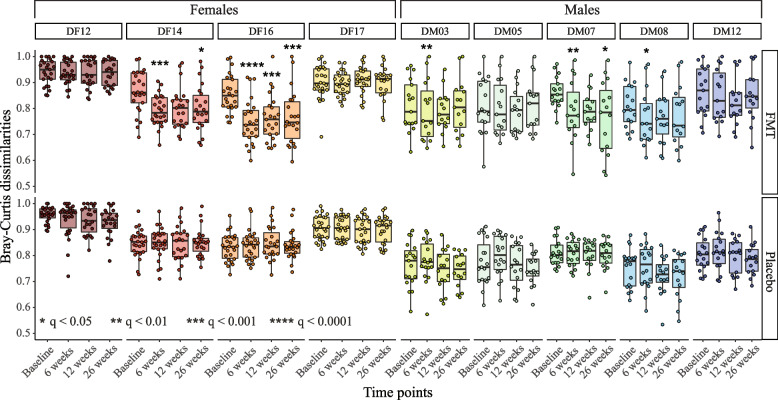
FMT recipients’ phageome composition shifted towards donor profiles. Bray–Curtis dissimilarities between donor and recipient vOTU profiles, stratified by sex-matched donors and time point. Significant shifts towards individual donor profiles were assessed using linear mixed-effects models (LMM) including a treatment by time point interaction (fixed effect) and subject ID (random effect). The *p* values were collectively adjusted using the FDR correction, and only statistically significant differences (i.e.,* q* ≤ 0.05) were reported

To identify shifts in phageome composition following FMT, we next compared recipients’ phageome to individual donor profiles by measuring Bray–Curtis dissimilarities (Fig. 3). We identified convergence in female FMT recipients towards donor DF16’s phageome, and to a lesser extent DF14. Male FMT recipients exhibited compositional shifts towards DM07 and, to a lesser extent, DM03 and DM08 phageomes. These compositional shifts were specific to FMT recipients and were not detected in the placebo group (Fig. 3). As expected, the donors toward which the recipients’ phageome shifted the most were the same donors that contributed the most engrafted vOTUs (Figs. 2a and 3).

### Donor-engrafted vOTUs are retained within the recipient’s gut long-term

**Fig. 4:**
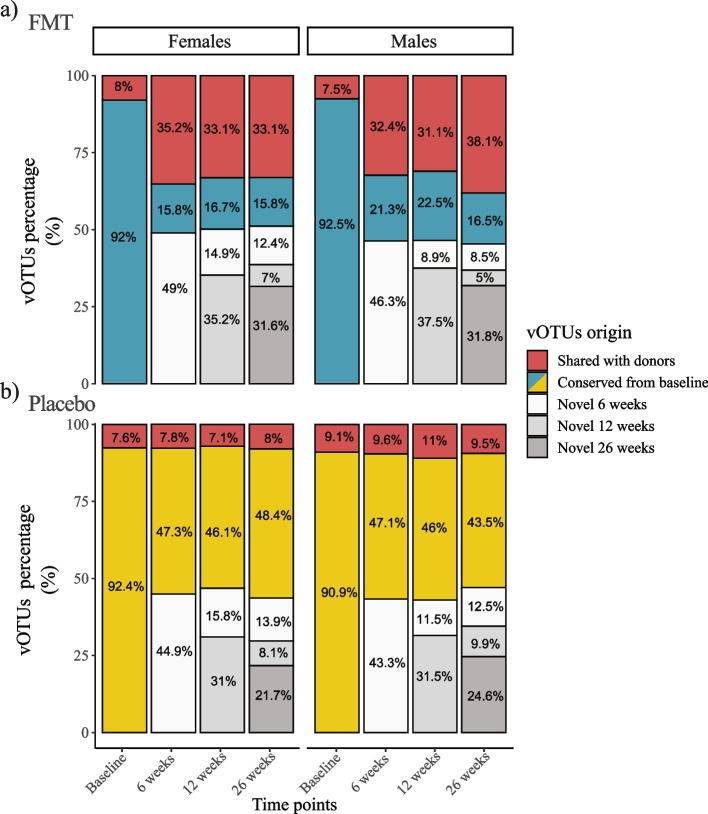
FMT and placebo recipients had distinct phageome compositions. The phageome compositions of **a** FMT and **b** placebo recipients were categorized according to the origin of the vOTUs: (1) vOTUs shared with the donors (i.e., shared with donors); (2) vOTUs that were initially identified at baseline within the FMT or placebo recipients (i.e., conserved from baseline), (3) vOTUs that were identified at time points subsequent to the transplantation in FMT or placebo recipients (i.e., novel)

Following engraftment, the number of vOTUs shared between the FMT recipients and donors was significantly increased from baseline (*χ*^2^ test, *p* < 2.2e^−16^) at all post-FMT time points in both females and males (Fig. 4a).

By comparison, the proportion of donor-matching vOTUs within placebo recipient microbiomes remained unchanged throughout the study period and was significantly lower than that observed in the FMT group post-treatment (*χ*^2^ test, *p* < 2.2e^−16^) (Fig. 4b). Collectively, these results indicate that FMT resulted in successful engraftment of donor phages within its recipients.

Acquisition of novel vOTUs that did not match donor or recipient vOTUs at baseline remained consistent throughout the course of the study in both treatment groups (FMT recipients: 50 ± 1 in females and 46 ± 0.6 in males; placebo recipients: 45.1 ± 1.6 in females and 44.4 ± 2.2 in males) (Fig. 4). The proportion of novel vOTUs was higher in FMT recipients at all time points (*χ*^2^ test, FDR correction, 6 weeks *q* = 0.04, 12 weeks *q* = 0.04, 26 weeks *q* = 0.04). The phageome fraction composed of novel vOTUs exhibited a significantly higher variability compared to the fraction of vOTUs conserved from baseline and shared with the donors (Additional file 2). This pattern was consistent in both FMT and placebo recipients. The variability was defined as the shift in phageome composition (Bray–Curtis dissimilarities) from 6 weeks at subsequent time points within individuals.

### FMT increased the variability and diversity of recipient gut phageomes

**Fig. 5:**
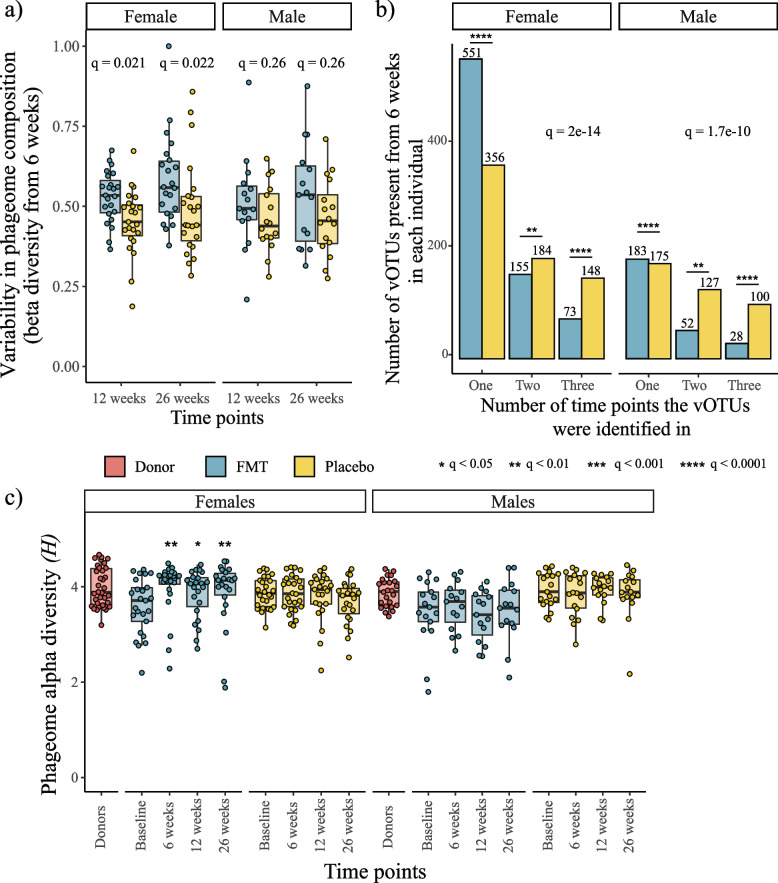
Increase in phageome variability and alpha diversity in FMT recipients post-transplantation. **a** Bray–Curtis dissimilarities between participants’ week 6 phageome and phageomes from subsequent follow-up time points. Comparisons between treatment groups were performed using Wilcoxon rank sum tests with FDR correction. **b** vOTUs identified in FMT recipients were less stable than the vOTUs identified in placebo individuals. Inter-group differences were assessed using Pearson’s chi-squared tests. To determine which variables contributed to the overall inter-group differences, a post hoc analysis was performed, and the *p* values were adjusted using the FDR correction. **c** Female FMT recipients exhibited an increase in phageome alpha diversity at 6 weeks following the transplantation. The differences in variation in alpha diversity post-FMT between FMT and placebo recipients were assessed using linear mixed-effects models. The *p* values were collectively adjusted using the FDR correction, and only statistically significant differences (i.e., *q* ≤ 0.05) were reported

The higher proportion of novel vOTUs acquired by FMT recipients at each time point suggested that the transplantation led to an increase in variability in the phageome composition of its recipients. Alongside the acute shift from the baseline phageome composition, a continuous shift could also be observed in FMT recipients post-transplantation. Specifically, at 12 weeks following the transplantation, the phage populations exhibited significantly higher variability in composition in female FMT recipients compared to placebo (Fig. 5a).

This higher variability was defined as the phageome beta diversity (Bray–Curtis dissimilarities on vOTU composition) at subsequent time points (i.e., 12 weeks and 26 weeks) from the first time point post-transplantation in which stool samples were collected (i.e., 6 weeks).

Further illustrating the increase in variability in phageome composition, the vOTUs identified in FMT recipients were overall less stable over time compared to placebo (Fig. 5b). Specifically, FMT recipients exhibited a higher proportion of vOTUs that could only be identified at one time point, compared to placebo. Conversely, placebo individuals had a higher proportion of vOTUs that could be identified at multiple time points. This difference was significant in both female (*χ*^2^ test, *p* = 3.8e^−9^) and male (*χ*^2^ test, *p* = 4.2e^−5^) individuals (Fig. 5b).

Female FMT recipients exhibited an increase in phageome alpha diversity post-transplantation that was significantly higher than the one exhibited by placebo recipients (LMM, FDR correction, 6 weeks *q* = 0.005, 12 weeks *q* = 0.029, 26 weeks *q* = 0.005) (Fig. 5c). By contrast, phageome alpha diversity in the males did not change over the study period.

### FMT recipients exhibited an increase in the total abundance of temperate phages

**Fig. 6:**
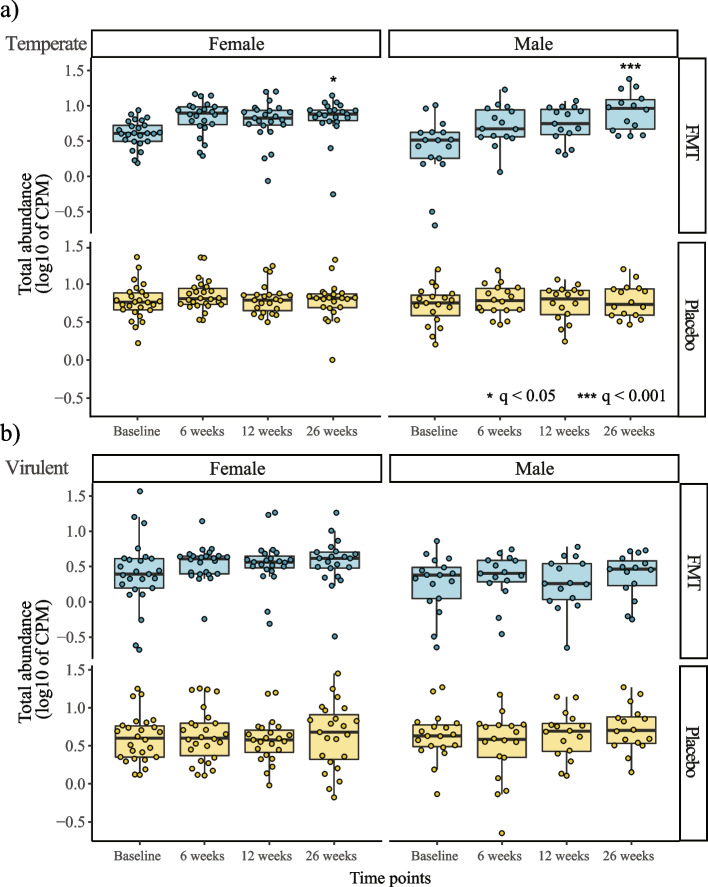
The total abundance of temperate phages increased post-FMT. Total abundance of temperate and virulent phages in **a** FMT and **b** placebo recipient microbiomes. Treatment differences in variation in the total abundance of the temperate and virulent phages post-FMT between FMT and placebo recipients were assessed using linear mixed-effects models. The *p* values were collectively adjusted using the FDR correction within sexes. Only statistically significant differences (*q < 0.05, ***q < 0.001) were reported

FMT recipients exhibited an increase in the total abundance of temperate phages post-transplantation, which was significantly higher than placebo recipients at 26 weeks (LMM, FDR correction, females *q* = 0.048, males *q* = 3.66e^−4^) (Fig. 6a).

A similar trend could be observed at earlier time points (i.e., 6 and 12 weeks) in both females and males, although did not reach statistical significance (*q* = 0.107).

Conversely, the total abundance of virulent phages did not change post-treatment and was not statistically different between treatment groups (LMM, FDR correction, *q* > 0.05) (Fig. 6b).

## Discussion

Transplantation of fecal microbiota from multiple lean donors altered the recipients’ phageome composition. Donor-derived phages comprised a considerable proportion of the recipient phageome following transplantation. Despite FMT recipients receiving an equal dose of capsules from the same set of four donors, higher levels of phage engraftment were seen for donors with higher phage diversity. FMT was also shown to promote phageome variability and diversity.

The Gut Bugs Trial was a multi-donor fecal transplantation clinical trial stratified by sex, in which the stool samples of multiple donors (i.e., four females and five males) were pooled together to create the capsules given to the recipients. Analysis of the bacteriome of the participants in the GBT revealed that FMT donors differed in engraftment efficacy and that the microbiome composition of the recipients shifted toward one of specific donors [19]. A similar observation could also be made in the phageome, as female donors DF14 and DF16 had the highest impact on the phageome of female recipients. The phageome composition of male recipients shifted primarily toward the composition of DM07, although a significant shift toward DM03 and DM08 was observed at 6 weeks. The high engraftment efficacy of DF16 could also explain the sex-specific differences observed in the efficacy of FMT in altering the recipient microbial populations [19]. Consistent with what was observed in the bacterial component [19], the engraftment efficacy of a donor was positively correlated with its microbial alpha diversity. A high diversity of microbes invading a new environment likely increases the probability of these finding niches to colonize. We observed 20 vOTUs that were identified in multiple donors and engrafted in FMT recipients. Speculatively, these vOTUs might be preferentially selected and engrafted through gene and single nucleotide polymorphism level adaptations. Future studies could fruitfully investigate the adaptation and evolution of such shared vOTUs.

The importance of the donor’s microbiome composition in microbial engraftment in FMT is currently under debate [44–47]. Two meta-analyses did not identify any significant correlation between the donor’s microbiome composition and microbial engraftment efficacy in FMT, reporting that this was prevalently connected to the recipient’s microbiome composition and microbial compositional similarities between donors and recipients at baseline [44, 45]. In contrast, other studies have reported a positive correlation between the donor’s microbiome alpha diversity and engraftment efficacy [46, 47]. The importance of the donor’s microbiome in determining microbial engraftment appears to be connected to the state of the recipient’s microbiome prior to transplantation—a high alpha diversity in the microbiome of FMT recipients has been negatively correlated with microbial engraftment [46]. Furthermore, antibiotic treatment of recipients prior to the transplantation led to a higher proportion of donor phages engrafting [44]. Consistent with this, the participants in the GBT underwent bowel cleansing prior to the transplantation [21], which may have been a contributing factor to the donor-dependent engraftment efficacy observed in this study by reducing recipients' microbial load [48].

Despite the phageome of both FMT and placebo recipients being subjected to variation in time, the phageome of female FMT recipients exhibited a higher variability compared to the placebo group. Similar trends were also observed in male recipients. This variability was further exemplified by the lower stability of the vOTUs identified in FMT recipients, as the recipient’s vOTUs could be less frequently identified at multiple time points compared to the placebo’s vOTUs. Consistent with this, the phageome of FMT recipients was composed of a higher proportion of novel vOTUs compared to the placebo group. Remarkably, a similar increase in variability could also be observed in the bacterial component of FMT recipients analyzed at a species level [19], in which a higher proportion of novel species could be identified at all time points. As suggested by Wilson et al., this increase in variability could be either the result of (1) a higher fluctuation in strain abundance above and below the detection threshold, (2) a significant alteration in the gut microbiome that facilitates the acquisition of environmental strains, or (3) a combination of both [19].

In addition to this, an increase in alpha diversity could be observed in both the phage and bacterial populations of female FMT recipients. The increase in variability and diversity exhibited by both phage and bacterial populations in FMT recipients is consistent with a change in the prevalent population dynamic between them. Specifically, it suggests a shift from a prevalent Piggyback-the-Winner (PtW) or Arms-race dynamic (ARD) (i.e., low variability and diversity of microbial populations) to a Fluctuating-selection (FSD) or Kill-the-Winner dynamic (KtW) (i.e., high variability and diversity of microbial populations) [49]. PtW dynamic is considered the dominant population dynamic in the lumen of the gastrointestinal tract of humans [50]. It is the result of the mutualistic interaction between phages and bacteria, which occurs during the phage’s lysogenic cycle. The other dynamics reported above are driven by the predator–prey interaction between phages and bacteria, which stems from the phage reproductive lytic cycle.

FMT-mediated alteration of microbial population dynamics has previously been observed by Liu et al. [18], who found that higher abundances of *Klebsiella*-infecting phages following FMT was associated with a decrease in the abundance of *Klebsiella* spp. The authors suggested that this shift was due to FMT-promoted changes in the population dynamics between *Klebsiella* spp and the infecting phages, which led toward a dynamic characterized by an active phages reproductive cycle, i.e., Kill-the-Winner dynamic [18, 51].

Interestingly, FMT recipients exhibited an increase in the abundance of temperate phages following the transplantation, which could not be observed in virulent phages or in the placebo group. Consistent with what was proposed previously by Liu et al. [18], the increase in the abundance of temperate phages could have been the result of their switch from the dormant lysogenic cycle to the reproductive lytic cycle. Nonetheless, the analyses were performed using metagenomic samples prepared from the whole stool, and free-living phages could not be distinguished from dormant integrated ones. Moreover, only a small percentage of phages could be associated with a bacterial host (Additional file 2, Additional Figure S3), and the direct effects of phage predation on their bacterial host could not be assessed in this analysis. Further research is therefore needed to ascertain the role of FMT in influencing the life cycle followed by temperate phages.

It is important to note that out of the 25,805 total vOTUs identified in the Gut Bugs Trial, 93.2% were represented by UViGs with either low or medium quality, and could, therefore, not be used in the analysis. This represents an ongoing challenge with gut phageome analysis, as the majority of the gut phageome, usually referred to as “viral dark matter”, remains uncharacterized [52]. Using a higher sequencing depth (e.g., 100 million reads per sample) or complementing bulk metagenomic analysis with viral-like particle enrichment could improve the characterization of less abundant phages.

The potential influence of FMT in altering microbial interactions provides novel insights into understanding its effects on recipients’ microbial populations and the gastrointestinal tract at large. Furthermore, it highlights the pivotal role of phages in the gut ecosystem and the importance of including their analysis in future studies on FMT and the gut microbiome. 

## Conclusions

In the Gut Bugs Trial, FMT promoted stable changes in the phageome composition of its recipients, which were conserved throughout the study. Upon receiving equal amounts of stool from different donors, the phageome of FMT recipients shifted toward the composition of the donors with the highest phageome alpha diversity. Despite the importance of donor microbial composition in promoting microbial engraftment currently being under debate, these results suggest that a higher microbial diversity was coupled with a more successful engraftment. The selection of donors with high microbial diversity could, therefore, be an important factor in improving the effectiveness of FMT in altering recipient microbial populations. Finally, FMT promoted an increase in the variability and diversity of both phage and bacterial populations, consistent with an alteration in microbial population dynamics.

### Supplementary Information

Additional file 1. Additional methods. In this file are reported the detailed methods used in this study: 1) Identification of putative viral contigs and production of Uncultivated Virus Genomes (UViGs); 2) Removal of false positive putative viral contigs; 3) Identification of clonal UViGs; 4) UViGs characterization; 5) Statistical analyses.

Additional file 2. Additional results. In this file are reported additional results that were mentioned in the main text: 1) Output of the phage identification workflow; 2) Analysis of the engrafted vOTUs; 3) The gut phageome comprised of a stable and a fluctuating component.

Additional file 3.** Additional Table S1**. Table reporting the metadata requested by MIUViG standards for the description of UViGs. In the first page are reported the MIUViG guidelines for the required metadata. In the second page are reported the metadata of the UViGs obtained in this study. In the following pages are reported the metadata of each vOTU-representative UViG analized in this study: 1) UViG name; 2) VirSorter2 score; 3) Predicted genome type; 4) whether it was identified as provirus or not by CheckV; 4) CheckV quality; 5) Completeness (%); 6) MIUViG quality; 7) Approach used by CheckV to determine completeness; 8) Taxonomy (Family); 9) Host specificity (Phylum); 10) Host specificity (Species).

## Data Availability

Post-processed reads and accompanying metadata are available on NCBI’s SRA database (BioProject PRJNA637785).

## Abbreviations

AF: Alignment fraction
ANI: Average nucleotide identity
ARD: Arms-race dynamic
BMI: Body mass index
CDI: Clostridium difficile infection
CPM: Copies per million
DF: Female donor
DM: Male donor
FDR: False discovery rate
FFT: Fecal filtrate transplantation
FMT: Fecal microbiota transplantation
FSD: Fluctuating selection dynamic
FVT: Fecal virome transplantation
GBT: Gut Bugs Trial
HMM: Hidden Markov models
HVR: Host-to-viral genes ratio
IBD: Inflammatory bowel diseases
KtW: Kill-the-winner
LMM: Linear mixed-effects models
MIUViG: Minimum information about an uncultivated virus genome
ORF: Open reading frame
PtW: Piggyback-the-winner
rCDI: Recurrent *Clostridium difficile* infection
RPK: Reads per kilobase
SOP: Standard operating procedure
UViG: Uncultivated virus genome
vOTU: Viral operational taxonomic unit
*χ*^2^: Pearson’s chi-squared
