# Supplementary material for: Fecal microbiota transplantation alters gut phage communities in a clinical trial for obesity
Source: Microbiome. 2024 Jul 6;12:122. doi: 10.1186/s40168-024-01833-w (PMC11227244; doi:10.1186/s40168-024-01833-w)
Supplement: Supplementary file 2 — Additional file 1. Additional methods. In this file are reported the detailed methods used in this study: 1) Identification of putative viral contigs and production of Uncultivated Virus Genomes (UViGs); 2) Removal of false positive putative viral contigs; 3) Identification of clonal UViGs; 4) UViGs characterization; 5) Statistical analyses. [file 40168_2024_1833_MOESM1_ESM.docx]

Additional File 1

# Identification of putative viral contigs and production of Uncultivated Virus Genomes (UViGs)

Putative viral contigs were identified using a modified version of Sullivan's Lab’s SOP [[2]](https://paperpile.com/c/1tcawA/kPRAg). Putative viral contigs were initially identified using VirSorter2 (*v*2.2.3), [[3](https://paperpile.com/c/1tcawA/Af6ur), [4]](https://paperpile.com/c/1tcawA/OxNUL) (*virsorter run --keep-original-seq --include-groups dsDNAphage,ssDNA --min-length 4000 --min-score 0.5 -j 28 all*). To maximize the recovery of *Microviridae* phages with small genomes [[5, 6]](https://paperpile.com/c/1tcawA/ysKpg+BzITo), the minimum genome length allowed for viral identification proposed in the SOP [[2]](https://paperpile.com/c/1tcawA/kPRAg) was reduced from 5000 bp to 4000 bp (*--min length 4000*). The putative viral contigs identified by Virsorter2 were then analyzed using CheckV (*v*0.7.0) [[1, 7]](https://paperpile.com/c/1tcawA/0FJA+Zpnzo) (*checkv end_to_end ~/db/checkv-db-v1.0*). The contigs were considered putative viral contigs if they presented at least one viral gene predicted by CheckV or if they did not harbor any predicted viral genes but matched at least one of the following requirements: 1) no host genes, 2) Virsorter2 score ≥ 0.95, or 3) three or more hallmark genes. Hallmark genes, identified by VirSorter2, are commonly associated with viral genomes [[4]](https://paperpile.com/c/1tcawA/OxNUL).

# Removal of false positive putative viral contigs

Manual curation of the putative viral contigs identified through the SOP was performed to remove false positives. A putative viral contig was considered to be a false positive based on the predicted number of host genes and their ratio with viral genes (HVR) (*i.e.*, Host-to-Viral genes Ratio or HVR > 0.386 & host genes > 5). 3,372 (14.3%) of the total putative viral contigs were removed from the analysis. The use of HVR and the number of host genes to call false positives was previously suggested by Nayfach et al. [[1]](https://paperpile.com/c/1tcawA/0FJA) (HVR > 2 & host genes > 5). The threshold used in this study was selected based on the average number of host genes and HVR found in complete putative viral contigs. Firstly, putative viral contigs predicted to be complete were selected. These contigs were dereplicated into vOTUs (viral Operational Taxonomic Units; 95% Average Nucleotide Identity over 85% Alignment Fraction). Complete vOTUs were divided into two groups based on whether they were predicted, by CheckV, to be proviruses (*i.e.*, their genomes were flanked by regions of bacterial DNA [[1]](https://paperpile.com/c/1tcawA/0FJA)) or not. While CheckV was designed to trim flanking bacterial regions from viral contigs, it was not intended to excise prophages from bacterial chromosomes ([berkeleylab / CheckV / issues / #78 - False positives or non-detected proviruses? — Bitbucket](https://bitbucket.org/berkeleylab/checkv/issues/78/false-positives-or-non-detected-proviruses)). It is possible that several putative viral contigs were not efficiently extracted from the bacterial DNA sequences in which they were initially identified. Consistent with this, the proviruses group had a significantly higher number of host genes (Pearson’s chi-squared test, *p* < 2.2e-16) and HVR (Wilcoxon test, *p* < 2.2e-16) compared to non-proviruses (**Additional Figure 1a**).


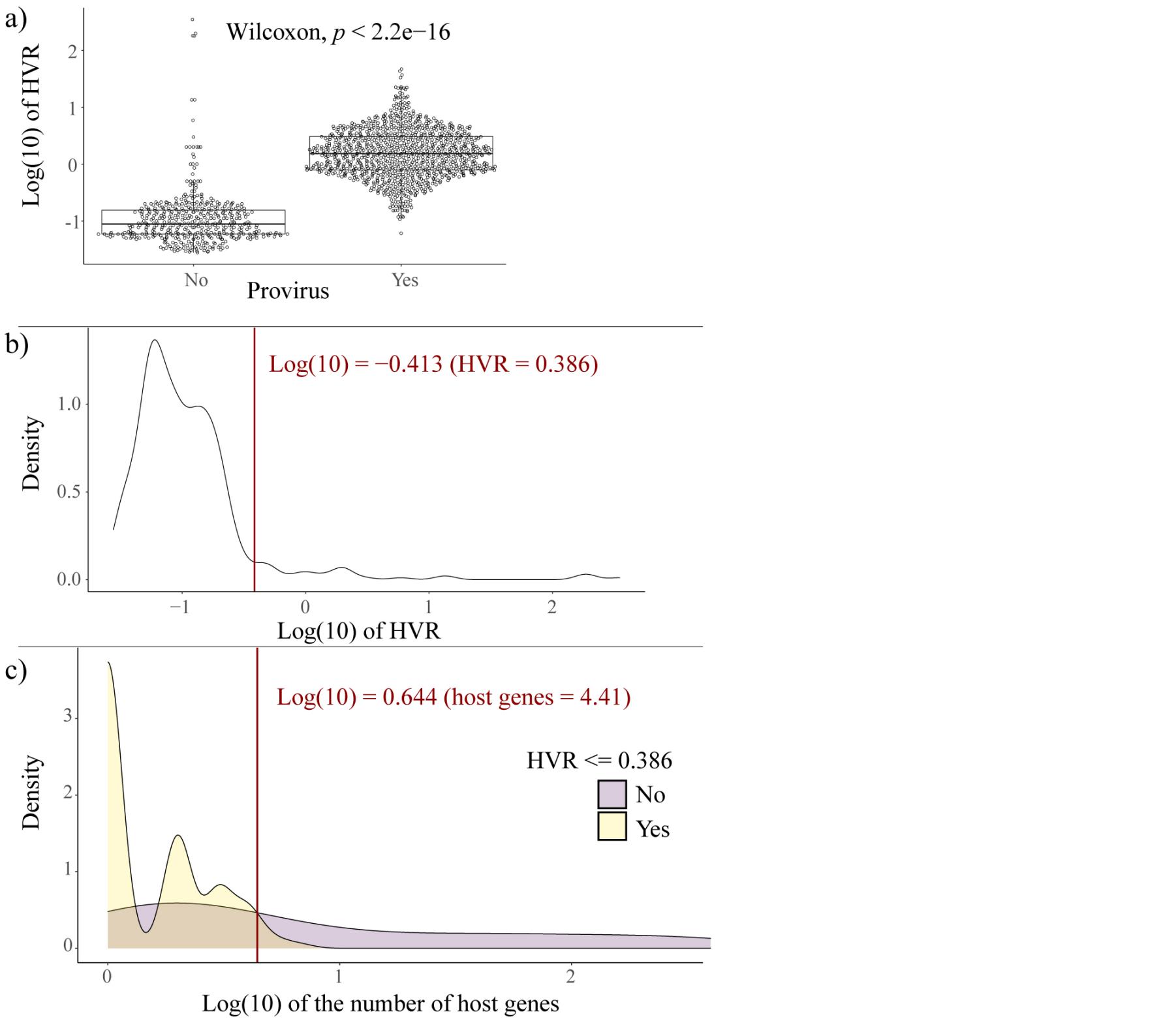


**Additional Figure 1** Selection of the Host-to-Viral gene ratio (HVR) cut-off to remove false positive putative viral contigs **a)** Proviral sequences exhibited a significantly higher HVR compared to non-proviral sequences. Each dot represents the HVR of a putative viral contig. **b)** The majority of complete non-proviral putative viral contigs had an HVR ≤ 0.386 (red vertical line). **c)** The majority of complete non-proviral putative viral contigs with an HVR ≤ 0.386 carried a number of host genes ≤ 4.41 (red vertical line).

Due to the likelihood of predicted proviral contigs to include bacterial DNA regions, only non-proviral complete sequences were used to determine the threshold of host genes and HVR to reduce the chances of calling false positive contigs. The HVR of non-proviral complete sequences exhibited a right-skewed distribution with a right tail that started at HVR = 0.386 (red line in **Additional Figure 1b**). Most contigs (96.79%) had an HVR value ≤ 0.386, and this HVR value was chosen as the cut-off used in this study to call false positives. Only 7.87% of complete proviral contigs were included under the HVR threshold, resulting in the majority of predicted proviruses being excluded (92.13%). By drastically reducing the number of proviruses, it is possible to introduce biases against phages that undergo the lysogenic cycle (*i.e.*, temperate phages). Nonetheless, there was no significant difference between the lifestyles of the putative viral contigs that met the HVR inclusion criteria (*i.e.*, HVR ≤ 0.386) and the entire set of putative viral contigs (Pearson’s Chi-squared test, *p* = 0.32). Consequently, we contend that minimal biases were introduced in this analysis.

The number of host genes carried by putative non-proviral complete contigs that were included or excluded by the HVR threshold (HVR = 0.386) were plotted in a density plot (**Additional Figure 1c**). The point at which the two distributions started to differ was chosen as the host genes cut-off (n = 4) and used to determine whether a contig was viral or not (Log(10) = 0.644, or host genes = 4.41) (red line in **Additional Figure 1c**).

# Identification of clonal UViGs

Clonal UViGs were identified based on genetic similarities (99.4% ANI over 85% AF) between UViGs detected in the recipients following the transplant and UViGs from the donors. The ANI cut-off value was determined by assessing the ANI distribution for UViGs clustering in a vOTU, dependent on whether the vOTU was identified in the same individual (*i.e.*, multiple stool donations from the same GBT donor) or from different individuals (*i.e.,* different GBT donors) (**Additional Figure 2**).


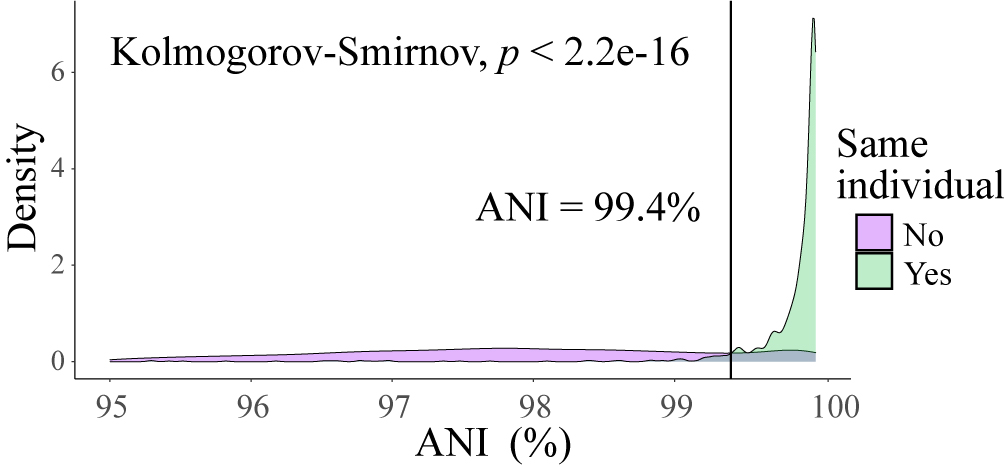


**Additional Figure 2**. Selection of Average Nucleotide Identity (ANI) cut-off to identify clonal UViGs. The statistical significance of the difference in ANI distribution was assessed using a Kolmogorov-Smirnov test. The black line represents the ANI cut-off value (99.4%), above which UViGs specific to individuals were overrepresented.

The ANI value above which UViGs specific to individuals were overrepresented (*i.e.*, ≥ 99.4%) was chosen as the cut-off to identify clonal UViGs engrafted from the donors to the FMT recipients. It is important to note that, despite a significant difference (Kolmogorov-Smirnov test, *p* < 2.2e-16) in the ANI distributions of UViGs depending on whether these were present in one or multiple individuals, there was still an overlap between the two distributions (**Additional Figure 2**). Specifically, 15.7% of UViG from different individuals clustered into vOTUs with an ANI ≥ 99.4%, while 5.4% of UViGs from the same individual at different time points clustered into vOTUs with an ANI < 99.4%. Therefore, a cut-off of 99.4% ANI has likely resulted in the exclusion of 5.4% false negatives and the inclusion of 15.7% false positives.

# UViGs characterization

The taxonomy of the UViGs was determined by combining information from vContact2 (*v*0.9.19) [[11, 12]](https://paperpile.com/c/1tcawA/YA5ko+a3tui) and Hidden Markov Models (HMM) profile similarities between the vOTUs and a viral orthologous gene database, ViPhOGs (29/06/2021) [[13]](https://paperpile.com/c/1tcawA/RB5az). The Open Reading Frames (ORFs) in the UViGs were predicted using Prodigal (*v*2.6.3) [[14]](https://paperpile.com/c/1tcawA/9vv0e) with default parameters. ORFs are portions of the genome included between translation start and stop codons, which likely represent protein-coding regions. UViGs with genome size ≥ 10 kb were used as input for vContact2 (*v*0.9.19) and were prepared for the analysis using the vContact2 (*v*0.9.19) function *vcontact2_gene2genome -s 'Prodigal-FAA'*. The taxonomy was predicted against RefSeq (release 201) database [[15]](https://paperpile.com/c/1tcawA/3JAWd) (*vcontact2 --raw-proteins --rel-mode 'Diamond' --db 'ProkaryoticViralRefSeq201-Merged' --pcs-mode MCL --vcs-mode ClusterONE --c1-bin ~vContact2/bin/cluster_one-1.0.jar*). All the UViGs, including the ones with genomes size < 10kb, were also taxonomically classified by alignment against the ViPhOGs database, as described previously [[16]](https://paperpile.com/c/1tcawA/Xa9Zz). Briefly, ViPhOGs HMM profiles were compressed into binary datafiles using the function *hmmpress* within hmmer (*v*3.3.2) [[17]](https://paperpile.com/c/1tcawA/zlelY) using default parameters [[18]](https://paperpile.com/c/1tcawA/48uZ0)*.* Alignments between query proteins and the ViPhOGs profiles were performed with hmmer (*v*3.3.2) using the function *hmmscan* (*hmmscan -E 0.001 --domE 0.1 --tblout*). UViGs were taxonomically classified if ≥ 20% of genes had significant hits on ViPhOGs (*i.e.,* sequence *e-value* ≤ 0.001 and domain-independent *e-value* ≤ 0.1), and ≥ 60% of those genes were assigned to the same viral taxon. If the vOTU had < 10 genes, at least two genes with significant hits were required. To taxonomically classify UViGs as Crass-like phages, the UViGs were clustered using vContact2 (*v*0.9.19) with 673 Crass-like sequences collected by Yutin et al [[19]](https://paperpile.com/c/1tcawA/ymlsS) *(vcontact2 --raw-proteins --rel-mode 'Diamond' --db 'None' --pcs-mode MCL --vcs-mode ClusterONE --c1-bin ~vContact2/bin/cluster_one-1.0.jar*). The outputs of all the approaches were considered when describing the taxonomy of an UViG.

# Statistical analyses

All the statistical tests were performed in R (*v*4.0.3).

Pairwise comparisons between two different categorical variables were performed using Wilcoxon rank-sum tests (‘wilcoxon.test()’ function from the stats package, built-in in R (*v*4.3.0)). Paired or not paired Wilcoxon rank-sum tests were used for pairwise comparisons between dependent or independent data, respectively. The comparisons performed were: 1) relative abundance of engrafted and non-engrafted vOTUs in the donors (not paired); 2) differences in beta diversity from 6 weeks at 12 and 26 weeks between the stable and fluctuating component of the recipients phageomes (paired); 3) differences in beta diversity from 6 weeks at 12 and 26 weeks between FMT and placebo recipients of the entire phageome (not paired). The *p*-values were adjusted using the Benjamini-Hochberg method of correction, or False Discovery Rate (FDR), using the ‘p.adjust()’ function from the stats package. Pearson’s Chi-squared (χ²) tests, calculated using the function ‘chisq.test()’ from the stats package, were used to determine the statistical significance of differences in: 1) phageome composition, between FMT and placebo recipients, stratified based on vOTUs origins, *i.e.*, shared with donors, conserved from baseline, novel from 6, 12, and 26 weeks; 2) donor’s engraftment efficacy; 3) taxonomy, host specificity, and lifestyle between engrafted and non-engrafted vOTUs; 4) stability of vOTUs between FMT and placebo recipients. Significant differences calculated using the χ² test were further analyzed via post hoc testing using the ‘chisq.posthoc.test’ function from the chisq.posthoc.test package (276).

Differences between multiple categorical variables were assessed by Kruskal-Wallis tests, using the ‘kruskal.test()’ function from the stats package. Kruskal-Wallis tests were used to determine the statistical significance of the differences in phageome alpha diversity between donors.

Linear correlations were assessed with Pearson’s correlation using the ‘stat_cor()’ extension for the ‘ggplot()’ function from the ggpubr (274) and tidyverse (275) packages, respectively. Pearson’s correlations were performed on normally distributed data using the flag *method = “Pearson”*. The normality of the data was assessed with the ‘shapiro.test()’ function from the stats package. Linear correlation was performed between donor engraftment efficacy and their phageome alpha diversity.

The differences between the ANI distributions of UViGs clustering in vOTUs, whether they were identified in the same donor at different donations or in different donors, were assessed with Kolmogorov-Smirnov test (‘ks.test()’ function from the stats package).

The FMT-promoted shifts in phageome composition from baseline within each individual were assessed using linear mixed-effects models (‘lmer()’ function from the lme4 package). The *p*-values were visualized in the ‘lmer()’ output with the lmerTest package. The linear mixed-effects models incorporated the fixed effects of time points (with baseline as the reference) and treatment (using placebo as the reference), while considering the recipients as random effects *(lmer( ~ time points * treatment + (1|recipient))*. Linear mixed-effects models were used to assess: 1) the shift from baseline in beta diversity between recipients and individual donors. The statistical significance of the shift was calculated separately for each donor. 2) the shift in alpha diversity from baseline. The statistical significance of the shift was calculated separately for each sex. 3) the shift in temperate and virulent phages’ total abundance from baseline. The statistical significance of the shift was calculated separately for each sex and lifestyle category (*i.e.,* temperate and virulent). For each linear mixed-effects model, the *p*-values were collectively adjusted with the FDR method of correction.

# References

[1. Nayfach S, Camargo AP, Schulz F, Eloe-Fadrosh E, Roux S, Kyrpides NC. CheckV assesses the quality and completeness of metagenome-assembled viral genomes. Nat Biotechnol. 2020. https://doi.org/](http://paperpile.com/b/1tcawA/0FJA)[10.1038/s41587-020-00774-7](http://dx.doi.org/10.1038/s41587-020-00774-7)[.](http://paperpile.com/b/1tcawA/0FJA)

[2. Guo J, Vik D, Adjie Pratama A, Roux S, Sullivan M. Viral sequence identification SOP with VirSorter2 v3. 2021.](http://paperpile.com/b/1tcawA/kPRAg)

[3.](http://paperpile.com/b/1tcawA/Af6ur) [VirSorter2 - jiarong.](http://paperpile.com/b/oADQaX/LYrC) <https://github.com/jiarong/VirSorter2>[.](http://paperpile.com/b/oADQaX/LYrC) Accessed 6 Dic 2022

[4. Guo J, Bolduc B, Zayed AA, Varsani A, Dominguez-Huerta G, Delmont TO, et al. VirSorter2: a multi-classifier, expert-guided approach to detect diverse DNA and RNA viruses. Microbiome. 2021;9:37.](http://paperpile.com/b/1tcawA/OxNUL)

[5. Sausset R, Petit MA, Gaboriau-Routhiau V, De Paepe M. New insights into intestinal phages. Mucosal Immunol. 2020;13:205–15.](http://paperpile.com/b/1tcawA/ysKpg)

[6. Kirchberger PC, Ochman H. Resurrection of a Global, Metagenomically Defined Microvirus.](http://paperpile.com/b/1tcawA/BzITo) *eLife*. 2020; 9:e51599.

[7.](http://paperpile.com/b/1tcawA/Zpnzo) [CheckV - Berkeley Lab.](http://paperpile.com/b/oADQaX/ZIQB) <https://bitbucket.org/berkeleylab/CheckV.> [Accessed 6 Dic 2022.](http://paperpile.com/b/oADQaX/ZIQB)

[8. Roux S, Adriaenssens EM, Dutilh BE, Koonin EV, Kropinski AM, Krupovic M, et al. Minimum Information about an Uncultivated Virus Genome (MIUViG). Nat Biotechnol. 2019;37:29–37.](http://paperpile.com/b/1tcawA/C2YBh)

[9. Jain C, Rodriguez-R LM, Phillippy AM, Konstantinidis KT, Aluru S. High throughput ANI analysis of 90K prokaryotic genomes reveals clear species boundaries. Nat Commun. 2018;9:5114.](http://paperpile.com/b/1tcawA/Tfta1)

[10. Kauffman KM, Chang WK, Brown JM, Hussain FA, Yang J, Polz MF, et al. Resolving the structure of phage–bacteria interactions in the context of natural diversity. Nat Commun. 2022;13:1–20.](http://paperpile.com/b/1tcawA/djomP)

[11. Bin Jang H, Bolduc B, Zablocki O, Kuhn JH, Roux S, Adriaenssens EM, et al. Taxonomic assignment of uncultivated prokaryotic virus genomes is enabled by gene-sharing networks. Nat Biotechnol. 2019;37:632–9.](http://paperpile.com/b/1tcawA/YA5ko)

[12.](http://paperpile.com/b/1tcawA/a3tui) [Vcontact2 - MAVERICLab.](http://paperpile.com/b/oADQaX/TB6S) <https://bitbucket.org/MAVERICLab/vcontact2/src/master/>. Accessed 6 Dic 2022

[13. Moreno-Gallego JL, Reyes A. Informative Regions In Viral Genomes. Viruses. 2021;13.](http://paperpile.com/b/1tcawA/RB5az)

[14. Hyatt D, Chen GL, LoCascio PF, Land ML, Larimer FW, Hauser LJ. Prodigal: Prokaryotic gene recognition and translation initiation site identification. BMC Bioinformatics. 2010;11.](http://paperpile.com/b/1tcawA/9vv0e)

[15. O’Leary NA, Wright MW, Brister JR, Ciufo S, Haddad D, McVeigh R, et al. Reference sequence (RefSeq) database at NCBI: current status, taxonomic expansion, and functional annotation. Nucleic Acids Res. 2016;44:D733–45.](http://paperpile.com/b/1tcawA/3JAWd)

[16. Camarillo-Guerrero LF, Almeida A, Rangel-Pineros G, Finn RD, Lawley TD. Massive expansion of human gut bacteriophage diversity. Cell. 2021;184:1098–109.e9.](http://paperpile.com/b/1tcawA/Xa9Zz)

[17. Eddy SR. Accelerated Profile HMM Searches. PLoS Comput Biol. 2011;7:e1002195.](http://paperpile.com/b/1tcawA/zlelY)

[18.](http://paperpile.com/b/1tcawA/48uZ0) [Hmmer - EddyRivasLab.](http://paperpile.com/b/oADQaX/cvIy) <https://github.com/EddyRivasLab/hmmer>. Accessed 6 Dic 2022.

[19. Yutin N, Benler S, Shmakov SA, Wolf YI, Tolstoy I, Rayko M, et al. Analysis of metagenome-assembled viral genomes from the human gut reveals diverse putative CrAss-like phages with unique genomic features. Nat Commun. 2021;12:1044.](http://paperpile.com/b/1tcawA/ymlsS)

[20.](http://paperpile.com/b/1tcawA/yrk2Q) [VirHostMatcher-Net - WeiliWw.](http://paperpile.com/b/oADQaX/cqRj) [https://github.com/WeiliWw/
VirHostMatcher-Net](https://github.com/WeiliWw/VirHostMatcher-Net)[.](http://paperpile.com/b/oADQaX/cqRj) Accessed 6 Dic 2022.

[21. Wang W, Ren J, Tang K, Dart E, Ignacio-Espinoza JC, Fuhrman JA, et al. A network-based integrated framework for predicting virus–prokaryote interactions. NAR Genom Bioinform. 2020;2.](http://paperpile.com/b/1tcawA/YN9AC)

[22. Hockenberry AJ, Wilke CO. BACPHLIP: predicting bacteriophage lifestyle from conserved protein domains. PeerJ. 2021;9:e11396.](http://paperpile.com/b/1tcawA/g4yDf)

[23.](http://paperpile.com/b/1tcawA/hJCLC) [Bacphlip](http://paperpile.com/b/oADQaX/NAmn) - adamhockneberry. <https://github.com/adamhockenberry/bacphlip>[.](http://paperpile.com/b/oADQaX/NAmn) Accessed 6 Dic 2022
