# Supplementary material for: Fecal microbiota transplantation alters gut phage communities in a clinical trial for obesity
Source: Microbiome. 2024 Jul 6;12:122. doi: 10.1186/s40168-024-01833-w (PMC11227244; doi:10.1186/s40168-024-01833-w)
Supplement: Supplementary file 3 — Additional file 2. Additional results. In this file are reported additional results that were mentioned in the main text: 1) Output of the phage identification workflow; 2) Analysis of the engrafted vOTUs; 3) The gut phageome comprised of a stable and a fluctuating component. [file 40168_2024_1833_MOESM2_ESM.docx]

Additional File 2

# Output of the phage identification workflow

381 stool samples were collected from the participants in the GBT (donors and recipients), generating, on average, 23 million reads per sample (6.9 Gb/sample). The reads were assembled into 36,806,474 contigs using MegaHIT (*v*1.1.4), which were analyzed with VirSorter2 (*v*2.2.3) and CheckV (*v*0.7.0) to identify the putative viral contigs, according to a modified version of the SOP proposed by Guo et al [[1]](https://paperpile.com/c/e0WsrP/FlI9d). A total number of 107,709 (0.29%) contigs were predicted to be putative viral contigs. 14,232 putative viral contigs (13.2% of all putative viral contigs) were considered false positives, based on their HVR (Host-to-viral genes ratio) and number of host genes, and therefore removed from the analysis. The remaining 93,477 putative viral contigs, referred to as UViGs in this analysis according to MIUViG standards [[2]](https://paperpile.com/c/e0WsrP/atizn), were clustered into vOTUs (95% ANI over 85% AF [[2]](https://paperpile.com/c/e0WsrP/atizn)), producing 25,805 unique vOTUs, of which 15,020 were singletons (58.2%). The longest UViG of each vOTU was considered as the representative UViG of the vOTU. Of the 25,805 vOTUs, 1761 (6.8%) were represented by an UViG with completeness ≥ 90% (*i.e.,* high-quality draft genome [[2]](https://paperpile.com/c/e0WsrP/atizn)) (**Additional Table 1**) (**Additional Figure 3a**). vOTUs represented by an UViG with completeness < 90% (*i.e.,* genome fragments [[2]](https://paperpile.com/c/e0WsrP/atizn)) were discarded.

On average, 17.96 ± 3.32% of total reads per sample aligned against the phage UViGs obtained in the analysis (Additional Figure 4).


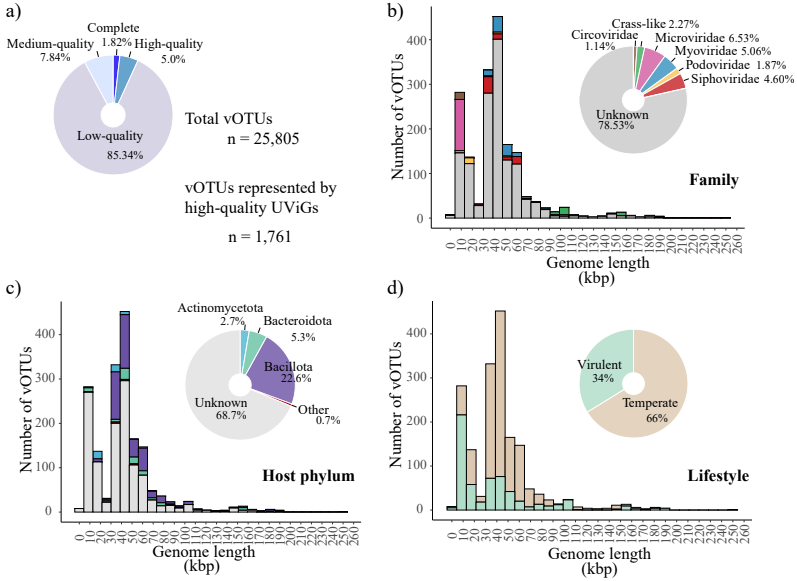
**Additional Figure 3**. **Characterization of the vOTU-representative UViGs obtained from the participants in the Gut Bugs Trial.** **a)** CheckV quality of the vOTU-representative UViGs. **b)** Taxonomic annotation (Family) of the vOTU-representative high-quality vOTU-representative UViGs. **c)** Host specificity (Phylum) of the high-quality vOTU-representative UViGs. **d)** Lifestyle of the high-quality vOTU-representative UViGs.


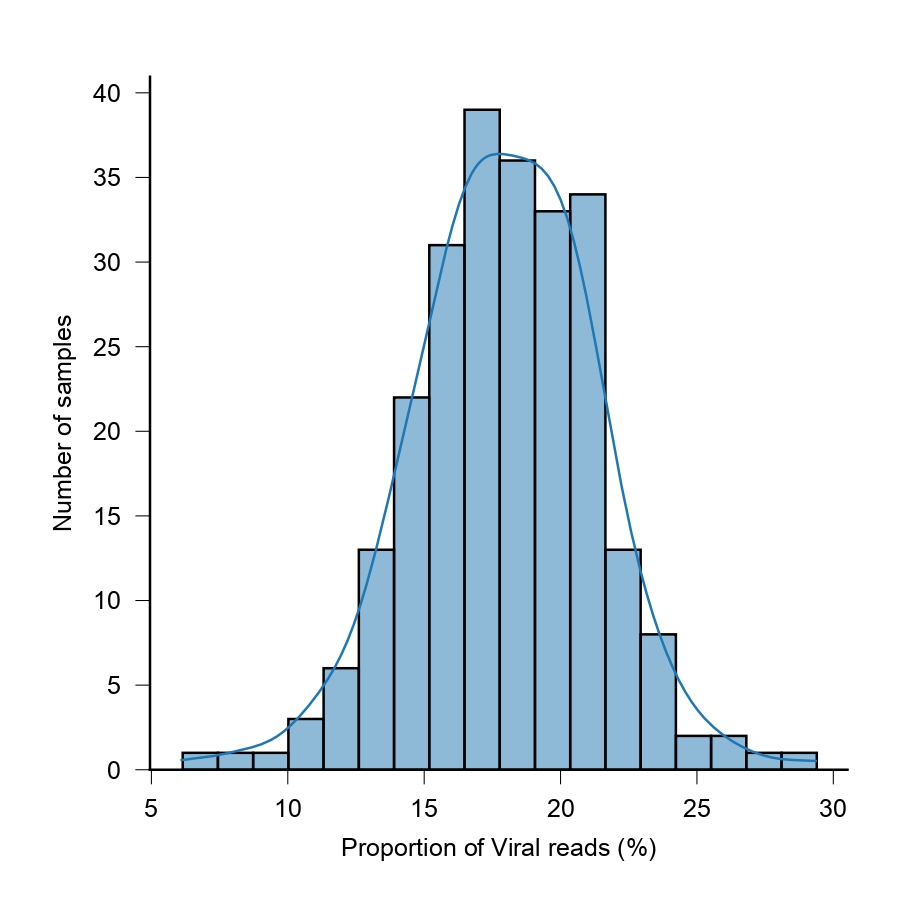


Additional Figure 4. Proportion of reads per sample aligning against the viral gene catalog.

The high-quality draft genome vOTUs were characterized by determining the genome length, taxa (Family), the host specificity (Phylum), and the lifestyle of the representative UViG (**Additional Figure 3b, c, d**). The vOTUs varied in length, ranging from 4,104 bp to 253,960 bp, with the majority (50%) of the vOTUs included within 26,620 and 51,547 bp. The majority of the vOTUs could not be assigned to any known taxa (78.53%), while the remainder belonged to the phage families *Microviridae* (6.53%), *Myoviridae* (5.06%), *Siphoviridae* (4.60%), Crass*-*like (2.27%), *Podoviridae* (1.87%), and to the eukaryotic viral family *Circoviridae* (1.14%) (**Additional Figure 3b**). The vOTUs were predicted to infect the bacterial phyla *Bacillota* (22.6%), *Bacteroidota* (5.3%), *Actinomycetota* (2.7%), *Pseudomonota* (0.62%), and the archaea phylum *Euryarchaeota* (0.06%). A bacterial host could not be predicted for 68.7% of the vOTUs. The last two phyla were reported as Other in the (**Additional Figure 3c**). The majority of the vOTUs (66%) were predicted to be temperate (34% virulent) (**Additional Figure 3d**).

# Analysis of the engrafted vOTUs

The majority of the engrafted vOTUs could not be taxonomically classified at a family level (n = 234, 83%) (**Additional Figure 5a**).


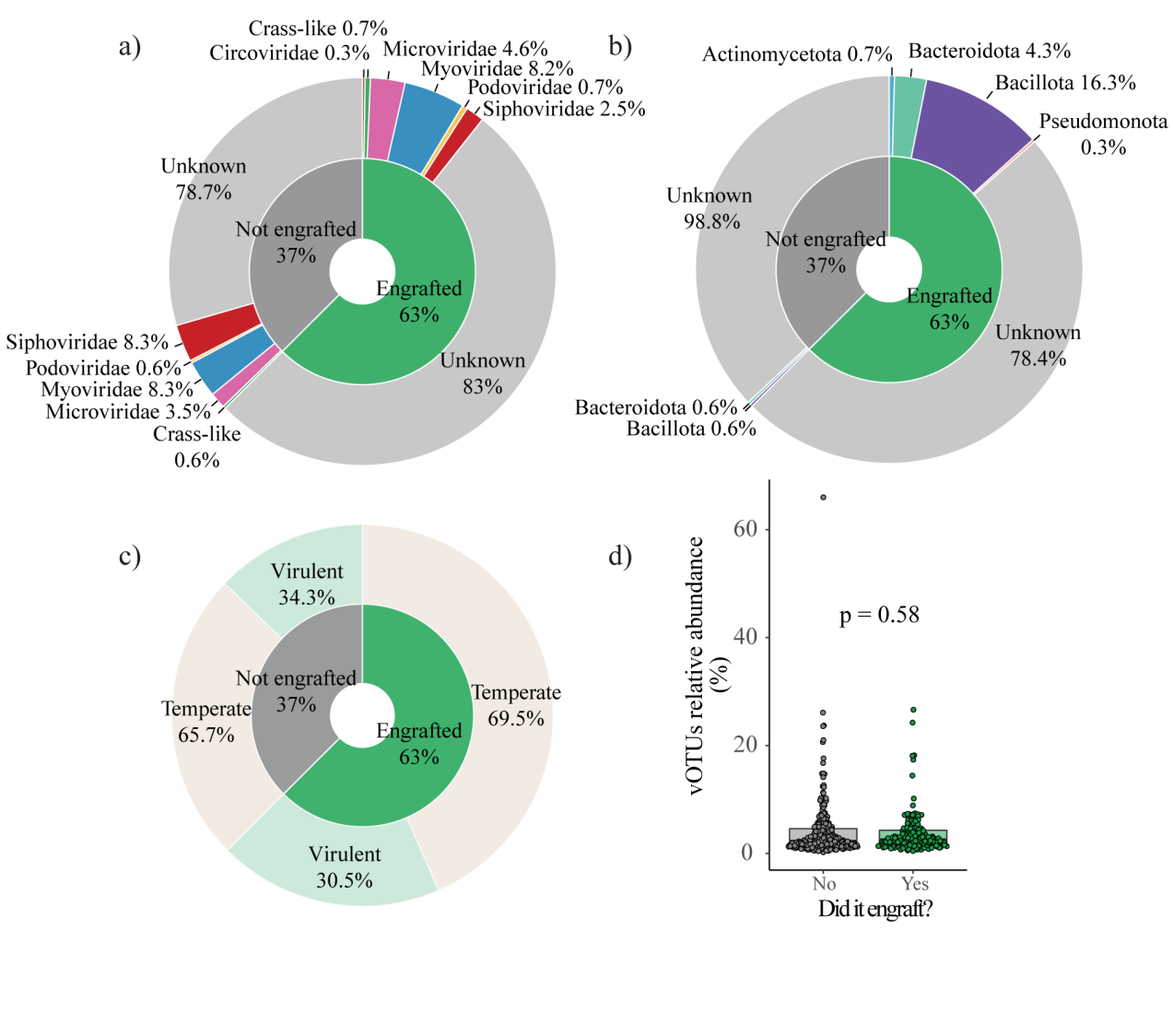


**Additional Figure 5. A bacterial host could be predicted more frequently for engrafted vOTUs than for non-engrafted vOTUs.** The differences between engrafted and non-engrafted vOTUs identified in the donors were assessed with regards to **a)** taxonomy (Family), **b)** host specificity (Phylum), **c)** lifestyle, and **d)** relative abundance (each dot represents a unique vOTU). The statistical significance of the difference in relative abundance between engrafted and non-engrafted vOTUs was assessed using a Wilcoxon test.

The remaining vOTUs were classified as *Myoviridae* (n = 23, 8.2%), *Microviridae* (n = 13, 4.6%), *Siphoviridae* (n = 7, 2.5%), *Podoviridae* (n = 2, 0.7%), Crass-like phages (n = 2, 0.7%), and *Circoviridae* (n = 1, 0.3%). At a phylum level, the engrafted vOTUs were predicted to infect *Bacillota* (n = 46, 16.3%), *Bacteroidota* (n = 12, 4.3%), *Actinomycetota* (n = 2, 0.7%), and *Pseudomonota* (n = 1, 0.4%) (**Additional Figure 5b**). Nonetheless, the host specificity could not be determined for the majority of the vOTUs (n = 221, 78.4%). With respect to their lifestyle, the majority of the engrafted vOTUs were temperate (n = 196, 69.5%), while the rest were virulent (n = 86, 30.5%) (**Additional Figure 5c**).

The unique vOTUs identified in all the donors were compared based on whether they had been engrafted or not in order to identify any phage-specific characteristics (*i.e.,* taxonomy, host specificity, lifestyle, and relative abundance) that might have been associated with engraftment. The engrafted vOTUs did not differ from the non-engrafted vOTUs in either taxonomy (χ² test, *p* = 0.88) (**Additional Figure 5a**), lifestyle (χ² test, *p* = 0.46) (**Additional Figure 5c**), or average relative abundance (Wilcoxon test, *p* = 0.74) (**Additional Figure 5d**). There was a significant difference only in host specificity (χ² test, *p* = 1.9e^-7^) (**Additional Figure 5b**). Specifically, a significantly higher proportion of vOTUs for which the host specificity could be predicted were engrafted (post hoc analysis, *p* = 1.3e^-8^).

# The gut phageome comprised of a stable and a fluctuating component

Longitudinal analysis of the proportion of novel and conserved phages in GBT recipients revealed that these did not change throughout the course of the study, suggesting the existence of a stable fraction of the gut phageome and a fluctuating one. In support of this hypothesis, the stable component of the gut phageome (*i.e.,* vOTUs present from baseline or shared with the donors) showed a significantly lower variability compared to the fluctuating component (*i.e.,* novel vOTUs) at all time points (**Additional Figure 6**).


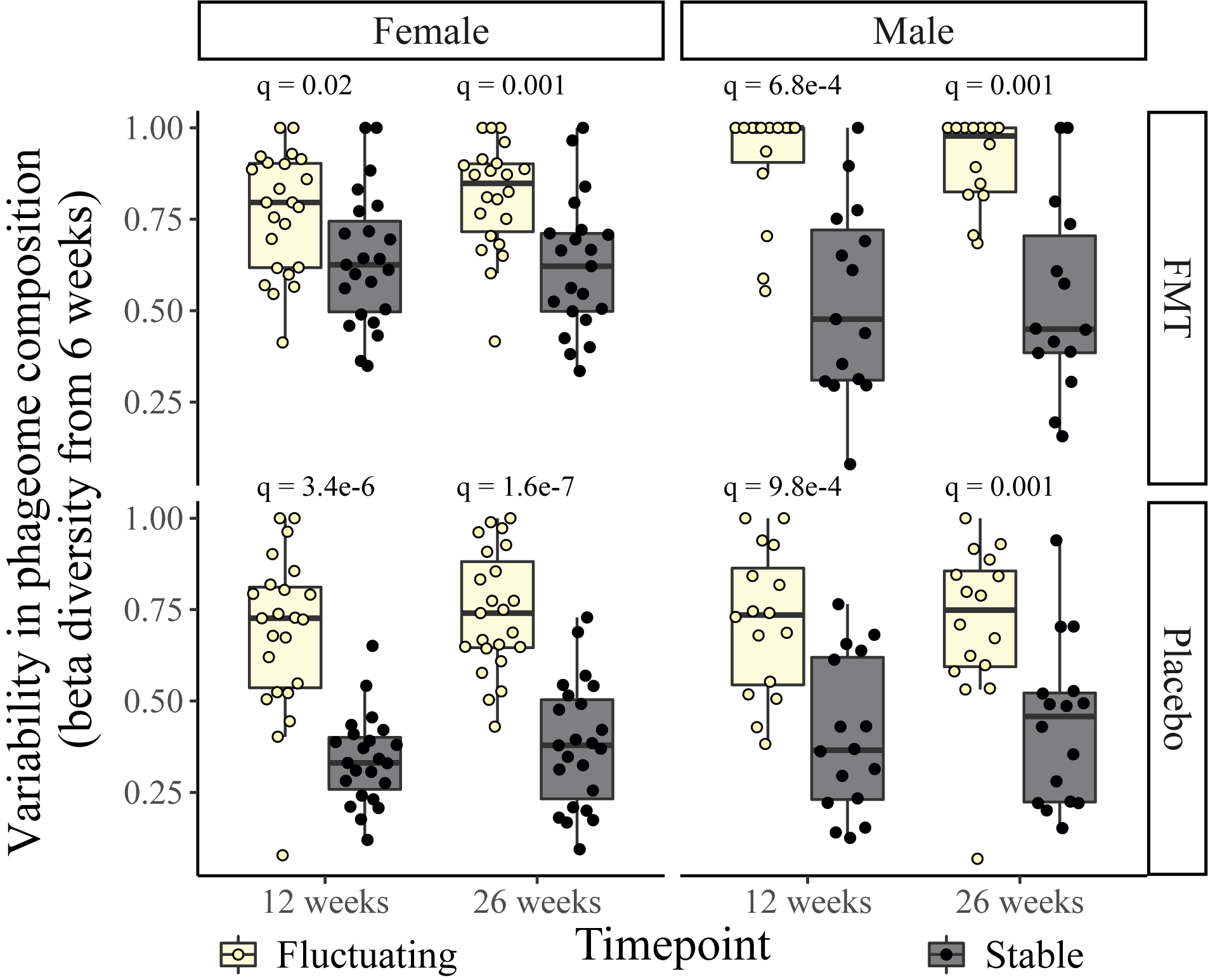


**Additional Figure 6.** **The fluctuating component of the gut phageome exhibited higher variability than the stable component**. Beta diversity was calculated with Bray-Curtis dissimilarities. Each dot represents the phageome of a single individual. Pairwise comparisons were calculated using paired Wilcoxon tests. The *p*-values were adjusted using the FDR method of correction.

# References

[1. Guo J, Vik D, Adjie Pratama A, Roux S, Sullivan M. Viral sequence identification SOP with VirSorter2 v3. 2021.](http://paperpile.com/b/e0WsrP/FlI9d)

[2. Roux S, Adriaenssens EM, Dutilh BE, Koonin EV, Kropinski AM, Krupovic M, et al. Minimum Information about an Uncultivated Virus Genome (MIUViG). Nat Biotechnol. 2019;37:29–37.](http://paperpile.com/b/e0WsrP/atizn)
