# Supplementary material for: Fecal microbiota transplantation alters gut phage communities in a clinical trial for obesity
Source: Microbiome. 2024 Jul 6;12:122. doi: 10.1186/s40168-024-01833-w (PMC11227244; doi:10.1186/s40168-024-01833-w)
Supplement: Supplementary file 4 — Additional file 3. Additional Table S1. Table reporting the metadata requested by MIUViG standards for the description of UViGs. In the first page are reported the MIUViG guidelines for the required metadata. In the second page are reported the metadata of the UViGs obtained in this study. In the following pages are reported the metadata of each vOTU-representative UViG analized in this study: 1) UViG name; 2) VirSorter2 score; 3) Predicted genome type; 4) whether it was identified as provirus or not by CheckV; 4) CheckV quality; 5) Completeness (%); 6) MIUViG quality; 7) Approach used by CheckV to determine completeness; 8) Taxonomy (Family); 9) Host specificity (Phylum); 10) Host specificity (Species). [file 40168_2024_1833_MOESM3_ESM.pdf]

Metadata category	Metadata	Requirement	Description	Syntax	Example value	Specificity to UViGs
**General genome metadata**	**source of UViGs**	Mandatory	Type of dataset from which the UViG was obtained	[metagenome (not viral targeted) | viral fraction metagenome (virome) | sequence-targeted metagenome | metatranscriptome (not viral targeted) | viral fraction RNA metagenome (RNA virome) | sequence-targeted RNA metagenome | microbial single amplified genome (SAG) | viral single amplified genome (vSAG) | isolate microbial genome | other]	viral fraction metagenome (virome)	New and specific to UViGs
	**assembly software**	Mandatory	Tool(s) used for assembly and/or binning, including version number and parameters	{software};{version};{parameters}	metaSPAdes; 3.11.0; kmer set 21,33,55,77,99,121, default parameters otherwise	Identical MIMAG / MISAG
	**viral identification software**	Mandatory	Tool(s) used for the identification of UViG as a viral genome, software or protocol name including version number, parameters, and cutoffs used (see Table S2)	{software};{version};{parameters}	VirSorter; 1.0.4; Virome database, category 2	New and specific to UViGs
	**predicted genome type**	Mandatory	Type of genome predicted for the UViG	[DNA | dsDNA | ssDNA | RNA | dsRNA | ssRNA | ssRNA (+) | ssRNA (-) | mixed | uncharacterized]	dsDNA	New and specific to UViGs
	**predicted genome structure**	Mandatory	Expected structure of the viral genome	[segmented | non-segmented | undetermined]	non-segmented	New and specific to UViGs
	**detection type**	Mandatory	Type of UViG detection	[independent sequence (UViG) | provirus (UpViG)]	independent sequence (UViG)	New and specific to UViGs
**Genome quality**	**assembly quality**	Mandatory	The assembly quality categories, specific for virus genomes, are based on sets of criteria as follows. **Finished**: Single, validated, contiguous sequence per replicon without gaps or ambiguities, with extensive manual review and editing to annotate putative gene functions and transcriptional units. **High-quality draft genome**: One or multiple fragments, totaling ≥ 90% of the expected genome or replicon sequence or predicted complete. **Genome fragment(s)**: One or multiple fragments, totalling < 90% of the expected genome or replicon sequence, or for which no genome size could be estimated.	[Finished genome | High-quality draft genome | Genome fragment(s)]	High-quality draft genome	Comparable to and adapted from MIMAG / MISAG
	**number of contigs**	Mandatory	Total number of contigs composing the UViG	{number}	1	Identical MIMAG / MISAG
	**completeness score**	Conditional (required for finished genomes and high-quality draft genomes, optional for other categories)	Estimated completeness of the UViG	{quality};{percentage}	high;92%	Comparable to and adapted from MIMAG / MISAG
	**completeness approach**	Conditional (required if a completeness estimation is provided)	Approach used to estimate the UViG completeness, including reference genome or group used, and contig feature suggesting a complete genome	{text}	UViG length compared to the average length of reference genomes from the P22virus genus (NCBI RefSeq v83)	Comparable to and adapted from MIMAG / MISAG
**Genome annotation**	**feature prediction**	Conditional (required if genome annotation is provided)	Method used to predict UViGs features such as ORFs, integration site, etc.	{software};{version};{parameters}	Prodigal; 2.6.3; default parameters	Comparable to and adapted from MIMAG / MISAG
	**reference database(s)**	Conditional (required if a viral-specific ORF annotation is provided)	List of database(s) used for ORF annotation, along with version number and reference to website or publication	{database};{version};{reference}	pVOGs; 5; http://dmk-brain.ecn.uiowa.edu/pVOGs/ Grazziotin et al. 2017 doi:10.1093/nar/gkw975	Comparable to and adapted from MIMAG / MISAG
	**similarity search method**	Conditional (required if a viral reference database is provided)	Tool used to compare ORFs with database, along with version and cutoffs used	{software};{version};{parameters}	HMMER3; 3.1b2; hmmsearch, cutoff of 50 on score	Comparable to and adapted from MIMAG / MISAG
	**taxonomic classification**	Conditional (required if a taxonomic classification is provided)	Method used for taxonomic classification, along with reference database used, classification rank, and thresholds used to classify new genomes	{text}	e.g. vConTACT vContact2 (references from NCBI RefSeq v83, genus rank classification, default parameters)	Comparable to and adapted from MIMAG / MISAG
	**vOTU classification approach**	Conditional (required if a vOTU classification is provided)	Cutoffs and approach used when clustering new UViGs in "species-level" vOTUs. Note that results from standard 95% ANI / 85% AF clustering should be provided alongside vOTUS defined from another set of thresholds, even if the latter are the ones primarily used during the analysis.	{ANI cutoff};{AF cutoff};{clustering method}	95% ANI;85% AF; greedy incremental clustering	New and specific to UViGs
	**vOTU sequence comparison approach**	Conditional (required if a vOTU classification is provided)	Tool and thresholds used to compare sequences when computing "species-level" vOTUs.	{software};{version};{parameters}	blastn; 2.6.0+; e-value cutoff: 0.001	New and specific to UViGs
	**vOTU database**	Conditional (required if a vOTU classification is provided)	Reference database (i.e. sequences not generated as part of the current study) used to cluster new genomes in "species-level" vOTUs, if any	{database};{version}	NCBI Viral RefSeq; 83	New and specific to UViGs
	**host prediction approach**	Conditional (required if a predicted host is provided)	Tool or approach used for host prediction	[provirus | host sequence similarity | CRISPR spacer match | kmer similarity | co-occurrence | combination | other]	CRISPR spacer match	New and specific to UViGs
	**host prediction estimated accuracy**	Conditional (required if a host prediction is provided, except for proviruses)	For each tool or approach used for host prediction, estimated false discovery rates should be included, either computed de novo or from the literature (see Table S4)	{text}	CRISPR spacer match: 0 or 1 mismatches, estimated 8% FDR at the host genus rank (Edwards et al. 2016 doi:10.1093/femsre/fuv048)	New and specific to UViGs
**viral SAG metadata**	**sorting technology**	Conditional (required for UViG obained from vSAGs)	Method used to sort/isolate cells or particles of interest	[flow cytometric cell sorting | microfluidics | lazer-tweezing | optical manipulation | micromanipulation | other]	flow cytometry cell sorting	Comparable to and adapted from MIMAG / MISAG
	**single cell or viral particle lysis approach**	Conditional (required for UViG obained from vSAGs)	Method used to free DNA from interior of the cell(s) or particle(s)	[chemical | enzymatic | physical | combination]	chemical	Comparable to and adapted from MIMAG / MISAG
	**single cell or viral particle lysis kit protocol**	Optional	Name of the kit or standard protocol used for cell(s) or particle(s) lysis	{text}	MagMAX™ Viral RNA Isolation Kit	Comparable to and adapted from MIMAG / MISAG
	**WGA amplification approach**	Conditional (required for UViG obained from vSAGs)	Method used to amplify genomic DNA in preparation for sequencing	[pcr based | mda based | none]	mda based	Identical MIMAG / MISAG
	**WGA amplification kit**	Optional	Kit used to amplify genomic DNA in preparation for sequencing	{text}	REPLI-g Mini Kit	Identical MIMAG / MISAG
**viral MAG metadata**	**size fraction selected**	Conditional (required for UViG assembled from metagenomes)	Filtering pore size used in sample preparation	{float}-{float} {unit}	0-0.22 µm	New and specific to UViGs
	**virus enrichment approach**	Conditional (required for UViG assembled from metagenomes)	List of approaches used to enrich the sample for viruses, if any	[filtration | ultrafiltration | centrifugation | ultracentrifugation | PEG Precipitation | FeCl Precipitation | CsCl density gradient | DNAse | RNAse | targeted sequence capture | other | none]	filtration + FeCl Precipitation + ultracentrifugation + DNAse	New and specific to UViGs
	**nucleic acid extraction**	Conditional (required for UViG assembled from metagenomes)	A link to a literature reference, electronic resource or a standard operating procedure (SOP), that describes the material separation to recover the nucleic acid fraction from a sample	{PMID} | {DOI} | {URL}	10.1111/j.1462-2920.2012.02836.x	Identical MIMAG / MISAG
	**WGA amplification approach**	Conditional (required for UViG assembled from metagenomes)	Description of the approach used for whole genome amplification, if any	[pcr based | mda based | none]	none	Identical MIMAG / MISAG
	**binning parameters**	Conditional (required if genome bin(s) were defined)	The parameters that have been applied during the extraction of genomes from metagenomic datasets	[homology search | kmer | coverage | codon usage | combination]	kmer and coverage	Identical MIMAG / MISAG
	**binning software**	Conditional (required if genome bin(s) were defined)	Tool(s) used for the extraction of genomes from metagenomic datasets	[metabat | maxbin | concoct | groupm | esom | metawatt | combination | other]	metabat	Identical MIMAG / MISAG
	**reassembly post binning**	Conditional (required if genome bin(s) were defined)	Has an assembly been performed on a genome bin extracted from a metagenomic assembly?	[yes | no]	yes	Identical MIMAG / MISAG
	**MAG coverage software**	Optional	Tool(s) used to determine the genome coverage if coverage is used as a binning parameter in the extraction of genomes from metagenomic datasets	[bwa | bbmap | bowtie | other]	bowtie	Identical MIMAG / MISAG

Metadata category	Metadata	Requirement	Description	Syntax	Example value	Specificity to UViGs	UViGs in this study
**General genome metadata**	**source of UViGs**	Mandatory	Type of dataset from which the UViG was obtained	[metagenome (not viral targeted) | viral fraction metagenome (virome) | sequence-targeted metagenome | metatranscriptome (not viral targeted) | viral fraction RNA metagenome (RNA virome) | sequence-targeted RNA metagenome | microbial single amplified genome (SAG) | viral single amplified genome (vSAG) | isolate microbial genome | other]	viral fraction metagenome (virome)	New and specific to UViGs	viral fraction metagenome (virome)
	**assembly software**	Mandatory	Tool(s) used for assembly and/or binning, including version number and parameters	{software};{version};{parameters}	metaSPAdes; 3.11.0; kmer set 21,33,55,77,99,121, default parameters otherwise	Identical MIMAG / MISAG	Flye; v.2.9, --meta
	**viral identification software**	Mandatory	Tool(s) used for the identification of UViG as a viral genome, software or protocol name including version number, parameters, and cutoffs used (see Table S2)	{software};{version};{parameters}	VirSorter; 1.0.4; Virome database, category 2	New and specific to UViGs	VirSorter2; 2.2.3; --min-score 0.5, --min-length 4000; CheckV; 0.7.0, viral genes > 0 or viral genes = 0 & host genes = 0 or VirSorter2 score = 0.95 or hallmark genes > 2.
	**predicted genome type**	Mandatory	Type of genome predicted for the UViG	[DNA | dsDNA | ssDNA | RNA | dsRNA | ssRNA | ssRNA (+) | ssRNA (-) | mixed | uncharacterized]	dsDNA	New and specific to UViGs	dsDNA
	**predicted genome structure**	Mandatory	Expected structure of the viral genome	[segmented | non-segmented | undetermined]	non-segmented	New and specific to UViGs	undetermined
	**detection type**	Mandatory	Type of UViG detection	[independent sequence (UViG) | provirus (UpViG)]	independent sequence (UViG)	New and specific to UViGs	Independent sequence (UViG)
**Genome quality**	**assembly quality**	Mandatory	The assembly quality categories, specific for virus genomes, are based on sets of criteria as follows. **Finished**: Single, validated, contiguous sequence per replicon without gaps or ambiguities, with extensive manual review and editing to annotate putative gene functions and transcriptional units. **High-quality draft genome**: One or multiple fragments, totaling ≥ 90% of the expected genome or replicon sequence or predicted complete. **Genome fragment(s)**: One or multiple fragments, totalling < 90% of the expected genome or replicon sequence, or for which no genome size could be estimated.	[Finished genome | High-quality draft genome | Genome fragment(s)]	High-quality draft genome	Comparable to and adapted from MIMAG / MISAG	reported in next sheet
	**number of contigs**	Mandatory	Total number of contigs composing the UViG	{number}	1	Identical MIMAG / MISAG	1
	**completeness score**	Conditional (required for finished genomes and high-quality draft genomes, optional for other categories)	Estimated completeness of the UViG	{quality};{percentage}	high;92%	Comparable to and adapted from MIMAG / MISAG	reported in next sheet
	**completeness approach**	Conditional (required if a completeness estimation is provided)	Approach used to estimate the UViG completeness, including reference genome or group used, and contig feature suggesting a complete genome	{text}	UViG length compared to the average length of reference genomes from the P22virus genus (NCBI RefSeq v83)	Comparable to and adapted from MIMAG / MISAG	reported in next sheet
**Genome annotation**	**feature prediction**	Conditional (required if genome annotation is provided)	Method used to predict UViGs features such as ORFs, integration site, etc.	{software};{version};{parameters}	Prodigal; 2.6.3; default parameters	Comparable to and adapted from MIMAG / MISAG	Prodigal; 2.6.3; default parameters
	**reference database(s)**	Conditional (required if a viral-specific ORF annotation is provided)	List of database(s) used for ORF annotation, along with version number and reference to website or publication	{database};{version};{reference}	pVOGs; 5; http://dmk-brain.ecn.uiowa.edu/pVOGs/ Grazziotin et al. 2017 doi:10.1093/nar/gkw975	Comparable to and adapted from MIMAG / MISAG	
	**similarity search method**	Conditional (required if a viral reference database is provided)	Tool used to compare ORFs with database, along with version and cutoffs used	{software};{version};{parameters}	HMMER3; 3.1b2; hmmsearch, cutoff of 50 on score	Comparable to and adapted from MIMAG / MISAG	
	**taxonomic classification**	Conditional (required if a taxonomic classification is provided)	Method used for taxonomic classification, along with reference database used, classification rank, and thresholds used to classify new genomes	{text}	e.g. vConTACT vContact2 (references from NCBI RefSeq v83, genus rank classification, default parameters)	Comparable to and adapted from MIMAG / MISAG	vContact2 (References from NCBI RefSeq v201 and list of Crass-like phages https://doi.org/10.1038/s41467-021-21350-w, family rank classification, default parameters), HMM profile similarities (ViPhOGs database (29/06/2021), family rank classification, significant hits = sequence evalue <= 0.001 and domain evalue <= 0.1, >=20% of ORFs with significant hits and >=60% of those hits assigned to the same taxon
	**vOTU classification approach**	Conditional (required if a vOTU classification is provided)	Cutoffs and approach used when clustering new UViGs in "species-level" vOTUs. Note that results from standard 95% ANI / 85% AF clustering should be provided alongside vOTUS defined from another set of thresholds, even if the latter are the ones primarily used during the analysis.	{ANI cutoff};{AF cutoff};{clustering method}	95% ANI;85% AF; greedy incremental clustering	New and specific to UViGs	
	**vOTU sequence comparison approach**	Conditional (required if a vOTU classification is provided)	Tool and thresholds used to compare sequences when computing "species-level" vOTUs.	{software};{version};{parameters}	blastn; 2.6.0+; e-value cutoff: 0.001	New and specific to UViGs	vContact2; 0.9.19; default parameters. Hmmer; 3.3.2; sequence evalue <= 0.001 & domain evalue <= 0.1
	**vOTU database**	Conditional (required if a vOTU classification is provided)	Reference database (i.e. sequences not generated as part of the current study) used to cluster new genomes in "species-level" vOTUs, if any	{database}:{version}	NCBI Viral RefSeq; 83	New and specific to UViGs	NCBI Prokaryotic RefSeq; 201 ViPhOGs; (29/06/2021)
	**host prediction approach**	Conditional (required if a predicted host is provided)	Tool or approach used for host prediction	[provirus | host sequence similarity | CRISPR spacer match | kmer similarity | co-occurrence | combination | other]	CRISPR spacer match	New and specific to UViGs	VirHostMatcher-Net; 1.0
	**host prediction estimated accuracy**	Conditional (required if a host prediction is provided, except for proviruses)	For each tool or approach used for host prediction, estimated false discovery rates should be included, either computed de novo or from the literature (see Table S4)	{text}	CRISPR spacer match: 0 or 1 mismatches, estimated 8% FDR at the host genus rank (Edwards et al. 2016 doi:10.1093/femsre/fuv048)	New and specific to UViGs	VirHostMatcher-Net; Estimated 10% FDR at the host phylum level; https://doi.org/10.1093/nargab/lqaa044
**viral SAG metadata**	**sorting technology**	Conditional (required for UViG obained from vSAGs)	Method used to sort/isolate cells or particles of interest	[flow cytometric cell sorting | microfluidics | lazer-tweezing | optical manipulation | micromanipulation | other]	flow cytometry cell sorting	Comparable to and adapted from MIMAG / MISAG	
	**single cell or viral particle lysis approach**	Conditional (required for UViG obained from vSAGs)	Method used to free DNA from interior of the cell(s) or particle(s)	[chemical | enzymatic | physical | combination]	chemical	Comparable to and adapted from MIMAG / MISAG	
	**single cell or viral particle lysis kit protocol**	Optional	Name of the kit or standard protocol used for cell(s) or particle(s) lysis	{text}	MagMAX™ Viral RNA Isolation Kit	Comparable to and adapted from MIMAG / MISAG	
	**WGA amplification approach**	Conditional (required for UViG obained from vSAGs)	Method used to amplify genomic DNA in preparation for sequencing	[pcr based | mda based | none]	mda based	Identical MIMAG / MISAG	
	**WGA amplification kit**	Optional	Kit used to amplify genomic DNA in preparation for sequencing	{text}	REPLI-g Mini Kit	Identical MIMAG / MISAG	
**viral MAG metadata**	**size fraction selected**	Conditional (required for UViG assembled from metagenomes)	Filtering pore size used in sample preparation	{float}-{float} {unit}	0-0.22 µm	New and specific to UViGs	None
	**virus enrichment approach**	Conditional (required for UViG assembled from metagenomes)	List of approaches used to enrich the sample for viruses, if any	[filtration | ultrafiltration | centrifugation | ultracentrifugation | PEG Precipitation | FeCl Precipitation | CsCl density gradient | DNAse | RNAse | targeted sequence capture | other | none]	filtration + FeCl Precipitation + ultracentrifugation + DNAse	New and specific to UViGs	centrifugation + PEG precipitation + chloroform + DNAse
	**nucleic acid extraction**	Conditional (required for UViG assembled from metagenomes)	A link to a literature reference, electronic resource or a standard operating procedure (SOP), that describes the material separation to recover the nucleic acid fraction from a sample	{PMID} | {DOI} | {URL}	10.1111/j.1462-2920.2012.02836.x	Identical MIMAG / MISAG	
	**WGA amplification approach**	Conditional (required for UViG assembled from metagenomes)	Description of the approach used for whole genome amplification, if any	[pcr based | mda based | none]	none	Identical MIMAG / MISAG	
	**binning parameters**	Conditional (required if genome bin(s) were defined)	The parameters that have been applied during the extraction of genomes from metagenomic datasets	[homology search | kmer | coverage | codon usage | combination]	kmer and coverage	Identical MIMAG / MISAG	
	**binning software**	Conditional (required if genome bin(s) were defined)	Tool(s) used for the extraction of genomes from metagenomic datasets	[metabat | maxbin | concoct | groupm | esom | metawatt | combination | other]	metabat	Identical MIMAG / MISAG	
	**reassembly post binning**	Conditional (required if genome bin(s) were defined)	Has an assembly been performed on a genome bin extracted from a metagenomic assembly?	[yes | no]	yes	Identical MIMAG / MISAG	
	**MAG coverage software**	Optional	Tool(s) used to determine the genome coverage if coverage is used as a binning parameter in the extraction of genomes from metagenomic datasets	[bwa | bbmap | bowtie | other]	bowtie	Identical MIMAG / MISAG	

UViG	VirSorter2 score	Predicted genome type	Provirus	Completeness quality	Completeness (%)	Assembly quality	Completeness approach	Family	Host phylum	Host species
TM16_26wk_k141_14730	0.993	dsDNAphage	No	High-quality	100	High-quality draft genome	AAI-based (high-confidence)		*Bacteroidetes*	*Bacteroides uniformis*
TF37_6wk_k141_98120	0.987	dsDNAphage	No	High-quality	90.38	High-quality draft genome	AAI-based (high-confidence)		*Firmicutes*	*Blautia obeum*
TF10_6wk_k141_80791	1	dsDNAphage	No	Complete	100	High-quality draft genome	DTR (high-confidence)		*Firmicutes*	*Dorea longicatena*
TM36_BL_k141_9530	1	dsDNAphage	No	High-quality	97.03	High-quality draft genome	AAI-based (high-confidence)			
TF32_BL_k141_101121	0.993	dsDNAphage	No	High-quality	100	High-quality draft genome	AAI-based (high-confidence)			
TF43_BL_k141_98600	0.98	dsDNAphage	No	High-quality	100	High-quality draft genome	AAI-based (high-confidence)	*Myoviridae*		
TM23_26wk_k141_12581	1	dsDNAphage	No	Complete	100	High-quality draft genome	DTR (high-confidence)		*Firmicutes*	*Coprococcus catus*
TF02_12wk_k141_110135	1	ssDNA	No	High-quality	100	High-quality draft genome	AAI-based (medium-confidence)			
TM05_12wk_k141_128743	1	dsDNAphage	No	High-quality	100	High-quality draft genome	AAI-based (high-confidence)	*Microviridae*		
TF13_6wk_k141_64666	1	dsDNAphage	No	High-quality	100	High-quality draft genome	AAI-based (high-confidence)		*Bacteroidetes*	*Bacteroides stercoris*
TM06_BL_k141_189536	0.993	dsDNAphage	No	Complete	100	High-quality draft genome	DTR (high-confidence)	*Siphoviridae*		
TM10_26wk_k141_132283	0.98	dsDNAphage	No	Complete	100	High-quality draft genome	DTR (high-confidence)			
DM12_D6_k141_173055	0.993	dsDNAphage	No	Complete	100	High-quality draft genome	DTR (high-confidence)			
TF50_12wk_k141_110792	1	dsDNAphage	No	High-quality	100	High-quality draft genome	AAI-based (high-confidence)		*Bacteroidetes*	*Bacteroides vulgatus*
TF21_6WK_k141_77470	0.973	dsDNAphage	No	High-quality	97.55	High-quality draft genome	AAI-based (high-confidence)		*Firmicutes*	*Dorea longicatena*
TF22_26wk_k141_154527	1	dsDNAphage	No	Complete	100	High-quality draft genome	DTR (high-confidence)			
TM13_26wk_k141_102309	1	dsDNAphage	No	High-quality	100	High-quality draft genome	AAI-based (high-confidence)			
TM01_26wk_k141_70454	0.967	dsDNAphage	No	High-quality	100	High-quality draft genome	AAI-based (high-confidence)		*Actinobacteria*	*Bifidobacterium bifidum*
TF25_26wk_k141_80825	0.993	dsDNAphage	No	High-quality	98.51	High-quality draft genome	AAI-based (high-confidence)			
DF16_D3_k141_237558	0.98	dsDNAphage	Yes	High-quality	100	High-quality draft genome	AAI-based (high-confidence)			
TM05_BL_k141_95302	0.993	dsDNAphage	No	High-quality	100	High-quality draft genome	AAI-based (high-confidence)			
TM15_6wk_k141_38966	1	dsDNAphage	No	High-quality	100	High-quality draft genome	AAI-based (high-confidence)	*Myoviridae*		
TF05_26wk_k141_77383	1	dsDNAphage	Yes	High-quality	100	High-quality draft genome	AAI-based (high-confidence)		*Firmicutes*	*Ruminococcus bromii*
DF14_D2_k141_70346	1	dsDNAphage	No	High-quality	95.06	High-quality draft genome	AAI-based (high-confidence)	*Myoviridae*		
TM22_26wk_k141_88877	1	dsDNAphage	No	High-quality	93.05	High-quality draft genome	AAI-based (medium-confidence)			
DF17_D3_k141_68508	0.993	dsDNAphage	Yes	High-quality	100	High-quality draft genome	AAI-based (high-confidence)			
TF05_6wk_k141_116220	0.98	dsDNAphage	Yes	High-quality	94.48	High-quality draft genome	AAI-based (high-confidence)		*Firmicutes*	*Faecalibacterium prausnitzii*
TM13_6wk_k141_32692	0.993	dsDNAphage	No	Complete	100	High-quality draft genome	DTR (high-confidence)		*Firmicutes*	*Marvinbryantia formatexigens*
TF18_6wk_k141_191356	0.94	dsDNAphage	No	High-quality	100	High-quality draft genome	AAI-based (high-confidence)	*Microviridae*		
TF35_26wk_k141_215962	0.98	dsDNAphage	No	High-quality	100	High-quality draft genome	AAI-based (high-confidence)			
TF21_26wk_k141_220385	0.993	dsDNAphage	No	High-quality	100	High-quality draft genome	AAI-based (high-confidence)		*Firmicutes*	*Blautia obeum*
TF05_6wk_k141_28950	0.987	dsDNAphage	No	High-quality	100	High-quality draft genome	AAI-based (high-confidence)			
TF02_BL_k141_64800	1	dsDNAphage	Yes	High-quality	100	High-quality draft genome	AAI-based (high-confidence)			
DM12_D2_k141_104847	1	dsDNAphage	No	Complete	100	High-quality draft genome	DTR (high-confidence)			
TM18_BL_k141_23206	1	dsDNAphage	No	High-quality	91.45	High-quality draft genome	AAI-based (high-confidence)			
TF02_6wk_k141_156294	1	dsDNAphage	No	High-quality	100	High-quality draft genome	AAI-based (high-confidence)	*Myoviridae*		
TF42_12wk_k141_192419	1	dsDNAphage	No	High-quality	96.13	High-quality draft genome	AAI-based (high-confidence)	*Siphoviridae*	*Firmicutes*	*Catenibacterium mitsuokai*
TF35_12wk_k141_204050	1	dsDNAphage	No	High-quality	100	High-quality draft genome	AAI-based (high-confidence)			
DM07_D3_k141_105307	0.967	dsDNAphage	No	High-quality	94.06	High-quality draft genome	AAI-based (high-confidence)			
TF26_12wk_k141_127668	0.993	dsDNAphage	No	High-quality	100	High-quality draft genome	AAI-based (high-confidence)		*Bacteroidetes*	*Prevotella stercorea*
DF17_D8_k141_66990	0.973	dsDNAphage	Yes	High-quality	100	High-quality draft genome	AAI-based (high-confidence)			
TF14_6wk_k141_141212	0.993	dsDNAphage	No	High-quality	100	High-quality draft genome	AAI-based (high-confidence)			
TM15_6wk_k141_103289	0.98	dsDNAphage	No	High-quality	100	High-quality draft genome	AAI-based (high-confidence)		*Firmicutes*	*Ruminococcus lactaris*
TM09_BL_k141_92076	0.98	dsDNAphage	No	Complete	100	High-quality draft genome	DTR (high-confidence)			
TF16_26wk_k141_122745	0.993	dsDNAphage	No	High-quality	93.1	High-quality draft genome	AAI-based (medium-confidence)		*Bacteroidetes*	*Bacteroides uniformis*
TF33_6wk_k141_15405	1	dsDNAphage	No	High-quality	90.74	High-quality draft genome	AAI-based (high-confidence)	*Podoviridae*	*Bacteroidetes*	*Prevotella melaninogenica*
DM03_D6_k141_54817	0.993	dsDNAphage	No	High-quality	100	High-quality draft genome	AAI-based (high-confidence)			
TM30_12wk_k141_4819	1	dsDNAphage	No	High-quality	91.92	High-quality draft genome	AAI-based (high-confidence)	*Myoviridae*	*Firmicutes*	*Coprococcus catus*
TF20_BL_k141_175365	0.993	dsDNAphage	No	High-quality	100	High-quality draft genome	AAI-based (high-confidence)	*Siphoviridae*		
TF46_6wk_k141_194078	0.967	dsDNAphage	No	High-quality	98.42	High-quality draft genome	AAI-based (high-confidence)			
TM05_6wk_k141_3980	1	dsDNAphage	No	High-quality	100	High-quality draft genome	AAI-based (high-confidence)			
TF18_12wk_k141_4905	0.993	dsDNAphage	No	High-quality	100	High-quality draft genome	AAI-based (high-confidence)		*Firmicutes*	*Staphylococcus epidermidis*
TF31_26wk_k141_43880	1	dsDNAphage	No	High-quality	98.92	High-quality draft genome	AAI-based (high-confidence)	*Siphoviridae*		
TM15_6wk_k141_91005	1	dsDNAphage	No	High-quality	99.51	High-quality draft genome	AAI-based (high-confidence)			
TM30_BL_k141_133743	1	dsDNAphage	No	High-quality	100	High-quality draft genome	AAI-based (high-confidence)			
TF45_12wk_k141_91818	0.993	dsDNAphage	No	High-quality	100	High-quality draft genome	AAI-based (high-confidence)		*Firmicutes*	*Faecalibacterium prausnitzii*
TM29_BL_k141_193829	0.953	dsDNAphage	No	High-quality	100	High-quality draft genome	AAI-based (high-confidence)			
TF09_6WK_k141_100724	0.907	dsDNAphage	Yes	High-quality	100	High-quality draft genome	AAI-based (high-confidence)	*Microviridae*		
TM09_BL_k141_53663	1	dsDNAphage	No	Complete	100	High-quality draft genome	DTR (high-confidence)			
TF06_26wk_k141_94505	1	dsDNAphage	No	High-quality	95.26	High-quality draft genome	AAI-based (high-confidence)			
TF05_BL_k141_72410	0.993	dsDNAphage	No	High-quality	100	High-quality draft genome	AAI-based (high-confidence)			
TM23_26wk_k141_126527	0.98	dsDNAphage	No	High-quality	91.05	High-quality draft genome	AAI-based (high-confidence)			
TM07_12wk_k141_236409	1	dsDNAphage	No	High-quality	100	High-quality draft genome	AAI-based (high-confidence)			
TM08_26wk_k141_77775	1	dsDNAphage	No	High-quality	94.95	High-quality draft genome	AAI-based (high-confidence)		*Firmicutes*	*Dorea longicatena*
DF14_D2_k141_8237	0.98	dsDNAphage	Yes	High-quality	98.46	High-quality draft genome	AAI-based (high-confidence)			
TF03_6wk_k141_237804	0.993	dsDNAphage	No	High-quality	100	High-quality draft genome	AAI-based (high-confidence)		*Firmicutes*	*Staphylococcus epidermidis*
TM20_12wk_k141_203274	1	dsDNAphage	No	Complete	100	High-quality draft genome	DTR (high-confidence)		*Firmicutes*	*Tyzzerella nexilis*
TF47_BL_k141_15608	1	ssDNA	No	High-quality	100	High-quality draft genome	AAI-based (high-confidence)	*Microviridae*		
TF17_26wk_k141_35170	0.98	dsDNAphage	No	High-quality	100	High-quality draft genome	AAI-based (high-confidence)			
TF10_26wk_k141_51069	0.967	dsDNAphage	Yes	High-quality	91.76	High-quality draft genome	AAI-based (high-confidence)		*Firmicutes*	*Dorea longicatena*
TF01_26wk_k141_25705	1	dsDNAphage	No	High-quality	100	High-quality draft genome	AAI-based (high-confidence)	*Microviridae*		
TF09_6WK_k141_22712	0.993	dsDNAphage	No	High-quality	95.25	High-quality draft genome	AAI-based (high-confidence)			
TF32_BL_k141_54858	0.967	dsDNAphage	No	High-quality	95.34	High-quality draft genome	AAI-based (high-confidence)			
TF28_BL_k141_47332	1	dsDNAphage	No	Complete	100	High-quality draft genome	DTR (high-confidence)			
TM17_26wk_k141_55672	0.967	dsDNAphage	No	High-quality	100	High-quality draft genome	AAI-based (high-confidence)	*Microviridae*		
TF34_BL_k141_122199	0.993	dsDNAphage	No	High-quality	96.99	High-quality draft genome	AAI-based (high-confidence)			
TF27_6wk_k141_222733	1	dsDNAphage	No	Complete	100	High-quality draft genome	DTR (high-confidence)			
TF43_6wk_k141_24603	0.973	dsDNAphage	No	High-quality	100	High-quality draft genome	AAI-based (high-confidence)			
TM05_6wk_k141_54746	0.993	dsDNAphage	No	High-quality	100	High-quality draft genome	AAI-based (high-confidence)	*Myoviridae*		
TF41_26wk_k141_102042	0.993	dsDNAphage	No	High-quality	100	High-quality draft genome	AAI-based (high-confidence)		*Firmicutes*	*Faecalibacterium prausnitzii*
TF09_BL_k141_12896	1	dsDNAphage	No	High-quality	100	High-quality draft genome	AAI-based (high-confidence)			
TM36_6WK_k141_89349	0.967	dsDNAphage	No	High-quality	100	High-quality draft genome	AAI-based (high-confidence)		*Bacteroidetes*	*Prevotella sp. oral taxon 306*
TF32_6wk_k141_4581	1	dsDNAphage	No	High-quality	97.43	High-quality draft genome	AAI-based (high-confidence)			
TF50_6wk_k141_103474	1	dsDNAphage	No	High-quality	99.59	High-quality draft genome	AAI-based (high-confidence)			
TF13_6wk_k141_236604	1	dsDNAphage	No	High-quality	100	High-quality draft genome	AAI-based (high-confidence)			
TM09_BL_k141_8686	1	dsDNAphage	No	High-quality	93.79	High-quality draft genome	AAI-based (high-confidence)			
TF28_12wk_k141_51290	1	dsDNAphage	No	High-quality	98.75	High-quality draft genome	AAI-based (high-confidence)			
DM03_D4_k141_119	0.733	dsDNAphage	Yes	High-quality	91.51	High-quality draft genome	AAI-based (high-confidence)			
TF02_BL_k141_90577	1	dsDNAphage	No	High-quality	100	High-quality draft genome	AAI-based (high-confidence)			
TF04_BL_k141_59896	0.993	dsDNAphage	No	High-quality	100	High-quality draft genome	AAI-based (high-confidence)			
TM23_12wk_k141_14991	1	dsDNAphage	No	High-quality	100	High-quality draft genome	AAI-based (high-confidence)		*Actinobacteria*	*Bifidobacterium longum*
TF33_6wk_k141_176483	1	dsDNAphage	Yes	High-quality	100	High-quality draft genome	AAI-based (high-confidence)	*Podoviridae*	*Proteobacteria*	*Escherichia coli*
TM14_12wk_k141_77861	1	dsDNAphage	No	High-quality	100	High-quality draft genome	AAI-based (high-confidence)	*Podoviridae*		
DM12_D3_k141_149316	1	dsDNAphage	No	Complete	100	High-quality draft genome	DTR (high-confidence)	*Microviridae*		
TF10_12wk_k141_28949	0.993	ssDNA	No	High-quality	96.39	High-quality draft genome	AAI-based (high-confidence)	*Microviridae*		
TF22_26wk_k141_236072	0.993	dsDNAphage	No	Complete	100	High-quality draft genome	DTR (high-confidence)			
TM34_6wk_k141_6041	1	dsDNAphage	No	High-quality	100	High-quality draft genome	AAI-based (high-confidence)		*Actinobacteria*	*Bifidobacterium bifidum*
TM06_26wk_k141_146411	0.993	dsDNAphage	No	High-quality	95.92	High-quality draft genome	AAI-based (high-confidence)		*Firmicutes*	*Anaerostipes hadrus*
TF06_BL_k141_52433	1	dsDNAphage	No	High-quality	100	High-quality draft genome	AAI-based (high-confidence)			
DF14_D7_k141_25321	1	dsDNAphage	No	High-quality	99.21	High-quality draft genome	AAI-based (high-confidence)			
DM12_D3_k141_142433	0.993	dsDNAphage	No	High-quality	93.02	High-quality draft genome	AAI-based (high-confidence)			
TF17_12wk_k141_65625	1	dsDNAphage	No	High-quality	93.86	High-quality draft genome	AAI-based (high-confidence)		*Bacteroidetes*	*Bacteroides uniformis*
TM09_6wk_k141_125736	1	dsDNAphage	No	High-quality	100	High-quality draft genome	AAI-based (high-confidence)		*Firmicutes*	*Roseburia intestinalis*
TF25_6WK_k141_506	1	dsDNAphage	Yes	Complete	100	High-quality draft genome	Provirus (high-confidence)	*Siphoviridae*	*Firmicutes*	*Anaerobutyricum hallii*
DF14_D8_k141_13616	1	dsDNAphage	No	High-quality	100	High-quality draft genome	AAI-based (high-confidence)	*Myoviridae*		
DF12_D4_k141_18488	0.993	dsDNAphage	Yes	High-quality	100	High-quality draft genome	AAI-based (high-confidence)			
TM04_12wk_k141_78696	0.993	dsDNAphage	No	Complete	100	High-quality draft genome	ITR (high-confidence)		*Firmicutes*	*Ruminococcus bromii*

UViG	VirSorter2 score	Predicted genome type	Provirus	Completeness quality	Completeness (%)	Assembly quality	Completeness approach	Family	Host phylum	Host species
TF02_6wk_k141_131917	0.927	ssDNA	No	High-quality	100	High-quality draft genome	AAI-based (high-confidence)			
TF22_BL_k141_162553	1	dsDNAphage	No	High-quality	100	High-quality draft genome	AAI-based (high-confidence)	*Crass_like*		
TF34_6wk_k141_201745	1	dsDNAphage	No	High-quality	100	High-quality draft genome	AAI-based (high-confidence)			
TF15_BL_k141_14479	1	dsDNAphage	No	High-quality	93.57	High-quality draft genome	AAI-based (high-confidence)	*Myoviridae*		
TM02_BL_k141_49248	0.993	dsDNAphage	No	High-quality	100	High-quality draft genome	AAI-based (high-confidence)			
TM36_12wk_k141_83567	1	dsDNAphage	No	High-quality	100	High-quality draft genome	AAI-based (high-confidence)		*Firmicutes*	*Coprococcus comes*
TF23_6wk_k141_184596	0.973	dsDNAphage	No	Complete	100	High-quality draft genome	DTR (high-confidence)		*Firmicutes*	*Anaerobutyricum hallii*
TM33_12wk_k141_38344	1	dsDNAphage	No	High-quality	92.5	High-quality draft genome	AAI-based (medium-confidence)		*Firmicutes*	*Megamonas hypermegale*
DM03_D6_k141_32168	0.993	dsDNAphage	No	High-quality	100	High-quality draft genome	AAI-based (high-confidence)			
DF17a_D2_k141_377	0.62	dsDNAphage	No	High-quality	100	High-quality draft genome	HMM-based (lower-bound)			
TF04_BL_k141_10907	0.973	dsDNAphage	No	High-quality	100	High-quality draft genome	AAI-based (high-confidence)			
DF17a_D2_k141_46944	1	dsDNAphage	Yes	High-quality	100	High-quality draft genome	AAI-based (high-confidence)			
TF13_6wk_k141_72315	0.987	dsDNAphage	Yes	High-quality	93.59	High-quality draft genome	AAI-based (high-confidence)		*Firmicutes*	*Dorea longicatena*
TM11_BL_k141_72848	0.987	ssDNA	No	High-quality	100	High-quality draft genome	HMM-based (lower-bound)			
DM12_D3_k141_11631	0.987	dsDNAphage	Yes	High-quality	93.39	High-quality draft genome	AAI-based (high-confidence)			
TF12_6wk_k141_152538	0.68	dsDNAphage	No	Complete	100	High-quality draft genome	DTR (high-confidence)			
TM20_BL_k141_82676	0.987	dsDNAphage	No	High-quality	90.79	High-quality draft genome	AAI-based (high-confidence)			
TF12_26wk_k141_126462	1	dsDNAphage	No	High-quality	99.2	High-quality draft genome	AAI-based (high-confidence)		*Firmicutes*	*Roseburia inulinivorans*
TF30_BL_k141_71792	1	dsDNAphage	No	Complete	100	High-quality draft genome	DTR (high-confidence)	*Podoviridae*		
TM34_12wk_k141_95669	1	dsDNAphage	No	High-quality	100	High-quality draft genome	AAI-based (high-confidence)		*Firmicutes*	*Roseburia intestinalis*
TF42_26wk_k141_18029[illegible]	1	dsDNAphage	No	High-quality	100	High-quality draft genome	AAI-based (high-confidence)		*Firmicutes*	*Coprococcus catus*
TF36_26wk_k141_40510	1	dsDNAphage	No	Complete	100	High-quality draft genome	DTR (high-confidence)		*Bacteroidetes*	*Bacteroides clarus*
TM05_26wk_k141_23035[illegible]	1	dsDNAphage	No	High-quality	100	High-quality draft genome	AAI-based (medium-confidence)			
TM20_BL_k141_152324	1	dsDNAphage	No	High-quality	100	High-quality draft genome	AAI-based (high-confidence)			
TM03_BL_k141_3571	0.98	dsDNAphage	No	High-quality	100	High-quality draft genome	AAI-based (high-confidence)			
TF47_26wk_k141_224084	1	dsDNAphage	No	Complete	100	High-quality draft genome	DTR (high-confidence)			
TM07_6wk_k141_223424	1	dsDNAphage	No	High-quality	98.42	High-quality draft genome	AAI-based (high-confidence)			
DM08_D2_k141_157809	0.987	dsDNAphage	No	High-quality	100	High-quality draft genome	AAI-based (high-confidence)			
TF14_6wk_k141_211817	0.993	dsDNAphage	No	High-quality	95.87	High-quality draft genome	AAI-based (high-confidence)			
TF22_BL_k141_110462	1	dsDNAphage	No	High-quality	91.12	High-quality draft genome	AAI-based (medium-confidence)			
TM30_6wk_k141_6917	1	dsDNAphage	No	High-quality	100	High-quality draft genome	AAI-based (high-confidence)		*Firmicutes*	*Faecalibacterium prausnitzii*
TF24_26wk_k141_101372	0.953	dsDNAphage	No	High-quality	100	High-quality draft genome	AAI-based (high-confidence)		*Actinobacteria*	*Bifidobacterium bifidum*
DF12_D6_k141_66449	0.993	dsDNAphage	Yes	High-quality	100	High-quality draft genome	AAI-based (medium-confidence)			
TM14_6wk_k141_5173	1	dsDNAphage	No	High-quality	100	High-quality draft genome	AAI-based (high-confidence)		*Firmicutes*	*Blautia obeum*
TM28_BL_k141_237776	1	dsDNAphage	No	High-quality	99.36	High-quality draft genome	AAI-based (high-confidence)			
TF11_26wk_k141_92863	1	dsDNAphage	No	High-quality	100	High-quality draft genome	AAI-based (high-confidence)		*Firmicutes*	*Tyzzerella nexilis*
TM21_BL_k141_11158	0.987	dsDNAphage	No	Complete	100	High-quality draft genome	DTR (high-confidence)			
TM06_26wk_k141_17849[illegible]	1	dsDNAphage	No	Complete	100	High-quality draft genome	DTR (high-confidence)		*Firmicutes*	*Faecalibacterium prausnitzii*
TM10_BL_k141_57373	0.993	dsDNAphage	No	Complete	100	High-quality draft genome	DTR (high-confidence)			
TF27_26wk_k141_176972	1	dsDNAphage	No	High-quality	93.18	High-quality draft genome	AAI-based (high-confidence)		*Firmicutes*	*Faecalibacterium prausnitzii*
TF12_26wk_k141_159112	0.98	dsDNAphage	No	Complete	100	High-quality draft genome	DTR (high-confidence)			
TM09_6wk_k141_14876	1	dsDNAphage	No	High-quality	100	High-quality draft genome	AAI-based (high-confidence)		*Firmicutes*	*Anaerostipes hadrus*
TF03_12wk_k141_41672	0.993	dsDNAphage	No	High-quality	96.73	High-quality draft genome	AAI-based (medium-confidence)			
TF18_12wk_k141_37938	0.993	dsDNAphage	No	Complete	100	High-quality draft genome	DTR (high-confidence)		*Firmicutes*	*Butyrivibrio crossotus*
TF29_6WK_k141_90159	1	dsDNAphage	No	High-quality	94.91	High-quality draft genome	AAI-based (high-confidence)		*Firmicutes*	*Dorea longicatena*
DF16_D6_k141_276724	0.993	dsDNAphage	No	High-quality	100	High-quality draft genome	AAI-based (high-confidence)			
TM15_BL_k141_7777	1	dsDNAphage	No	High-quality	100	High-quality draft genome	AAI-based (high-confidence)			
DM12_D2_k141_19310	1	dsDNAphage	Yes	High-quality	100	High-quality draft genome	AAI-based (medium-confidence)			
TF17_26wk_k141_71629	1	dsDNAphage	No	High-quality	100	High-quality draft genome	AAI-based (high-confidence)		*Firmicutes*	*Tyzzerella nexilis*
TF22_12wk_k141_3927	0.993	dsDNAphage	Yes	High-quality	100	High-quality draft genome	AAI-based (high-confidence)			
DF14_D7_k141_38324	1	dsDNAphage	No	Complete	100	High-quality draft genome	DTR (high-confidence)			
TF24_26wk_k141_17202	1	dsDNAphage	Yes	High-quality	93.37	High-quality draft genome	AAI-based (high-confidence)			
TM03_26wk_k141_18483[illegible]	1	dsDNAphage	No	Complete	100	High-quality draft genome	DTR (high-confidence)			
TF23_6wk_k141_34094	0.987	dsDNAphage	No	High-quality	100	High-quality draft genome	AAI-based (high-confidence)			
TF40_6wk_k141_54507	0.993	dsDNAphage	No	High-quality	97.06	High-quality draft genome	AAI-based (high-confidence)	*Siphoviridae*		
DM08_D5_k141_187999	1	dsDNAphage	No	Complete	100	High-quality draft genome	DTR (medium-confidence)	*Myoviridae*		
TF48_6wk_k141_135683	0.973	dsDNAphage	No	High-quality	100	High-quality draft genome	AAI-based (high-confidence)			
TF22_6wk_k141_47159	0.993	dsDNAphage	No	High-quality	90.74	High-quality draft genome	AAI-based (high-confidence)	*Myoviridae*		
TF29_12wk_k141_172614	0.973	dsDNAphage	No	High-quality	97.52	High-quality draft genome	AAI-based (medium-confidence)			
TF31_12wk_k141_68010	0.98	dsDNAphage	No	High-quality	100	High-quality draft genome	AAI-based (medium-confidence)		*Firmicutes*	*Hungatella hathewayi*
TM10_BL_k141_50059	0.973	dsDNAphage	No	Complete	100	High-quality draft genome	DTR (high-confidence)			
TF21_12wk_k141_84989	0.993	dsDNAphage	No	High-quality	100	High-quality draft genome	AAI-based (high-confidence)		*Firmicutes*	*Faecalibacterium prausnitzii*
DF17a_D2_k141_22436	0.987	dsDNAphage	No	High-quality	96.36	High-quality draft genome	AAI-based (medium-confidence)			
TF44_12wk_k141_10745	0.993	dsDNAphage	No	High-quality	96.73	High-quality draft genome	AAI-based (high-confidence)		*Firmicutes*	*Dorea longicatena*
DM12_D4_k141_113302	0.98	dsDNAphage	No	Complete	100	High-quality draft genome	DTR (high-confidence)			
TM30_12wk_k141_76583	1	dsDNAphage	No	High-quality	94.02	High-quality draft genome	AAI-based (high-confidence)			
TF42_26wk_k141_182494	0.98	dsDNAphage	No	High-quality	95.81	High-quality draft genome	AAI-based (high-confidence)			
TF49_12wk_k141_211932	0.993	dsDNAphage	No	Complete	100	High-quality draft genome	DTR (high-confidence)	*Crass_like*	*Firmicutes*	*Staphylococcus aureus*
TM30_26wk_k141_25266[illegible]	1	dsDNAphage	No	Complete	100	High-quality draft genome	DTR (high-confidence)			
TF30_12wk_k141_176618	0.967	dsDNAphage	No	High-quality	98.43	High-quality draft genome	AAI-based (high-confidence)		*Firmicutes*	*Marvinbryantia formatexigens*
TF45_12wk_k141_148419	0.993	dsDNAphage	No	High-quality	100	High-quality draft genome	AAI-based (high-confidence)		*Firmicutes*	*Blautia obeum*
TF28_6wk_k141_23703	1	dsDNAphage	No	High-quality	96.97	High-quality draft genome	AAI-based (high-confidence)		*Actinobacteria*	*Bifidobacterium longum*
TF44_6wk_k141_124374	0.98	dsDNAphage	No	High-quality	99.99	High-quality draft genome	AAI-based (high-confidence)	*Microviridae*		
TM14_BL_k141_174143	0.993	dsDNAphage	No	Complete	100	High-quality draft genome	DTR (high-confidence)	*Crass_like*		
TF08_BL_k141_6144	0.993	dsDNAphage	No	High-quality	97.51	High-quality draft genome	AAI-based (high-confidence)		*Firmicutes*	*Ruminococcus bromii*
TM08_6wk_k141_167840	0.993	dsDNAphage	No	High-quality	100	High-quality draft genome	AAI-based (high-confidence)	*Myoviridae*		
TM13_26wk_k141_10093[illegible]	0.993	dsDNAphage	No	High-quality	96.83	High-quality draft genome	AAI-based (high-confidence)	*Crass_like*	*Firmicutes*	*Clostridium sporogenes*
TM04_BL_k141_127596	1	dsDNAphage	No	High-quality	100	High-quality draft genome	AAI-based (high-confidence)			
DF16_D6_k141_363298	1	dsDNAphage	No	Complete	100	High-quality draft genome	DTR (high-confidence)			
TM24_26wk_k141_11734[illegible]	0.933	ssDNA	No	High-quality	100	High-quality draft genome	HMM-based (lower-bound)			
TF17_26wk_k141_211864	0.993	dsDNAphage	No	High-quality	97.43	High-quality draft genome	AAI-based (high-confidence)			
TM39_12wk_k141_62233	0.993	dsDNAphage	No	High-quality	100	High-quality draft genome	AAI-based (high-confidence)		*Firmicutes*	*Faecalibacterium prausnitzii*
DM03_D6_k141_61947	0.993	dsDNAphage	Yes	High-quality	100	High-quality draft genome	AAI-based (high-confidence)			
TM17_BL_k141_83176	0.973	dsDNAphage	No	High-quality	95.29	High-quality draft genome	AAI-based (high-confidence)			
TM17_12wk_k141_72302	1	dsDNAphage	No	Complete	100	High-quality draft genome	DTR (high-confidence)		*Firmicutes*	*Megamonas funiformis*
TM27_26wk_k141_94343	1	dsDNAphage	No	Complete	100	High-quality draft genome	DTR (high-confidence)	*Crass_like*	*Bacteroidetes*	*Prevotella melaninogenica*
DM07_D4_k141_2815	0.993	dsDNAphage	No	High-quality	100	High-quality draft genome	AAI-based (high-confidence)			
TF37_12wk_k141_146475	0.98	dsDNAphage	Yes	High-quality	91.54	High-quality draft genome	AAI-based (high-confidence)	*Siphoviridae*	*Firmicutes*	*Faecalibacterium prausnitzii*
TF35_BL_k141_33687	0.993	dsDNAphage	No	High-quality	100	High-quality draft genome	AAI-based (high-confidence)			
DM12_D2_k141_7271	1	dsDNAphage	No	High-quality	91.46	High-quality draft genome	AAI-based (high-confidence)			
TM05_BL_k141_80740	0.993	dsDNAphage	No	High-quality	100	High-quality draft genome	AAI-based (high-confidence)	*Microviridae*		
TM15_BL_k141_71595	1	dsDNAphage	No	Complete	100	High-quality draft genome	DTR (high-confidence)			
TF26_6wk_k141_50121	0.653	dsDNAphage	No	High-quality	100	High-quality draft genome	AAI-based (high-confidence)			
DM07_D3_k141_15303	0.987	dsDNAphage	No	High-quality	100	High-quality draft genome	AAI-based (high-confidence)			
TF46_12wk_k141_4985	0.98	dsDNAphage	No	High-quality	100	High-quality draft genome	AAI-based (high-confidence)			
TF40_BL_k141_109655	1	dsDNAphage	No	Complete	100	High-quality draft genome	DTR (high-confidence)			
TM12_BL_k141_5680	1	dsDNAphage	No	High-quality	100	High-quality draft genome	AAI-based (medium-confidence)			
TF10_6wk_k141_18539	0.98	dsDNAphage	Yes	High-quality	96.6	High-quality draft genome	AAI-based (medium-confidence)	*Myoviridae*	*Firmicutes*	*Roseburia inulinivorans*
TF26_26wk_k141_186896	1	dsDNAphage	No	Complete	100	High-quality draft genome	DTR (high-confidence)			
TF32_6wk_k141_34497	0.853	ssDNA	No	High-quality	100	High-quality draft genome	HMM-based (lower-bound)			
TF08_BL_k141_80911	0.993	dsDNAphage	No	High-quality	95.67	High-quality draft genome	AAI-based (high-confidence)	*Siphoviridae*		
TF24_26wk_k141_185490	0.98	dsDNAphage	No	Complete	100	High-quality draft genome	DTR (high-confidence)	*Circoviridae*		
TF47_26wk_k141_180730	0.987	dsDNAphage	No	Complete	100	High-quality draft genome	DTR (high-confidence)			

UViG	VirSorter2 score	Predicted genome type	Provirus	Completeness quality	Completeness (%)	Assembly quality	Completeness approach	Family	Host phylum	Host species
TF47_12wk_k141_214962	1	dsDNAphage	No	High-quality	98.85	High-quality draft genome	AAI-based (high-confidence)			
DF14_D7_k141_177825	1	dsDNAphage	No	Complete	100	High-quality draft genome	DTR (high-confidence)			
DF17_D8_k141_20193	0.98	dsDNAphage	Yes	High-quality	90.38	High-quality draft genome	AAI-based (high-confidence)			
TF24_26wk_k141_52157	0.993	dsDNAphage	No	Complete	100	High-quality draft genome	DTR (high-confidence)		*Actinobacteria*	*Bifidobacterium adolescentis*
TF50_12wk_k141_95797	0.98	dsDNAphage	No	High-quality	97.73	High-quality draft genome	AAI-based (high-confidence)		*Firmicutes*	*Coprococcus catus*
TF25_6WK_k141_67510	0.98	dsDNAphage	No	Complete	100	High-quality draft genome	DTR (high-confidence)		*Firmicutes*	*Flavonifractor plautii*
TM08_26wk_k141_2118	0.993	dsDNAphage	No	Complete	100	High-quality draft genome	DTR (high-confidence)			
TM12_6WK_k141_101783	0.993	dsDNAphage	No	High-quality	100	High-quality draft genome	AAI-based (high-confidence)	*Myoviridae*	*Firmicutes*	*Roseburia inulinivorans*
TF23_12wk_k141_93478	0.96	dsDNAphage	No	High-quality	100	High-quality draft genome	HMM-based (lower-bound)			
TF09_BL_k141_31304	0.993	dsDNAphage	No	High-quality	100	High-quality draft genome	AAI-based (high-confidence)			
TM02_12wk_k141_121593	1	dsDNAphage	No	High-quality	100	High-quality draft genome	AAI-based (high-confidence)		*Bacteroidetes*	*Prevotella disiens*
TM06_26wk_k141_63973	1	dsDNAphage	No	High-quality	98.42	High-quality draft genome	AAI-based (high-confidence)		*Firmicutes*	*Anaerobutyricum hallii*
TF13_12wk_k141_118769	0.993	dsDNAphage	No	High-quality	100	High-quality draft genome	AAI-based (high-confidence)	*Podoviridae*		
TM13_BL_k141_5783	1	dsDNAphage	No	High-quality	99.5	High-quality draft genome	AAI-based (high-confidence)			
TF28_12wk_k141_53195	0.987	dsDNAphage	No	High-quality	100	High-quality draft genome	AAI-based (medium-confidence)		*Actinobacteria*	*Bifidobacterium pseudocatenulatum*
TF03_26wk_k141_13452	1	dsDNAphage	No	High-quality	100	High-quality draft genome	AAI-based (high-confidence)			
TF31_12wk_k141_95311	1	dsDNAphage	No	High-quality	96.88	High-quality draft genome	AAI-based (high-confidence)		*Actinobacteria*	*Bifidobacterium adolescentis*
TF38_12wk_k141_242113	0.66	dsDNAphage	No	Complete	100	High-quality draft genome	DTR (high-confidence)			
TF20_12wk_k141_136695	0.973	dsDNAphage	No	High-quality	94.7	High-quality draft genome	AAI-based (high-confidence)			
TM06_26wk_k141_179906	0.993	dsDNAphage	No	High-quality	100	High-quality draft genome	AAI-based (high-confidence)		*Firmicutes*	*Anaerostipes hadrus*
TF25_BL_k141_143955	1	dsDNAphage	No	Complete	100	High-quality draft genome	DTR (high-confidence)			
TM36_12wk_k141_92020	1	dsDNAphage	No	Complete	100	High-quality draft genome	DTR (high-confidence)			
TF45_26wk_k141_140194	1	dsDNAphage	No	High-quality	100	High-quality draft genome	AAI-based (high-confidence)			
TF03_6wk_k141_218677	0.667	ssDNA	No	High-quality	100	High-quality draft genome	HMM-based (lower-bound)	*Circoviridae*		
TF39_6wk_k141_16376	1	dsDNAphage	No	Complete	100	High-quality draft genome	DTR (high-confidence)		*Firmicutes*	*Roseburia intestinalis*
DF14_D8_k141_45534	0.993	dsDNAphage	No	High-quality	93.15	High-quality draft genome	AAI-based (high-confidence)			
DF16_D3_k141_273741	0.733	dsDNAphage	No	High-quality	100	High-quality draft genome	HMM-based (lower-bound)			
TF48_26wk_k141_2715	1	dsDNAphage	No	High-quality	100	High-quality draft genome	AAI-based (high-confidence)		*Actinobacteria*	*Bifidobacterium longum*
DM12_D2_k141_33379	1	dsDNAphage	No	High-quality	94.38	High-quality draft genome	AAI-based (high-confidence)			
TF48_26wk_k141_63513	0.993	dsDNAphage	No	High-quality	100	High-quality draft genome	AAI-based (high-confidence)			
DF17a_D2_k141_130185	0.973	dsDNAphage	No	High-quality	100	High-quality draft genome	AAI-based (high-confidence)	*Microviridae*		
TF47_26wk_k141_21056	1	dsDNAphage	No	High-quality	99.79	High-quality draft genome	AAI-based (high-confidence)		*Firmicutes*	*Ruminococcus bromii*
TM30_12wk_k141_173999	1	ssDNA	No	High-quality	100	High-quality draft genome	AAI-based (high-confidence)	*Microviridae*		
TF19_12wk_k141_38265	0.993	dsDNAphage	No	High-quality	100	High-quality draft genome	AAI-based (high-confidence)			
DF12_D7_k141_72295	1	dsDNAphage	No	High-quality	100	High-quality draft genome	AAI-based (high-confidence)	*Siphoviridae*		
DM03_D1_k141_111992	0.993	dsDNAphage	Yes	High-quality	98.57	High-quality draft genome	AAI-based (high-confidence)	*Siphoviridae*	*Firmicutes*	*Anaerostipes hadrus*
DM12_D4_k141_118158	0.98	dsDNAphage	No	High-quality	100	High-quality draft genome	AAI-based (high-confidence)	*Myoviridae*		
TF20_BL_k141_203010	0.987	dsDNAphage	No	High-quality	97.66	High-quality draft genome	AAI-based (high-confidence)			
TF40_BL_k141_83210	0.993	dsDNAphage	No	High-quality	100	High-quality draft genome	AAI-based (high-confidence)		*Firmicutes*	*Roseburia intestinalis*
TM24_6wk_k141_81188	0.967	dsDNAphage	No	High-quality	100	High-quality draft genome	AAI-based (medium-confidence)		*Firmicutes*	*Megamonas funiformis*
TF06_12wk_k141_37859	1	dsDNAphage	No	High-quality	97.93	High-quality draft genome	AAI-based (high-confidence)			
DF16_D5_k141_306272	1	dsDNAphage	No	Complete	100	High-quality draft genome	DTR (high-confidence)			
TF26_26wk_k141_84687	1	dsDNAphage	No	High-quality	100	High-quality draft genome	AAI-based (high-confidence)			
TF20_6WK_k141_144767	1	dsDNAphage	No	High-quality	98.34	High-quality draft genome	AAI-based (medium-confidence)	*Podoviridae*		
TF37_6wk_k141_46257	0.96	ssDNA	No	High-quality	100	High-quality draft genome	HMM-based (lower-bound)			
TF11_26wk_k141_46942	0.987	dsDNAphage	No	Complete	100	High-quality draft genome	DTR (high-confidence)			
TF10_12wk_k141_132867	0.987	dsDNAphage	No	High-quality	92.1	High-quality draft genome	AAI-based (medium-confidence)			
TF47_26wk_k141_213576	0.993	dsDNAphage	No	Complete	100	High-quality draft genome	DTR (high-confidence)			
TM04_BL_k141_165097	0.993	dsDNAphage	No	High-quality	100	High-quality draft genome	AAI-based (high-confidence)			
TF51_6wk_k141_3137	1	dsDNAphage	No	High-quality	100	High-quality draft genome	AAI-based (high-confidence)	*Myoviridae*		
TM13_6wk_k141_142067	0.987	dsDNAphage	No	Complete	100	High-quality draft genome	DTR (high-confidence)		*Firmicutes*	*Coprococcus comes*
TM28_BL_k141_98145	0.98	dsDNAphage	No	High-quality	100	High-quality draft genome	AAI-based (high-confidence)			
TF31_12wk_k141_5018	1	dsDNAphage	No	Complete	100	High-quality draft genome	DTR (high-confidence)		*Bacteroidetes*	*Bacteroides uniformis*
TF22_26wk_k141_92929	1	dsDNAphage	No	High-quality	94.66	High-quality draft genome	AAI-based (high-confidence)	*Myoviridae*		
TF30_26wk_k141_96576	1	dsDNAphage	No	High-quality	100	High-quality draft genome	AAI-based (high-confidence)		*Firmicutes*	*Coprococcus comes*
TF44_BL_k141_38987	1	dsDNAphage	No	High-quality	100	High-quality draft genome	AAI-based (medium-confidence)			
TF42_6wk_k141_38866	1	dsDNAphage	No	High-quality	100	High-quality draft genome	AAI-based (high-confidence)			
TF41_BL_k141_47565	0.973	dsDNAphage	No	High-quality	100	High-quality draft genome	AAI-based (medium-confidence)			
TF48_BL_k141_94050	0.987	dsDNAphage	No	High-quality	90.81	High-quality draft genome	AAI-based (high-confidence)			
TF15_BL_k141_117091	0.98	dsDNAphage	No	Complete	100	High-quality draft genome	DTR (high-confidence)			
TM16_12wk_k141_28174	0.96	dsDNAphage	No	High-quality	100	High-quality draft genome	AAI-based (medium-confidence)		*Euryarchaeota*	*Methanobrevibacter smithii*
TM08_26wk_k141_191922	0.967	dsDNAphage	No	High-quality	100	High-quality draft genome	AAI-based (medium-confidence)		*Firmicutes*	*Faecalibacterium prausnitzii*
TM20_BL_k141_158691	1	dsDNAphage	No	High-quality	96.27	High-quality draft genome	AAI-based (high-confidence)	*Crass_like*		
TF37_6wk_k141_78318	1	dsDNAphage	No	Complete	100	High-quality draft genome	DTR (high-confidence)	*Crass_like*	*Firmicutes*	*Staphylococcus aureus*
TF07_26wk_k141_85994	1	dsDNAphage	No	High-quality	91.09	High-quality draft genome	AAI-based (high-confidence)		*Bacteroidetes*	*Prevotella copri*
TM34_6wk_k141_98401	1	dsDNAphage	No	High-quality	100	High-quality draft genome	AAI-based (high-confidence)		*Firmicutes*	*Anaerobutyricum hallii*
TM08_12wk_k141_51210	0.987	dsDNAphage	No	High-quality	92.13	High-quality draft genome	AAI-based (high-confidence)		*Firmicutes*	*Ruminococcus bromii*
TM23_BL_k141_48430	1	dsDNAphage	No	High-quality	97.2	High-quality draft genome	AAI-based (high-confidence)			
DF17_D6_k141_139028	0.993	dsDNAphage	Yes	High-quality	100	High-quality draft genome	AAI-based (high-confidence)			
TM32_12wk_k141_40057	1	dsDNAphage	No	Complete	100	High-quality draft genome	DTR (high-confidence)	*Crass_like*	*Bacteroidetes*	*Parabacteroides johnsonii*
DM07_D4_k141_214202	1	dsDNAphage	No	High-quality	100	High-quality draft genome	AAI-based (high-confidence)			
TF40_6wk_k141_109570	0.993	dsDNAphage	No	High-quality	100	High-quality draft genome	AAI-based (high-confidence)		*Bacteroidetes*	*Bacteroides caccae*
DF17_D6_k141_14665	1	dsDNAphage	Yes	High-quality	100	High-quality draft genome	AAI-based (medium-confidence)	*Siphoviridae*		
DM12_D2_k141_96457	0.793	dsDNAphage	No	High-quality	100	High-quality draft genome	AAI-based (medium-confidence)			
TF22_BL_k141_119577	1	dsDNAphage	No	High-quality	90.93	High-quality draft genome	AAI-based (medium-confidence)			
TF21_6WK_k141_22343	1	dsDNAphage	No	High-quality	90.76	High-quality draft genome	AAI-based (high-confidence)		*Actinobacteria*	*Bifidobacterium adolescentis*
TF17_26wk_k141_7585	0.993	dsDNAphage	No	High-quality	100	High-quality draft genome	AAI-based (high-confidence)		*Firmicutes*	*Marvinbryantia formatexigens*
TF25_BL_k141_15551	1	dsDNAphage	No	High-quality	98.31	High-quality draft genome	AAI-based (high-confidence)			
DM03_D1_k141_127167	0.98	dsDNAphage	Yes	High-quality	92.37	High-quality draft genome	AAI-based (high-confidence)			
TF37_6wk_k141_72445	0.98	dsDNAphage	No	High-quality	100	High-quality draft genome	AAI-based (high-confidence)			
TM18_BL_k141_21724	0.987	dsDNAphage	No	High-quality	100	High-quality draft genome	AAI-based (medium-confidence)			
TF06_26wk_k141_106312	1	dsDNAphage	No	High-quality	90.32	High-quality draft genome	AAI-based (medium-confidence)			
TF07_12wk_k141_181182	0.987	dsDNAphage	Yes	High-quality	100	High-quality draft genome	AAI-based (high-confidence)		*Bacteroidetes*	*Bacteroides stercoris*
TF20_26wk_k141_84392	1	dsDNAphage	No	Complete	100	High-quality draft genome	ITR (high-confidence)	*Podoviridae*	*Actinobacteria*	*Bifidobacterium pseudocatenulatum*
TM15_BL_k141_157103	1	dsDNAphage	No	High-quality	100	High-quality draft genome	AAI-based (high-confidence)			
TM27_26wk_k141_81847	0.993	dsDNAphage	No	High-quality	100	High-quality draft genome	AAI-based (high-confidence)			
TF25_BL_k141_117557	0.98	dsDNAphage	No	High-quality	92.29	High-quality draft genome	AAI-based (high-confidence)			
TM34_26wk_k141_176244	0.993	dsDNAphage	No	High-quality	93.29	High-quality draft genome	AAI-based (high-confidence)			
DM05_D1_k141_121870	0.96	dsDNAphage	Yes	High-quality	90.44	High-quality draft genome	AAI-based (high-confidence)	*Siphoviridae*		
TF08_BL_k141_10721	0.927	dsDNAphage	No	High-quality	100	High-quality draft genome	AAI-based (high-confidence)			
TM06_6wk_k141_83380	0.993	dsDNAphage	No	High-quality	99.69	High-quality draft genome	AAI-based (high-confidence)		*Firmicutes*	*Anaerostipes hadrus*
DF17_D4_k141_101905	0.993	dsDNAphage	No	High-quality	98.49	High-quality draft genome	AAI-based (high-confidence)	*Myoviridae*		
TF25_6WK_k141_132570	1	dsDNAphage	No	High-quality	100	High-quality draft genome	AAI-based (medium-confidence)			
TM24_6wk_k141_127016	0.993	dsDNAphage	No	High-quality	100	High-quality draft genome	AAI-based (high-confidence)			
TM09_6wk_k141_55346	1	dsDNAphage	No	High-quality	91.44	High-quality draft genome	AAI-based (high-confidence)		*Firmicutes*	*Dorea longicatena*
TF10_6wk_k141_92630	1	dsDNAphage	No	High-quality	100	High-quality draft genome	AAI-based (high-confidence)		*Bacteroidetes*	*Prevotella copri*
TF22_BL_k141_42604	0.993	dsDNAphage	No	High-quality	93.21	High-quality draft genome	AAI-based (high-confidence)			
TF31_12wk_k141_26422	0.993	dsDNAphage	No	High-quality	100	High-quality draft genome	AAI-based (high-confidence)	*Siphoviridae*	*Bacteroidetes*	*Parabacteroides johnsonii*
DF14_D5_k141_15624	1	dsDNAphage	No	High-quality	100	High-quality draft genome	AAI-based (high-confidence)	*Siphoviridae*		
TF46_6wk_k141_14621	0.993	dsDNAphage	No	Complete	100	High-quality draft genome	DTR (high-confidence)		*Firmicutes*	*Blautia obeum*
TM32_12wk_k141_21945	1	dsDNAphage	No	Complete	100	High-quality draft genome	DTR (high-confidence)		*Firmicutes*	*Staphylococcus epidermidis*
DF17_D4_k141_121942	0.987	dsDNAphage	No	High-quality	100	High-quality draft genome	AAI-based (high-confidence)			
TF35_26wk_k141_13666	1	dsDNAphage	No	High-quality	100	High-quality draft genome	AAI-based (high-confidence)	*Siphoviridae*	*Firmicutes*	*Ruminococcus bromii*
TF12_6wk_k141_162359	0.993	dsDNAphage	No	High-quality	100	High-quality draft genome	AAI-based (high-confidence)		*Firmicutes*	*Ruminococcus bromii*
TF18_6wk_k141_180922	1	dsDNAphage	Yes	High-quality	98.54	High-quality draft genome	AAI-based (high-confidence)		*Firmicutes*	*Blautia obeum*

UViG	VirSorter2 score	Predicted genome type	Provirus	Completeness quality	Completeness (%)	Assembly quality	Completeness approach	Family	Host phylum	Host species
TF02_12wk_k141_49281	1	dsDNAphage	Yes	High-quality	94.04	High-quality draft genome	AAI-based (high-confidence)			
TM30_12wk_k141_82377	1	dsDNAphage	No	High-quality	95.38	High-quality draft genome	AAI-based (high-confidence)		*Bacteroidetes*	*Bacteroides salyersiae*
TF19_26wk_k141_54529	1	dsDNAphage	Yes	High-quality	94.67	High-quality draft genome	AAI-based (high-confidence)		*Firmicutes*	*Faecalibacterium prausnitzii*
TM02_26wk_k141_43732	0.993	dsDNAphage	No	High-quality	92.98	High-quality draft genome	AAI-based (medium-confidence)		*Firmicutes*	*Roseburia inulinivorans*
TF37_12wk_k141_4021	1	dsDNAphage	No	High-quality	100	High-quality draft genome	AAI-based (high-confidence)		*Bacteroidetes*	*Bacteroides ovatus*
TF43_BL_k141_156113	0.993	dsDNAphage	No	High-quality	99.81	High-quality draft genome	AAI-based (high-confidence)		*Bacteroidetes*	*Bacteroides dorei*
TF42_6wk_k141_136123	1	dsDNAphage	No	High-quality	100	High-quality draft genome	AAI-based (high-confidence)		*Firmicutes*	*Roseburia intestinalis*
TF11_12wk_k141_20235	0.993	dsDNAphage	Yes	High-quality	100	High-quality draft genome	AAI-based (medium-confidence)		*Firmicutes*	*Roseburia intestinalis*
TF47_26wk_k141_63935	0.993	dsDNAphage	No	High-quality	100	High-quality draft genome	AAI-based (high-confidence)			
TF48_6wk_k141_78420	1	dsDNAphage	No	High-quality	100	High-quality draft genome	AAI-based (high-confidence)		*Firmicutes*	*Coprococcus catus*
TM30_6wk_k141_262485	1	dsDNAphage	No	Complete	100	High-quality draft genome	DTR (high-confidence)			
TM35_BL_k141_15263	0.993	dsDNAphage	No	High-quality	99.24	High-quality draft genome	AAI-based (high-confidence)			
TF15_12wk_k141_56504	1	dsDNAphage	No	High-quality	90.73	High-quality draft genome	AAI-based (high-confidence)		*Firmicutes*	*Coprococcus comes*
TF49_6wk_k141_167669	1	dsDNAphage	No	High-quality	94.32	High-quality draft genome	AAI-based (high-confidence)			
TF41_BL_k141_55306	0.973	dsDNAphage	No	High-quality	100	High-quality draft genome	AAI-based (high-confidence)		*Proteobacteria*	*Escherichia coli*
TM09_26wk_k141_1410	1	dsDNAphage	No	High-quality	94.84	High-quality draft genome	AAI-based (high-confidence)		*Firmicutes*	*Anaerostipes hadrus*
TF43_26wk_k141_71876	0.993	dsDNAphage	No	High-quality	98.16	High-quality draft genome	AAI-based (high-confidence)		*Firmicutes*	*Faecalibacterium prausnitzii*
DM03_D1_k141_35998	0.993	dsDNAphage	Yes	High-quality	99.04	High-quality draft genome	AAI-based (high-confidence)			
TF43_BL_k141_151454	0.767	dsDNAphage	No	High-quality	100	High-quality draft genome	AAI-based (high-confidence)			
TF23_6wk_k141_141296	1	dsDNAphage	No	High-quality	99.83	High-quality draft genome	AAI-based (high-confidence)		*Bacteroidetes*	*Prevotella veroralis*
TF06_12wk_k141_6417	1	dsDNAphage	No	High-quality	97.31	High-quality draft genome	AAI-based (high-confidence)	*Crass_like*		
DF14_D7_k141_109096	1	dsDNAphage	No	High-quality	100	High-quality draft genome	AAI-based (high-confidence)			
TM32_12wk_k141_9246	1	dsDNAphage	No	High-quality	96.95	High-quality draft genome	AAI-based (high-confidence)		*Bacteroidetes*	*Bacteroides dorei*
TF42_6wk_k141_36598	0.987	dsDNAphage	No	High-quality	100	High-quality draft genome	AAI-based (high-confidence)		*Actinobacteria*	*Bifidobacterium bifidum*
TM20_BL_k141_168269	1	dsDNAphage	No	High-quality	94.13	High-quality draft genome	AAI-based (high-confidence)			
TM14_BL_k141_46199	0.993	dsDNAphage	No	Complete	100	High-quality draft genome	DTR (high-confidence)			
TF24_12wk_k141_141674	0.96	dsDNAphage	No	High-quality	100	High-quality draft genome	AAI-based (high-confidence)			
TF05_6wk_k141_18557	1	dsDNAphage	No	High-quality	96.06	High-quality draft genome	AAI-based (high-confidence)		*Firmicutes*	*Faecalibacterium prausnitzii*
DM12_D3_k141_10325	1	dsDNAphage	No	Complete	100	High-quality draft genome	DTR (high-confidence)	*Siphoviridae*		
TF01_26wk_k141_119301	0.993	dsDNAphage	No	High-quality	100	High-quality draft genome	AAI-based (high-confidence)			
TF41_BL_k141_59395	1	dsDNAphage	No	High-quality	98.27	High-quality draft genome	AAI-based (high-confidence)	*Myoviridae*		
TF13_BL_k141_9193	0.993	dsDNAphage	Yes	High-quality	100	High-quality draft genome	AAI-based (high-confidence)			
TM27_6wk_k141_47861	1	dsDNAphage	No	High-quality	94.16	High-quality draft genome	AAI-based (high-confidence)	*Siphoviridae*	*Bacteroidetes*	*Bacteroides xylanisolvens*
TM04_6wk_k141_34760	0.987	dsDNAphage	No	High-quality	100	High-quality draft genome	AAI-based (high-confidence)		*Actinobacteria*	*Bifidobacterium bifidum*
TF26_6wk_k141_68204	1	dsDNAphage	No	High-quality	96.71	High-quality draft genome	AAI-based (high-confidence)		*Firmicutes*	*Roseburia intestinalis*
TM04_6wk_k141_62059	0.987	dsDNAphage	No	High-quality	97.43	High-quality draft genome	AAI-based (high-confidence)		*Bacteroidetes*	*Bacteroides uniformis*
TF03_6wk_k141_241988	0.987	dsDNAphage	No	High-quality	100	High-quality draft genome	AAI-based (high-confidence)			
TF36_BL_k141_58164	0.887	ssDNA	No	High-quality	100	High-quality draft genome	HMM-based (lower-bound)			
DM08_D6_k141_124219	0.993	ssDNA	No	High-quality	100	High-quality draft genome	HMM-based (lower-bound)			
TF26_26wk_k141_186839	1	dsDNAphage	No	Complete	100	High-quality draft genome	DTR (high-confidence)		*Firmicutes*	*Blautia obeum*
TF32_6wk_k141_123728	1	dsDNAphage	Yes	High-quality	100	High-quality draft genome	AAI-based (medium-confidence)			
TM09_12wk_k141_114263	1	dsDNAphage	No	Complete	100	High-quality draft genome	DTR (high-confidence)	*Podoviridae*		
TM39_12wk_k141_25349	0.987	dsDNAphage	No	High-quality	100	High-quality draft genome	AAI-based (high-confidence)		*Firmicutes*	*Faecalibacterium prausnitzii*
DM08_D5_k141_160924	1	dsDNAphage	No	High-quality	91.27	High-quality draft genome	AAI-based (high-confidence)			
TF41_26wk_k141_22355	0.993	dsDNAphage	No	High-quality	94.86	High-quality draft genome	AAI-based (high-confidence)		*Firmicutes*	*Ruminococcus bromii*
TF49_6wk_k141_104453	0.787	dsDNAphage	No	High-quality	98.96	High-quality draft genome	AAI-based (medium-confidence)			
TM16_6WK_k141_234568	0.987	dsDNAphage	No	High-quality	100	High-quality draft genome	AAI-based (high-confidence)		*Firmicutes*	*Roseburia inulinivorans*
TF30_6wk_k141_166440	1	dsDNAphage	Yes	High-quality	90.7	High-quality draft genome	AAI-based (medium-confidence)			
TF03_26wk_k141_108973	1	dsDNAphage	No	High-quality	96.1	High-quality draft genome	AAI-based (high-confidence)	*Siphoviridae*		
TM05_BL_k141_107025	1	dsDNAphage	No	High-quality	100	High-quality draft genome	AAI-based (high-confidence)			
TF24_BL_k141_71389	1	dsDNAphage	No	High-quality	100	High-quality draft genome	AAI-based (high-confidence)	*Myoviridae*		
TF09_6WK_k141_10296	0.987	dsDNAphage	No	High-quality	100	High-quality draft genome	AAI-based (high-confidence)	*Siphoviridae*	*Firmicutes*	*Ruminococcus bromii*
TF40_BL_k141_77591	0.993	dsDNAphage	No	High-quality	100	High-quality draft genome	AAI-based (high-confidence)			
TM12_6WK_k141_103922	0.767	ssDNA	No	High-quality	100	High-quality draft genome	HMM-based (lower-bound)			
TF32_6wk_k141_42025	0.987	dsDNAphage	No	High-quality	95.52	High-quality draft genome	AAI-based (high-confidence)		*Firmicutes*	*Faecalibacterium prausnitzii*
TM30_BL_k141_206919	0.64	dsDNAphage	No	High-quality	100	High-quality draft genome	AAI-based (high-confidence)			
TM23_BL_k141_6899	0.987	dsDNAphage	No	High-quality	100	High-quality draft genome	AAI-based (medium-confidence)			
TF37_6wk_k141_147746	0.993	dsDNAphage	No	Complete	100	High-quality draft genome	DTR (high-confidence)		*Firmicutes*	*Blautia obeum*
TF31_12wk_k141_35541	0.993	dsDNAphage	Yes	High-quality	100	High-quality draft genome	AAI-based (high-confidence)			
DM12_D5_k141_47380	0.993	dsDNAphage	No	High-quality	100	High-quality draft genome	AAI-based (high-confidence)	*Myoviridae*		
TF29_6WK_k141_192249	0.98	dsDNAphage	No	High-quality	93.12	High-quality draft genome	AAI-based (high-confidence)			
TF16_BL_k141_30264	0.993	dsDNAphage	No	High-quality	100	High-quality draft genome	AAI-based (high-confidence)			
TF32_BL_k141_42758	0.947	dsDNAphage	No	High-quality	100	High-quality draft genome	AAI-based (medium-confidence)			
TM28_12wk_k141_153361	1	dsDNAphage	No	High-quality	100	High-quality draft genome	AAI-based (high-confidence)			
TF15_12wk_k141_87358	1	dsDNAphage	No	Complete	100	High-quality draft genome	DTR (high-confidence)		*Firmicutes*	*Faecalibacterium prausnitzii*
TM29_6wk_k141_31288	0.993	dsDNAphage	No	High-quality	100	High-quality draft genome	AAI-based (high-confidence)		*Firmicutes*	*Anaerobutyricum hallii*
DF16_D9_k141_288034	1	dsDNAphage	No	Complete	100	High-quality draft genome	DTR (high-confidence)			
TF08_BL_k141_129009	1	dsDNAphage	No	High-quality	97.7	High-quality draft genome	AAI-based (high-confidence)			
DM07_D6_k141_28949	1	dsDNAphage	No	High-quality	93.86	High-quality draft genome	AAI-based (medium-confidence)			
TF41_BL_k141_56275	0.7	dsDNAphage	No	High-quality	100	High-quality draft genome	AAI-based (high-confidence)			
TF08_BL_k141_47815	0.993	dsDNAphage	Yes	High-quality	100	High-quality draft genome	AAI-based (high-confidence)			
TF05_6wk_k141_101786	0.993	dsDNAphage	No	High-quality	94.08	High-quality draft genome	AAI-based (medium-confidence)			
TM07_BL_k141_177779	1	dsDNAphage	No	High-quality	100	High-quality draft genome	AAI-based (high-confidence)			
TF27_12wk_k141_2686	0.993	dsDNAphage	Yes	High-quality	100	High-quality draft genome	AAI-based (high-confidence)		*Firmicutes*	*Coprococcus comes*
TF48_BL_k141_50957	1	dsDNAphage	No	Complete	100	High-quality draft genome	DTR (high-confidence)			
TF22_26wk_k141_49766	0.54	dsDNAphage	No	High-quality	100	High-quality draft genome	AAI-based (high-confidence)			
TM39_12wk_k141_74908	0.967	dsDNAphage	No	Complete	100	High-quality draft genome	DTR (high-confidence)		*Firmicutes*	*Anaerobutyricum hallii*
TM17_6WK_k141_13455	1	dsDNAphage	No	High-quality	100	High-quality draft genome	AAI-based (high-confidence)	*Podoviridae*		
TM16_BL_k141_23190	0.667	dsDNAphage	No	High-quality	100	High-quality draft genome	AAI-based (medium-confidence)			
TM28_6wk_k141_96273	1	dsDNAphage	No	High-quality	96.85	High-quality draft genome	AAI-based (high-confidence)			
TF32_6wk_k141_23758	0.96	dsDNAphage	Yes	High-quality	100	High-quality draft genome	AAI-based (high-confidence)		*Firmicutes*	*Dorea longicatena*
TF45_26wk_k141_50684	0.96	dsDNAphage	No	High-quality	100	High-quality draft genome	AAI-based (high-confidence)		*Bacteroidetes*	*Bacteroides nordii*
TF32_12wk_k141_29569	0.953	dsDNAphage	No	High-quality	100	High-quality draft genome	AAI-based (high-confidence)			
DM05_D1_k141_124655	1	dsDNAphage	No	Complete	100	High-quality draft genome	DTR (high-confidence)			
TF32_12wk_k141_5005	1	dsDNAphage	No	High-quality	100	High-quality draft genome	AAI-based (high-confidence)		*Firmicutes*	*Tyzzerella nexilis*
TM06_26wk_k141_78863	1	dsDNAphage	No	Complete	100	High-quality draft genome	DTR (high-confidence)		*Firmicutes*	*Blautia obeum*
TF23_BL_k141_75286	1	dsDNAphage	No	High-quality	100	High-quality draft genome	AAI-based (high-confidence)			
TF42_6wk_k141_61314	1	dsDNAphage	No	High-quality	94.32	High-quality draft genome	AAI-based (high-confidence)			
TF04_BL_k141_115524	0.993	dsDNAphage	No	High-quality	97.23	High-quality draft genome	AAI-based (high-confidence)			
TM16_6WK_k141_248566	1	dsDNAphage	No	High-quality	100	High-quality draft genome	AAI-based (high-confidence)			
DM12_D3_k141_33250	0.993	dsDNAphage	Yes	High-quality	99.17	High-quality draft genome	AAI-based (high-confidence)	*Myoviridae*		
TF39_12wk_k141_91660	0.987	dsDNAphage	No	High-quality	100	High-quality draft genome	AAI-based (high-confidence)			
TM36_12wk_k141_126385	1	dsDNAphage	No	High-quality	98.49	High-quality draft genome	AAI-based (high-confidence)		*Bacteroidetes*	*Prevotella copri*
TM30_BL_k141_244284	1	dsDNAphage	No	High-quality	99.04	High-quality draft genome	AAI-based (high-confidence)			
TF48_BL_k141_193477	0.893	dsDNAphage	No	High-quality	100	High-quality draft genome	AAI-based (high-confidence)			
TF33_BL_k141_164065	0.98	dsDNAphage	No	High-quality	100	High-quality draft genome	AAI-based (high-confidence)	*Siphoviridae*	*Proteobacteria*	*Escherichia coli*
TM24_6wk_k141_114866	0.993	dsDNAphage	No	High-quality	100	High-quality draft genome	AAI-based (high-confidence)			
TF37_6wk_k141_114639	1	dsDNAphage	No	High-quality	96.35	High-quality draft genome	AAI-based (high-confidence)			
TF43_12wk_k141_183329	0.933	dsDNAphage	No	High-quality	100	High-quality draft genome	HMM-based (lower-bound)			
TM05_6wk_k141_136407	0.98	dsDNAphage	No	High-quality	100	High-quality draft genome	AAI-based (high-confidence)			
DM12_D3_k141_149327	1	dsDNAphage	No	Complete	100	High-quality draft genome	DTR (high-confidence)			
TM23_BL_k141_77532	0.993	dsDNAphage	No	High-quality	100	High-quality draft genome	AAI-based (high-confidence)	*Myoviridae*		
TM35_BL_k141_50710	0.993	dsDNAphage	No	Complete	100	High-quality draft genome	DTR (high-confidence)			
TF04_BL_k141_169960	1	dsDNAphage	No	Complete	100	High-quality draft genome	DTR (high-confidence)	*Crass_like*		
TM34_26wk_k141_60367	0.713	dsDNAphage	No	High-quality	100	High-quality draft genome	HMM-based (lower-bound)			

UViG	VirSorter2 score	Predicted genome type	Provirus	Completeness quality	Completeness (%)	Assembly quality	Completeness approach	Family	Host phylum	Host species
TF38_6wk_k141_141002	1	dsDNAphage	No	High-quality	100	High-quality draft genome	AAI-based (medium-confidence)		*Firmicutes*	*Ruminococcus bromii*
TF05_12wk_k141_82869	0.993	dsDNAphage	No	High-quality	99.79	High-quality draft genome	AAI-based (high-confidence)		*Actinobacteria*	*Bifidobacterium adolescentis*
TM19_BL_k141_64230	1	dsDNAphage	No	High-quality	94.1	High-quality draft genome	AAI-based (high-confidence)			
TM05_12wk_k141_175843	0.993	ssDNA	No	Complete	100	High-quality draft genome	DTR (high-confidence)	*Microviridae*	*Bacteroidetes*	*Prevotella disiens*
TF02_6wk_k141_181592	0.987	dsDNAphage	No	High-quality	100	High-quality draft genome	AAI-based (high-confidence)	*Microviridae*		
TF27_6wk_k141_8247	0.993	dsDNAphage	No	Complete	100	High-quality draft genome	DTR (high-confidence)		*Firmicutes*	*Blautia obeum*
TF18_6wk_k141_92629	1	dsDNAphage	No	High-quality	93.53	High-quality draft genome	AAI-based (high-confidence)			
TF43_BL_k141_26960	0.987	dsDNAphage	No	Complete	100	High-quality draft genome	DTR (high-confidence)		*Firmicutes*	*Roseburia intestinalis*
TF48_26wk_k141_48243	0.993	dsDNAphage	No	High-quality	99.3	High-quality draft genome	AAI-based (high-confidence)			
TF44_12wk_k141_105166	0.993	dsDNAphage	No	High-quality	95.85	High-quality draft genome	AAI-based (high-confidence)		*Firmicutes*	*Faecalibacterium prausnitzii*
DF16_D5_k141_255005	0.987	dsDNAphage	No	High-quality	100	High-quality draft genome	AAI-based (high-confidence)			
TF33_BL_k141_175106	1	dsDNAphage	No	High-quality	100	High-quality draft genome	AAI-based (medium-confidence)			
TF43_12wk_k141_112503	0.993	dsDNAphage	No	High-quality	90.34	High-quality draft genome	AAI-based (high-confidence)		*Firmicutes*	*Faecalibacterium prausnitzii*
TM25_26wk_k141_11448[illegible]	0.987	dsDNAphage	No	Complete	100	High-quality draft genome	DTR (high-confidence)			
TF24_12wk_k141_88094	0.993	dsDNAphage	No	High-quality	100	High-quality draft genome	AAI-based (high-confidence)		*Firmicutes*	*Ruminococcus bromii*
TF45_BL_k141_47053	0.987	dsDNAphage	No	High-quality	100	High-quality draft genome	AAI-based (high-confidence)	*Microviridae*		
TF16_12wk_k141_104461	0.993	dsDNAphage	Yes	High-quality	100	High-quality draft genome	AAI-based (medium-confidence)	*Podoviridae*		
TM23_BL_k141_57832	0.9	dsDNAphage	No	High-quality	100	High-quality draft genome	AAI-based (high-confidence)			
TF37_6wk_k141_134014	1	dsDNAphage	No	High-quality	93.85	High-quality draft genome	AAI-based (high-confidence)		*Bacteroidetes*	*Bacteroides stercoris*
DM07_D1_k141_126862	0.993	dsDNAphage	No	Complete	100	High-quality draft genome	DTR (high-confidence)			
TM29_12wk_k141_15508	0.987	dsDNAphage	No	High-quality	100	High-quality draft genome	AAI-based (high-confidence)		*Firmicutes*	*Blautia obeum*
TF50_12wk_k141_184527	1	dsDNAphage	No	Complete	100	High-quality draft genome	DTR (high-confidence)		*Bacteroidetes*	*Prevotella timonensis*
DF16_D6_k141_88966	0.993	dsDNAphage	No	High-quality	100	High-quality draft genome	AAI-based (medium-confidence)			
TM30_6wk_k141_2632	1	dsDNAphage	No	Complete	100	High-quality draft genome	DTR (high-confidence)			
TF32_6wk_k141_75754	1	dsDNAphage	Yes	High-quality	99.94	High-quality draft genome	AAI-based (high-confidence)		*Firmicutes*	*Streptococcus constellatus*
DF14_D2_k141_113350	0.993	dsDNAphage	No	Complete	100	High-quality draft genome	DTR (high-confidence)	*Siphoviridae*		
TF27_26wk_k141_60934	0.993	dsDNAphage	No	High-quality	100	High-quality draft genome	AAI-based (high-confidence)			
TF10_BL_k141_174044	0.993	dsDNAphage	No	Complete	100	High-quality draft genome	DTR (medium-confidence)	*Siphoviridae*		
TF16_26wk_k141_3488	1	dsDNAphage	No	High-quality	100	High-quality draft genome	AAI-based (high-confidence)		*Bacteroidetes*	*Prevotella bivia*
TF28_12wk_k141_45624	1	dsDNAphage	No	High-quality	100	High-quality draft genome	AAI-based (high-confidence)		*Bacteroidetes*	*Prevotella copri*
TF04_BL_k141_69875	1	dsDNAphage	No	High-quality	93.42	High-quality draft genome	AAI-based (high-confidence)			
TF12_26wk_k141_54118	1	dsDNAphage	Yes	High-quality	100	High-quality draft genome	AAI-based (high-confidence)	*Myoviridae*	*Firmicutes*	*Faecalibacterium prausnitzii*
TF30_BL_k141_6116	1	dsDNAphage	No	High-quality	97.94	High-quality draft genome	AAI-based (high-confidence)	*Siphoviridae*		
TF35_BL_k141_9170	0.993	dsDNAphage	Yes	High-quality	100	High-quality draft genome	AAI-based (medium-confidence)			
TM34_26wk_k141_40405	1	dsDNAphage	No	High-quality	100	High-quality draft genome	AAI-based (high-confidence)			
TF28_6wk_k141_4884	1	dsDNAphage	No	High-quality	100	High-quality draft genome	AAI-based (high-confidence)			
TM28_6wk_k141_92215	0.993	dsDNAphage	No	High-quality	100	High-quality draft genome	AAI-based (high-confidence)		*Actinobacteria*	*Bifidobacterium longum*
DF16_D9_k141_170555	0.987	dsDNAphage	Yes	Complete	100	High-quality draft genome	Provirus (high-confidence)			
TF37_12wk_k141_109543	1	dsDNAphage	No	High-quality	93.3	High-quality draft genome	AAI-based (high-confidence)		*Bacteroidetes*	*Bacteroides xylanisolvens*
TF33_6wk_k141_35434	0.973	dsDNAphage	No	Complete	100	High-quality draft genome	DTR (high-confidence)			
DM07_D5_k141_194492	1	dsDNAphage	No	High-quality	97.9	High-quality draft genome	AAI-based (high-confidence)			
TF11_26wk_k141_130760	0.993	dsDNAphage	Yes	High-quality	100	High-quality draft genome	AAI-based (high-confidence)		*Firmicutes*	*Staphylococcus aureus*
DM08_D6_k141_97169	0.987	dsDNAphage	Yes	High-quality	100	High-quality draft genome	AAI-based (high-confidence)			
TM13_26wk_k141_19828[illegible]	1	dsDNAphage	No	High-quality	100	High-quality draft genome	AAI-based (high-confidence)		*Firmicutes*	*Faecalibacterium prausnitzii*
TM24_BL_k141_25888	0.993	dsDNAphage	No	High-quality	96.3	High-quality draft genome	AAI-based (high-confidence)			
TF20_12wk_k141_137878	0.933	dsDNAphage	No	High-quality	100	High-quality draft genome	AAI-based (high-confidence)			
DF16a_D4_k141_51821	0.64	dsDNAphage	No	High-quality	100	High-quality draft genome	AAI-based (medium-confidence)			
TM23_BL_k141_127515	1	dsDNAphage	No	Complete	100	High-quality draft genome	DTR (high-confidence)			
DM08_D2_k141_173461	0.827	dsDNAphage	No	High-quality	100	High-quality draft genome	AAI-based (high-confidence)			
DF16_D2_k141_264002	0.993	dsDNAphage	Yes	High-quality	100	High-quality draft genome	AAI-based (high-confidence)			
DF14_D7_k141_99527	1	dsDNAphage	No	Complete	100	High-quality draft genome	DTR (high-confidence)	*Myoviridae*		
DF17_D8_k141_139493	0.953	dsDNAphage	Yes	High-quality	90.02	High-quality draft genome	AAI-based (high-confidence)			
TM06_BL_k141_126282	1	dsDNAphage	No	High-quality	99.08	High-quality draft genome	AAI-based (medium-confidence)			
TF06_6WK_k141_197893	0.987	dsDNAphage	No	Complete	100	High-quality draft genome	DTR (high-confidence)		*Firmicutes*	*Faecalibacterium prausnitzii*
TF12_26wk_k141_41173	1	dsDNAphage	No	Complete	100	High-quality draft genome	DTR (high-confidence)			
TM27_26wk_k141_143112	0.98	dsDNAphage	No	Complete	100	High-quality draft genome	DTR (high-confidence)			
TM28_12wk_k141_57905	0.987	dsDNAphage	No	High-quality	100	High-quality draft genome	AAI-based (high-confidence)		*Firmicutes*	*Dorea formicigenerans*
TF30_6wk_k141_191726	0.993	dsDNAphage	Yes	High-quality	100	High-quality draft genome	AAI-based (high-confidence)		*Firmicutes*	*Faecalibacterium prausnitzii*
TF18_BL_k141_171432	1	dsDNAphage	No	Complete	100	High-quality draft genome	DTR (high-confidence)			
DF12_D5_k141_68866	0.993	dsDNAphage	Yes	Complete	100	High-quality draft genome	Provirus (high-confidence)			
TF18_6wk_k141_51530	0.987	dsDNAphage	No	High-quality	100	High-quality draft genome	AAI-based (high-confidence)			
TF32_26wk_k141_122583	0.973	dsDNAphage	No	High-quality	100	High-quality draft genome	AAI-based (high-confidence)			
DM08_D6_k141_102573	0.993	dsDNAphage	No	High-quality	100	High-quality draft genome	AAI-based (high-confidence)			
TF32_BL_k141_166984	0.893	dsDNAphage	No	High-quality	100	High-quality draft genome	AAI-based (high-confidence)			
TM24_6wk_k141_221350	0.993	dsDNAphage	No	Complete	100	High-quality draft genome	DTR (high-confidence)			
TF41_12wk_k141_34055	0.987	dsDNAphage	No	High-quality	100	High-quality draft genome	AAI-based (high-confidence)		*Firmicutes*	*Ruminococcus champanellensis*
TM12_6WK_k141_72406	1	dsDNAphage	No	High-quality	100	High-quality draft genome	AAI-based (high-confidence)		*Firmicutes*	*Faecalibacterium prausnitzii*
TM17_12wk_k141_151255	0.993	dsDNAphage	No	Complete	100	High-quality draft genome	DTR (high-confidence)		*Firmicutes*	*Blautia obeum*
TF50_BL_k141_157761	1	dsDNAphage	No	Complete	100	High-quality draft genome	DTR (high-confidence)	*Podoviridae*		
TM10_BL_k141_15129	0.993	dsDNAphage	No	Complete	100	High-quality draft genome	DTR (high-confidence)	*Siphoviridae*		
TM07_12wk_k141_142355	0.987	dsDNAphage	No	High-quality	100	High-quality draft genome	AAI-based (high-confidence)		*Firmicutes*	*Anaerobutyricum hallii*
TM04_12wk_k141_17859	1	dsDNAphage	No	High-quality	99.49	High-quality draft genome	AAI-based (medium-confidence)			
TF44_6wk_k141_46839	1	dsDNAphage	No	High-quality	100	High-quality draft genome	AAI-based (high-confidence)		*Firmicutes*	*Anaerostipes hadrus*
TF30_BL_k141_115070	0.993	dsDNAphage	No	Complete	100	High-quality draft genome	DTR (high-confidence)			
DM03_D4_k141_72208	0.987	dsDNAphage	No	High-quality	90.53	High-quality draft genome	AAI-based (medium-confidence)			
TF32_BL_k141_103813	0.727	dsDNAphage	No	High-quality	100	High-quality draft genome	AAI-based (medium-confidence)			
TM22_12wk_k141_10484	1	dsDNAphage	No	High-quality	100	High-quality draft genome	AAI-based (high-confidence)		*Actinobacteria*	*Bifidobacterium longum*
TF20_BL_k141_53272	0.967	dsDNAphage	No	High-quality	100	High-quality draft genome	AAI-based (high-confidence)			
TM25_26wk_k141_88015	1	dsDNAphage	No	High-quality	100	High-quality draft genome	AAI-based (high-confidence)	*Crass_like*	*Bacteroidetes*	*Bacteroides xylanisolvens*
TF47_6wk_k141_201604	0.967	dsDNAphage	No	High-quality	100	High-quality draft genome	AAI-based (high-confidence)			
DF16b_D4_k141_88970	1	dsDNAphage	Yes	High-quality	95.62	High-quality draft genome	AAI-based (high-confidence)		*Bacteroidetes*	*Prevotella disiens*
TF43_26wk_k141_176960	1	dsDNAphage	No	High-quality	93.3	High-quality draft genome	AAI-based (high-confidence)		*Actinobacteria*	*Bifidobacterium longum*
TF37_26wk_k141_120171	0.987	dsDNAphage	Yes	High-quality	100	High-quality draft genome	AAI-based (high-confidence)		*Bacteroidetes*	*Bacteroides ovatus*
TM14_6wk_k141_32763	0.993	dsDNAphage	No	High-quality	98.73	High-quality draft genome	AAI-based (high-confidence)			
TF41_BL_k141_83801	0.987	dsDNAphage	No	High-quality	100	High-quality draft genome	AAI-based (high-confidence)			
TM05_12wk_k141_92972	0.993	dsDNAphage	No	Complete	100	High-quality draft genome	DTR (high-confidence)		*Firmicutes*	*Streptococcus pneumoniae*
TF33_BL_k141_136424	1	dsDNAphage	Yes	High-quality	100	High-quality draft genome	AAI-based (high-confidence)			
TF28_6wk_k141_38058	0.993	dsDNAphage	No	High-quality	96.71	High-quality draft genome	AAI-based (high-confidence)		*Firmicutes*	*Dorea longicatena*
TF38_12wk_k141_242282	0.987	dsDNAphage	No	Complete	100	High-quality draft genome	DTR (high-confidence)		*Firmicutes*	*Faecalibacterium prausnitzii*
TM20_12wk_k141_44978	0.993	dsDNAphage	No	High-quality	100	High-quality draft genome	AAI-based (high-confidence)			
TM20_6wk_k141_109637	0.993	dsDNAphage	No	High-quality	98.36	High-quality draft genome	AAI-based (high-confidence)			
DF12_D2_k141_76088	1	dsDNAphage	No	High-quality	100	High-quality draft genome	AAI-based (high-confidence)			
TM13_26wk_k141_160912	0.987	dsDNAphage	No	High-quality	100	High-quality draft genome	AAI-based (high-confidence)		*Firmicutes*	*Eubacterium ventriosum*
DF12_D3_k141_63948	1	dsDNAphage	No	High-quality	95.89	High-quality draft genome	AAI-based (high-confidence)			
TM33_6wk_k141_108591	0.987	dsDNAphage	No	High-quality	100	High-quality draft genome	AAI-based (medium-confidence)	*Podoviridae*		
TM16_12wk_k141_222452	1	dsDNAphage	No	High-quality	100	High-quality draft genome	AAI-based (high-confidence)		*Firmicutes*	*Roseburia intestinalis*
TF29_6WK_k141_179007	0.987	dsDNAphage	No	High-quality	100	High-quality draft genome	AAI-based (high-confidence)			
TM15_BL_k141_13658	0.993	dsDNAphage	No	High-quality	100	High-quality draft genome	AAI-based (high-confidence)			
TF06_BL_k141_20226	1	dsDNAphage	No	High-quality	100	High-quality draft genome	AAI-based (medium-confidence)			
TF45_6wk_k141_54433	0.993	dsDNAphage	No	High-quality	93.07	High-quality draft genome	AAI-based (high-confidence)		*Firmicutes*	*Roseburia inulinivorans*
TF37_BL_k141_33504	0.993	dsDNAphage	No	High-quality	100	High-quality draft genome	AAI-based (high-confidence)			
TM30_26wk_k141_10322[illegible]	0.987	dsDNAphage	No	High-quality	100	High-quality draft genome	AAI-based (high-confidence)		*Firmicutes*	*Blautia obeum*
TM21_12wk_k141_55236	1	dsDNAphage	No	Complete	100	High-quality draft genome	DTR (high-confidence)		*Firmicutes*	*Faecalibacterium prausnitzii*
TM35_BL_k141_232929	1	dsDNAphage	No	High-quality	100	High-quality draft genome	AAI-based (high-confidence)			
TM02_BL_k141_109066	0.94	ssDNA	No	High-quality	100	High-quality draft genome	HMM-based (lower-bound)			

UViG	VirSorter2 score	Predicted genome type	Provirus	Completeness quality	Completeness (%)	Assembly quality	Completeness approach	Family	Host phylum	Host species
TM30_12wk_k141_43651	0.993	dsDNAphage	No	High-quality	100	High-quality draft genome	AAI-based (high-confidence)		*Firmicutes*	*Butyrivibrio crossotus*
TF37_BL_k141_166384	0.993	dsDNAphage	No	Complete	100	High-quality draft genome	DTR (high-confidence)	*Siphoviridae*		
TM05_BL_k141_122283	1	dsDNAphage	No	Complete	100	High-quality draft genome	DTR (high-confidence)			
TF21_26wk_k141_24858	0.993	dsDNAphage	No	High-quality	96.7	High-quality draft genome	AAI-based (high-confidence)			
TF37_12wk_k141_94607	0.993	dsDNAphage	No	Complete	100	High-quality draft genome	DTR (high-confidence)			
TF04_BL_k141_15741	0.993	dsDNAphage	No	High-quality	100	High-quality draft genome	AAI-based (high-confidence)			
TF20_BL_k141_79461	0.993	dsDNAphage	No	High-quality	100	High-quality draft genome	AAI-based (high-confidence)			
TF08_BL_k141_71550	0.993	dsDNAphage	No	High-quality	91.49	High-quality draft genome	AAI-based (high-confidence)			
TF29_BL_k141_1434	0.973	dsDNAphage	No	Complete	100	High-quality draft genome	DTR (high-confidence)			
TF49_BL_k141_92403	1	dsDNAphage	No	High-quality	92.96	High-quality draft genome	AAI-based (high-confidence)		*Bacteroidetes*	*Bacteroides vulgatus*
TM19_BL_k141_96364	1	dsDNAphage	No	High-quality	100	High-quality draft genome	AAI-based (high-confidence)			
TF16_BL_k141_70513	0.987	dsDNAphage	No	High-quality	100	High-quality draft genome	AAI-based (high-confidence)	*Myoviridae*		
TF19_26wk_k141_198265	0.993	ssDNA	No	High-quality	100	High-quality draft genome	HMM-based (lower-bound)			
TF01_6wk_k141_130802	0.933	ssDNA	No	High-quality	100	High-quality draft genome	HMM-based (lower-bound)			
DM08_D5_k141_7795	1	dsDNAphage	No	Complete	100	High-quality draft genome	DTR (high-confidence)			
TM25_26wk_k141_48917	0.98	dsDNAphage	No	High-quality	100	High-quality draft genome	AAI-based (medium-confidence)		*Proteobacteria*	*Bilophila wadsworthia*
TM17_12wk_k141_29044	1	dsDNAphage	No	High-quality	92.17	High-quality draft genome	AAI-based (high-confidence)		*Firmicutes*	*Faecalibacterium prausnitzii*
TM05_6wk_k141_138539	1	dsDNAphage	No	Complete	100	High-quality draft genome	DTR (high-confidence)		*Bacteroidetes*	*Prevotella copri*
TF34_6wk_k141_13267	1	dsDNAphage	No	High-quality	97.43	High-quality draft genome	AAI-based (high-confidence)		*Firmicutes*	*Faecalibacterium prausnitzii*
TF34_12wk_k141_130865	0.973	dsDNAphage	No	High-quality	100	High-quality draft genome	AAI-based (high-confidence)		*Firmicutes*	*Dialister invisus*
DF17_D4_k141_66600	1	dsDNAphage	No	High-quality	100	High-quality draft genome	AAI-based (high-confidence)			
TF25_26wk_k141_81962	1	dsDNAphage	Yes	High-quality	100	High-quality draft genome	AAI-based (high-confidence)			
TF12_6wk_k141_8226	1	dsDNAphage	Yes	High-quality	100	High-quality draft genome	AAI-based (high-confidence)		*Bacteroidetes*	*Bacteroides ovatus*
TM37_BL_k141_3065	1	dsDNAphage	No	High-quality	92.6	High-quality draft genome	AAI-based (high-confidence)			
TM22_26wk_k141_48107	1	dsDNAphage	No	High-quality	100	High-quality draft genome	AAI-based (high-confidence)		*Bacteroidetes*	*Prevotella multiformis*
TM24_12wk_k141_118915	0.667	dsDNAphage	No	High-quality	100	High-quality draft genome	AAI-based (medium-confidence)			
TM16_BL_k141_224760	0.987	dsDNAphage	No	Complete	100	High-quality draft genome	DTR (high-confidence)			
DM07_D6_k141_36597	0.993	dsDNAphage	No	High-quality	100	High-quality draft genome	AAI-based (high-confidence)			
TM06_26wk_k141_16095[illegible]	0.993	dsDNAphage	No	Complete	100	High-quality draft genome	DTR (high-confidence)		*Firmicutes*	*Butyrivibrio crossotus*
DF14_D3_k141_17632	0.753	dsDNAphage	No	High-quality	100	High-quality draft genome	AAI-based (medium-confidence)			
DF17_D8_k141_144264	0.953	dsDNAphage	No	High-quality	100	High-quality draft genome	AAI-based (high-confidence)	*Microviridae*		
TF45_26wk_k141_33946	1	dsDNAphage	No	Complete	100	High-quality draft genome	DTR (high-confidence)			
TF20_12wk_k141_131965	0.993	dsDNAphage	No	High-quality	100	High-quality draft genome	AAI-based (high-confidence)			
TF19_26wk_k141_28863	0.893	dsDNAphage	No	High-quality	100	High-quality draft genome	AAI-based (high-confidence)			
TF21_26wk_k141_14522	1	dsDNAphage	No	High-quality	100	High-quality draft genome	AAI-based (high-confidence)		*Firmicutes*	*Ruminococcus bromii*
DF14_D3_k141_54474	0.993	dsDNAphage	Yes	High-quality	100	High-quality draft genome	AAI-based (high-confidence)			
TM03_12wk_k141_41255	0.993	dsDNAphage	No	High-quality	96.03	High-quality draft genome	AAI-based (high-confidence)			
TF32_26wk_k141_27678	0.967	ssDNA	No	High-quality	100	High-quality draft genome	HMM-based (lower-bound)			
TF19_BL_k141_40941	0.993	dsDNAphage	No	High-quality	98.09	High-quality draft genome	AAI-based (high-confidence)			
TF14_6wk_k141_124765	0.98	dsDNAphage	No	High-quality	100	High-quality draft genome	AAI-based (high-confidence)		*Bacteroidetes*	*Prevotella disiens*
TF20_12wk_k141_195392	0.973	dsDNAphage	No	High-quality	100	High-quality draft genome	AAI-based (high-confidence)			
DF14_D4_k141_96687	0.987	dsDNAphage	No	High-quality	100	High-quality draft genome	AAI-based (medium-confidence)			
TM15_BL_k141_112441	0.987	dsDNAphage	No	High-quality	91.92	High-quality draft genome	AAI-based (high-confidence)			
TM16_12wk_k141_254331	0.94	dsDNAphage	No	Complete	100	High-quality draft genome	DTR (high-confidence)			
TF04_BL_k141_144777	1	dsDNAphage	No	High-quality	94.39	High-quality draft genome	AAI-based (high-confidence)			
TF15_BL_k141_40241	0.993	dsDNAphage	No	High-quality	100	High-quality draft genome	AAI-based (high-confidence)	*Microviridae*		
TF22_12wk_k141_152484	1	dsDNAphage	No	High-quality	95.29	High-quality draft genome	AAI-based (high-confidence)		*Firmicutes*	*Roseburia intestinalis*
TM08_6wk_k141_78781	0.993	dsDNAphage	No	High-quality	100	High-quality draft genome	AAI-based (high-confidence)	*Myoviridae*		
TF17_BL_k141_20504	1	dsDNAphage	No	Complete	100	High-quality draft genome	DTR (high-confidence)			
TF45_6wk_k141_67386	0.973	dsDNAphage	No	High-quality	100	High-quality draft genome	AAI-based (high-confidence)			
TF24_12wk_k141_37755	1	dsDNAphage	Yes	High-quality	100	High-quality draft genome	AAI-based (high-confidence)			
TF44_6wk_k141_7097	0.993	dsDNAphage	No	High-quality	100	High-quality draft genome	HMM-based (lower-bound)			
TF27_12wk_k141_46313	1	dsDNAphage	Yes	High-quality	100	High-quality draft genome	AAI-based (high-confidence)		*Firmicutes*	*Dorea longicatena*
TF19_BL_k141_128186	1	dsDNAphage	No	High-quality	92.21	High-quality draft genome	AAI-based (medium-confidence)		*Bacteroidetes*	*Bacteroides stercoris*
DM12_D3_k141_8054	0.98	dsDNAphage	Yes	High-quality	100	High-quality draft genome	AAI-based (high-confidence)			
TM03_12wk_k141_11288[illegible]	0.993	dsDNAphage	No	Complete	100	High-quality draft genome	DTR (high-confidence)		*Firmicutes*	*Blautia obeum*
TM39_26wk_k141_60199	1	dsDNAphage	No	High-quality	97.08	High-quality draft genome	AAI-based (high-confidence)			
TM15_BL_k141_159356	1	dsDNAphage	No	High-quality	96.33	High-quality draft genome	AAI-based (medium-confidence)			
TF40_12wk_k141_65235	1	dsDNAphage	No	High-quality	92.58	High-quality draft genome	AAI-based (high-confidence)			
TM03_26wk_k141_13918[illegible]	1	dsDNAphage	No	High-quality	97.92	High-quality draft genome	AAI-based (high-confidence)	*Crass_like*	*Firmicutes*	*Staphylococcus aureus*
TM04_12wk_k141_64301	1	dsDNAphage	No	High-quality	100	High-quality draft genome	AAI-based (high-confidence)	*Myoviridae*	*Firmicutes*	*Roseburia inulinivorans*
TF44_BL_k141_2963	0.987	dsDNAphage	No	Complete	100	High-quality draft genome	DTR (high-confidence)	*Microviridae*		
TF06_26wk_k141_191656	1	dsDNAphage	No	Complete	100	High-quality draft genome	DTR (high-confidence)		*Firmicutes*	*Staphylococcus aureus*
TF48_6wk_k141_153623	0.993	dsDNAphage	No	High-quality	99.91	High-quality draft genome	AAI-based (high-confidence)			
TF30_BL_k141_7378	1	dsDNAphage	No	Complete	100	High-quality draft genome	DTR (high-confidence)			
TF21_12wk_k141_101072	1	dsDNAphage	Yes	High-quality	100	High-quality draft genome	AAI-based (high-confidence)		*Firmicutes*	*Faecalibacterium prausnitzii*
TF40_12wk_k141_111014	1	dsDNAphage	No	High-quality	100	High-quality draft genome	AAI-based (high-confidence)	*Siphoviridae*		
TF23_12wk_k141_39847	0.947	dsDNAphage	No	High-quality	100	High-quality draft genome	HMM-based (lower-bound)			
TF33_6wk_k141_179667	1	dsDNAphage	No	Complete	100	High-quality draft genome	DTR (high-confidence)	*Crass_like*	*Firmicutes*	*Staphylococcus aureus*
TF20_26wk_k141_137534	0.993	dsDNAphage	No	High-quality	100	High-quality draft genome	AAI-based (high-confidence)			
TF38_6wk_k141_59732	0.993	dsDNAphage	No	High-quality	100	High-quality draft genome	AAI-based (high-confidence)			
TF05_26wk_k141_98900	0.993	dsDNAphage	No	High-quality	100	High-quality draft genome	AAI-based (high-confidence)			
DM03_D6_k141_18204	1	dsDNAphage	No	High-quality	100	High-quality draft genome	AAI-based (high-confidence)			
TF32_12wk_k141_85142	0.98	dsDNAphage	Yes	High-quality	98.07	High-quality draft genome	AAI-based (high-confidence)		*Firmicutes*	*Faecalibacterium prausnitzii*
TF49_26wk_k141_137702	1	dsDNAphage	No	High-quality	100	High-quality draft genome	AAI-based (high-confidence)		*Firmicutes*	*Streptococcus constellatus*
TF32_12wk_k141_97667	0.993	dsDNAphage	Yes	Complete	100	High-quality draft genome	Provirus (medium-confidence)		*Firmicutes*	*Faecalibacterium prausnitzii*
TF20_12wk_k141_80255	0.993	dsDNAphage	No	High-quality	100	High-quality draft genome	AAI-based (high-confidence)			
TF38_6wk_k141_4238	0.987	dsDNAphage	No	High-quality	100	High-quality draft genome	AAI-based (high-confidence)		*Firmicutes*	*Roseburia intestinalis*
TF21_12wk_k141_236017	0.953	dsDNAphage	No	High-quality	100	High-quality draft genome	AAI-based (high-confidence)		*Firmicutes*	*Faecalibacterium prausnitzii*
TM25_6wk_k141_84989	0.98	dsDNAphage	No	High-quality	100	High-quality draft genome	AAI-based (high-confidence)		*Firmicutes*	*Blautia obeum*
TF09_BL_k141_183195	1	dsDNAphage	No	High-quality	91.78	High-quality draft genome	AAI-based (high-confidence)			
DF17_D3_k141_38721	1	dsDNAphage	No	High-quality	100	High-quality draft genome	AAI-based (high-confidence)			
DF16_D5_k141_238761	0.767	dsDNAphage	No	High-quality	99.95	High-quality draft genome	AAI-based (high-confidence)			
DF16_D5_k141_209532	0.84	ssDNA	No	High-quality	100	High-quality draft genome	HMM-based (lower-bound)			
DM12_D6_k141_122806	1	dsDNAphage	No	High-quality	90.99	High-quality draft genome	AAI-based (high-confidence)			
TM16_12wk_k141_102187	0.987	dsDNAphage	No	Complete	100	High-quality draft genome	DTR (high-confidence)			
TF39_6wk_k141_147909	1	dsDNAphage	No	High-quality	100	High-quality draft genome	AAI-based (high-confidence)			
DF16_D6_k141_20648	0.96	dsDNAphage	No	High-quality	90.71	High-quality draft genome	AAI-based (high-confidence)	*Siphoviridae*		
TF45_26wk_k141_10335	0.993	dsDNAphage	No	Complete	100	High-quality draft genome	DTR (high-confidence)	*Crass_like*	*Bacteroidetes*	*Parabacteroides distasonis*
TF15_BL_k141_119305	1	dsDNAphage	Yes	High-quality	100	High-quality draft genome	AAI-based (high-confidence)			
TF36_6wk_k141_162382	1	dsDNAphage	No	Complete	100	High-quality draft genome	DTR (high-confidence)		*Bacteroidetes*	*Prevotella copri*
TF34_6wk_k141_267756	1	dsDNAphage	Yes	High-quality	99.72	High-quality draft genome	AAI-based (high-confidence)		*Firmicutes*	*Butyrivibrio crossotus*
TF51_6wk_k141_45771	1	dsDNAphage	No	Complete	100	High-quality draft genome	DTR (high-confidence)		*Firmicutes*	*Blautia obeum*
TM27_12wk_k141_179423	0.993	dsDNAphage	No	High-quality	100	High-quality draft genome	AAI-based (high-confidence)		*Firmicutes*	*Dorea longicatena*
DF14_D5_k141_12684	0.993	dsDNAphage	No	High-quality	100	High-quality draft genome	AAI-based (high-confidence)			
TF29_BL_k141_42158	0.993	dsDNAphage	Yes	High-quality	100	High-quality draft genome	AAI-based (high-confidence)	*Siphoviridae*		
DF16_D9_k141_287896	1	dsDNAphage	No	Complete	100	High-quality draft genome	DTR (medium-confidence)			
TF13_26wk_k141_190997	0.98	dsDNAphage	No	High-quality	100	High-quality draft genome	AAI-based (high-confidence)	*Microviridae*		
TM08_6wk_k141_166189	0.82	dsDNAphage	No	High-quality	100	High-quality draft genome	HMM-based (lower-bound)			
TM09_26wk_k141_56815	1	dsDNAphage	No	High-quality	94.5	High-quality draft genome	AAI-based (medium-confidence)		*Firmicutes*	*Anaerostipes hadrus*
TF49_BL_k141_147319	0.987	dsDNAphage	No	Complete	100	High-quality draft genome	ITR (high-confidence)			
DM08_D2_k141_48681	0.993	dsDNAphage	No	High-quality	100	High-quality draft genome	AAI-based (high-confidence)			
DF17_D4_k141_131391	1	dsDNAphage	No	High-quality	91.97	High-quality draft genome	AAI-based (high-confidence)	*Siphoviridae*		
TF33_BL_k141_43709	0.993	dsDNAphage	No	Complete	100	High-quality draft genome	DTR (medium-confidence)			
DF16_D2_k141_32125	0.987	dsDNAphage	No	High-quality	100	High-quality draft genome	AAI-based (medium-confidence)	*Podoviridae*		

UViG	VirSorter2 score	Predicted genome type	Provirus	Completeness quality	Completeness (%)	Assembly quality	Completeness approach	Family	Host phylum	Host species
TM04_BL_k141_78047	1	dsDNAphage	No	High-quality	100	High-quality draft genome	AAI-based (high-confidence)		*Firmicutes*	*Coprococcus comes*
TM07_BL_k141_194303	1	dsDNAphage	No	Complete	100	High-quality draft genome	DTR (high-confidence)	*Microviridae*		
TF29_12wk_k141_82220	0.993	dsDNAphage	Yes	Complete	100	High-quality draft genome	DTR (high-confidence)		*Firmicutes*	*Coprococcus comes*
TF14_BL_k141_133814	1	dsDNAphage	No	High-quality	90.57	High-quality draft genome	AAI-based (high-confidence)			
TF21_26wk_k141_127106	0.62	ssDNA	Yes	High-quality	100	High-quality draft genome	HMM-based (lower-bound)			
TF11_6wk_k141_189848	1	dsDNAphage	No	Complete	100	High-quality draft genome	DTR (high-confidence)			
TF23_BL_k141_43771	0.947	dsDNAphage	No	High-quality	100	High-quality draft genome	HMM-based (lower-bound)			
TF26_6wk_k141_49227	0.993	dsDNAphage	No	Complete	100	High-quality draft genome	DTR (high-confidence)			
TF39_12wk_k141_83711	1	dsDNAphage	No	High-quality	100	High-quality draft genome	AAI-based (high-confidence)			
TF37_6wk_k141_147749	0.987	dsDNAphage	No	Complete	100	High-quality draft genome	DTR (high-confidence)		*Firmicutes*	*Blautia obeum*
TM30_12wk_k141_46568	0.993	dsDNAphage	No	High-quality	99.63	High-quality draft genome	AAI-based (high-confidence)		*Firmicutes*	*Ruminococcus bromii*
TF35_26wk_k141_132706	1	dsDNAphage	No	Complete	100	High-quality draft genome	DTR (high-confidence)			
TF41_12wk_k141_50150	0.987	dsDNAphage	No	High-quality	100	High-quality draft genome	AAI-based (high-confidence)		*Firmicutes*	*Roseburia intestinalis*
TM16_12wk_k141_20335	0.993	dsDNAphage	No	High-quality	100	High-quality draft genome	AAI-based (high-confidence)			
TM34_6wk_k141_5649	1	dsDNAphage	No	High-quality	90.23	High-quality draft genome	AAI-based (high-confidence)		*Actinobacteria*	*Bifidobacterium bifidum*
TM35_BL_k141_219044	0.98	dsDNAphage	No	High-quality	99.48	High-quality draft genome	AAI-based (high-confidence)			
TF34_26wk_k141_158075	0.993	dsDNAphage	No	Complete	100	High-quality draft genome	DTR (high-confidence)			
TM08_26wk_k141_23830	1	dsDNAphage	No	Complete	100	High-quality draft genome	DTR (high-confidence)		*Firmicutes*	*Staphylococcus aureus*
TF38_6wk_k141_54044	1	dsDNAphage	No	High-quality	100	High-quality draft genome	AAI-based (high-confidence)		*Firmicutes*	*Faecalibacterium prausnitzii*
TF31_6WK_k141_81915	0.973	dsDNAphage	No	High-quality	100	High-quality draft genome	AAI-based (high-confidence)		*Actinobacteria*	*Bifidobacterium longum*
TF26_12wk_k141_77616	0.993	dsDNAphage	No	High-quality	100	High-quality draft genome	AAI-based (high-confidence)			
TF40_BL_k141_3744	0.987	dsDNAphage	No	High-quality	100	High-quality draft genome	AAI-based (high-confidence)			
TF35_6wk_k141_186761	1	dsDNAphage	No	High-quality	90.84	High-quality draft genome	AAI-based (high-confidence)	*Siphoviridae*		
TM14_12wk_k141_87780	1	dsDNAphage	No	High-quality	100	High-quality draft genome	AAI-based (high-confidence)		*Actinobacteria*	*Bifidobacterium bifidum*
DF14_D7_k141_140558	0.993	dsDNAphage	No	High-quality	100	High-quality draft genome	AAI-based (high-confidence)			
DM12_D6_k141_5516	0.993	dsDNAphage	No	High-quality	95.25	High-quality draft genome	AAI-based (high-confidence)			
TF30_12wk_k141_27549	0.987	dsDNAphage	No	High-quality	99.83	High-quality draft genome	AAI-based (high-confidence)	*Siphoviridae*	*Firmicutes*	*Streptococcus pyogenes*
TF23_BL_k141_62365	1	dsDNAphage	No	High-quality	100	High-quality draft genome	AAI-based (high-confidence)			
DF16a_D4_k141_25439	0.993	dsDNAphage	No	High-quality	100	High-quality draft genome	AAI-based (high-confidence)			
TM12_12wk_k141_110910	1	dsDNAphage	No	Complete	100	High-quality draft genome	DTR (high-confidence)		*Firmicutes*	*Ruminococcus bromii*
DM03_D6_k141_19138	1	dsDNAphage	No	High-quality	98.95	High-quality draft genome	AAI-based (high-confidence)	*Siphoviridae*		
TM18_BL_k141_52293	0.993	dsDNAphage	No	High-quality	92.07	High-quality draft genome	AAI-based (high-confidence)			
TF03_26wk_k141_128142	1	dsDNAphage	No	High-quality	92.76	High-quality draft genome	AAI-based (high-confidence)	*Myoviridae*	*Firmicutes*	*Roseburia intestinalis*
TF44_6wk_k141_29164	0.993	dsDNAphage	No	Complete	100	High-quality draft genome	DTR (high-confidence)		*Firmicutes*	*Clostridioides difficile*
TF05_12wk_k141_18694	0.993	dsDNAphage	Yes	High-quality	96.21	High-quality draft genome	AAI-based (high-confidence)	*Podoviridae*		
TF15_BL_k141_39114	1	dsDNAphage	Yes	High-quality	98.09	High-quality draft genome	AAI-based (medium-confidence)			
DM07_D1_k141_15656	0.993	dsDNAphage	No	High-quality	100	High-quality draft genome	AAI-based (high-confidence)			
TM17_6WK_k141_106150	0.993	dsDNAphage	No	High-quality	100	High-quality draft genome	AAI-based (high-confidence)		*Firmicutes*	*Faecalibacterium prausnitzii*
TM22_26wk_k141_94127	1	dsDNAphage	No	High-quality	100	High-quality draft genome	AAI-based (high-confidence)			
TF46_12wk_k141_116711	0.88	ssDNA	No	High-quality	100	High-quality draft genome	HMM-based (lower-bound)			
DM08_D4_k141_66400	1	dsDNAphage	No	High-quality	100	High-quality draft genome	AAI-based (high-confidence)			
TF34_BL_k141_27748	0.993	dsDNAphage	No	High-quality	100	High-quality draft genome	AAI-based (high-confidence)			
TF03_12wk_k141_89668	1	dsDNAphage	No	High-quality	100	High-quality draft genome	AAI-based (high-confidence)		*Firmicutes*	*Roseburia intestinalis*
TM19_BL_k141_44432	0.993	dsDNAphage	No	High-quality	100	High-quality draft genome	AAI-based (medium-confidence)			
TF33_BL_k141_155278	1	dsDNAphage	No	Complete	100	High-quality draft genome	DTR (high-confidence)	*Crass_like*		
TF41_BL_k141_184976	0.967	dsDNAphage	No	High-quality	100	High-quality draft genome	AAI-based (high-confidence)			
TF12_6wk_k141_15275	1	dsDNAphage	No	Complete	100	High-quality draft genome	DTR (high-confidence)		*Firmicutes*	*Staphylococcus aureus*
DF16_D3_k141_296681	1	dsDNAphage	No	High-quality	94.62	High-quality draft genome	AAI-based (high-confidence)			
TF50_BL_k141_118405	0.993	dsDNAphage	No	Complete	100	High-quality draft genome	DTR (high-confidence)			
DF16_D7_k141_195504	0.993	dsDNAphage	No	Complete	100	High-quality draft genome	DTR (high-confidence)			
TM27_BL_k141_150840	1	dsDNAphage	No	High-quality	99.17	High-quality draft genome	AAI-based (high-confidence)			
TF33_6wk_k141_21460	1	dsDNAphage	No	Complete	100	High-quality draft genome	DTR (high-confidence)	*Crass_like*		
TF41_12wk_k141_54461	1	dsDNAphage	No	High-quality	100	High-quality draft genome	AAI-based (medium-confidence)	*Podoviridae*		
TF30_6wk_k141_47348	1	dsDNAphage	Yes	High-quality	100	High-quality draft genome	AAI-based (high-confidence)			
TM13_12wk_k141_122826	0.993	dsDNAphage	No	High-quality	100	High-quality draft genome	AAI-based (high-confidence)			
TM03_26wk_k141_18474	1	dsDNAphage	No	Complete	100	High-quality draft genome	DTR (high-confidence)	*Microviridae*	*Bacteroidetes*	*Prevotella bivia*
TM03_26wk_k141_37683	0.973	dsDNAphage	No	High-quality	100	High-quality draft genome	AAI-based (high-confidence)		*Firmicutes*	*Tyzzerella nexilis*
TF05_6wk_k141_48120	1	dsDNAphage	No	High-quality	100	High-quality draft genome	AAI-based (high-confidence)		*Firmicutes*	*Blautia hansenii*
TM18_26wk_k141_82553	1	dsDNAphage	No	High-quality	94.34	High-quality draft genome	AAI-based (high-confidence)		*Firmicutes*	*Eubacterium ventriosum*
TM24_12wk_k141_47367	0.993	dsDNAphage	No	High-quality	100	High-quality draft genome	AAI-based (high-confidence)			
TF23_6wk_k141_139786	1	dsDNAphage	No	High-quality	100	High-quality draft genome	AAI-based (high-confidence)			
TF16_BL_k141_142243	0.987	dsDNAphage	No	High-quality	98.83	High-quality draft genome	AAI-based (high-confidence)			
DF12_D7_k141_66552	0.993	dsDNAphage	No	High-quality	96.88	High-quality draft genome	AAI-based (high-confidence)	*Siphoviridae*		
TF37_BL_k141_157812	1	dsDNAphage	No	High-quality	94.24	High-quality draft genome	AAI-based (high-confidence)			
TM01_26wk_k141_150163	0.993	dsDNAphage	No	High-quality	100	High-quality draft genome	AAI-based (high-confidence)		*Firmicutes*	*Enterococcus faecalis*
TF08_BL_k141_76711	1	dsDNAphage	No	High-quality	95.89	High-quality draft genome	AAI-based (high-confidence)	*Siphoviridae*		
DM08_D5_k141_146857	0.993	ssDNA	No	High-quality	100	High-quality draft genome	HMM-based (lower-bound)			
TF32_12wk_k141_27758	0.987	dsDNAphage	No	High-quality	96.92	High-quality draft genome	AAI-based (high-confidence)		*Firmicutes*	*Tyzzerella nexilis*
DM07_D1_k141_17355	1	dsDNAphage	Yes	High-quality	91.87	High-quality draft genome	AAI-based (high-confidence)			
TF05_26wk_k141_80647	1	dsDNAphage	No	High-quality	91.04	High-quality draft genome	AAI-based (high-confidence)		*Firmicutes*	*Ruminococcus bromii*
TF40_6wk_k141_91504	0.987	dsDNAphage	No	High-quality	100	High-quality draft genome	AAI-based (high-confidence)		*Firmicutes*	*Faecalibacterium prausnitzii*
TF11_26wk_k141_113147	0.973	dsDNAphage	No	High-quality	100	High-quality draft genome	AAI-based (high-confidence)		*Firmicutes*	*Faecalibacterium prausnitzii*
TM39_BL_k141_39466	0.993	dsDNAphage	No	High-quality	94.67	High-quality draft genome	AAI-based (high-confidence)			
TM24_12wk_k141_83482	0.993	dsDNAphage	No	High-quality	100	High-quality draft genome	AAI-based (high-confidence)		*Firmicutes*	*Megamonas hypermegale*
TF41_12wk_k141_158414	0.993	dsDNAphage	No	High-quality	100	High-quality draft genome	AAI-based (high-confidence)	*Siphoviridae*		
TM24_12wk_k141_171086	0.993	dsDNAphage	No	Complete	100	High-quality draft genome	ITR (medium-confidence)		*Actinobacteria*	*Bifidobacterium adolescentis*
TM03_26wk_k141_6498	0.987	dsDNAphage	No	Complete	100	High-quality draft genome	DTR (high-confidence)	*Siphoviridae*	*Firmicutes*	*Tyzzerella nexilis*
TF35_26wk_k141_136493	0.993	dsDNAphage	No	High-quality	100	High-quality draft genome	AAI-based (high-confidence)		*Firmicutes*	*Staphylococcus aureus*
TM03_6wk_k141_2150	0.993	dsDNAphage	No	Complete	100	High-quality draft genome	DTR (high-confidence)	*Crass_like*	*Bacteroidetes*	*Parabacteroides distasonis*
TM25_26wk_k141_26896	1	dsDNAphage	No	High-quality	100	High-quality draft genome	AAI-based (high-confidence)			
TF35_26wk_k141_2813	0.993	dsDNAphage	No	High-quality	99.21	High-quality draft genome	AAI-based (high-confidence)		*Firmicutes*	*Dorea longicatena*
TF34_BL_k141_45326	0.993	dsDNAphage	No	Complete	100	High-quality draft genome	DTR (high-confidence)	*Crass_like*		
TF40_6wk_k141_21761	0.987	dsDNAphage	No	High-quality	100	High-quality draft genome	AAI-based (high-confidence)	*Microviridae*		
TF37_6wk_k141_143572	0.993	ssDNA	No	High-quality	100	High-quality draft genome	AAI-based (medium-confidence)			
TM14_26wk_k141_31235	0.987	dsDNAphage	No	High-quality	100	High-quality draft genome	AAI-based (high-confidence)		*Firmicutes*	*Roseburia intestinalis*
TF30_BL_k141_46259	1	dsDNAphage	No	High-quality	98.91	High-quality draft genome	AAI-based (high-confidence)		*Firmicutes*	*Dorea formicigenerans*
TF12_BL_k141_163785	0.987	dsDNAphage	No	High-quality	98.01	High-quality draft genome	AAI-based (medium-confidence)			
TM12_BL_k141_27026	0.66	dsDNAphage	No	High-quality	100	High-quality draft genome	HMM-based (lower-bound)			
TM37_BL_k141_114302	1	dsDNAphage	No	High-quality	100	High-quality draft genome	AAI-based (high-confidence)	*Myoviridae*	*Firmicutes*	*Roseburia inulinivorans*
TM24_6wk_k141_203648	1	dsDNAphage	No	High-quality	95.17	High-quality draft genome	AAI-based (high-confidence)		*Firmicutes*	*Anaerostipes hadrus*
TM09_26wk_k141_59038	0.973	dsDNAphage	No	High-quality	97.23	High-quality draft genome	AAI-based (high-confidence)		*Actinobacteria*	*Bifidobacterium adolescentis*
TM30_26wk_k141_24714	0.993	dsDNAphage	No	High-quality	95.33	High-quality draft genome	AAI-based (high-confidence)	*Siphoviridae*	*Firmicutes*	*Ruminococcus bromii*
TM05_6wk_k141_233898	1	dsDNAphage	No	Complete	100	High-quality draft genome	DTR (high-confidence)			
TF01_BL_k141_2359	0.973	ssDNA	No	High-quality	100	High-quality draft genome	HMM-based (lower-bound)			
TF15_BL_k141_95229	1	dsDNAphage	No	High-quality	100	High-quality draft genome	AAI-based (high-confidence)	*Myoviridae*		
TF16_12wk_k141_4005	0.967	dsDNAphage	No	High-quality	93.32	High-quality draft genome	AAI-based (high-confidence)		*Firmicutes*	*Faecalibacterium prausnitzii*
TF25_26wk_k141_24915	0.993	dsDNAphage	No	High-quality	100	High-quality draft genome	AAI-based (high-confidence)			
TM39_BL_k141_174763	0.987	dsDNAphage	No	Complete	100	High-quality draft genome	DTR (high-confidence)			
TF37_6wk_k141_71139	0.8	dsDNAphage	No	High-quality	100	High-quality draft genome	AAI-based (medium-confidence)			
TF44_6wk_k141_48081	1	dsDNAphage	No	High-quality	91.86	High-quality draft genome	AAI-based (high-confidence)		*Firmicutes*	*Coprococcus comes*
DM12_D5_k141_147447	0.967	dsDNAphage	No	Complete	100	High-quality draft genome	DTR (high-confidence)			
TF34_6wk_k141_261981	0.993	dsDNAphage	No	Complete	100	High-quality draft genome	DTR (high-confidence)		*Proteobacteria*	*Oxalobacter formigenes*
TF43_26wk_k141_111451	0.993	dsDNAphage	No	High-quality	100	High-quality draft genome	AAI-based (high-confidence)			
TF17_26wk_k141_81993	1	dsDNAphage	No	High-quality	97.91	High-quality draft genome	AAI-based (high-confidence)		*Firmicutes*	*Tyzzerella nexilis*
TM13_12wk_k141_122499	0.987	dsDNAphage	No	High-quality	97.91	High-quality draft genome	AAI-based (high-confidence)			

UViG	VirSorter2 score	Predicted genome type	Provirus	Completeness quality	Completeness (%)	Assembly quality	Completeness approach	Family	Host phylum	Host species
TF51_BL_k141_4182	0.993	dsDNAphage	No	High-quality	98.98	High-quality draft genome	AAI-based (high-confidence)			
TF38_BL_k141_25887	1	dsDNAphage	No	High-quality	100	High-quality draft genome	AAI-based (high-confidence)			
TF27_BL_k141_97127	1	dsDNAphage	No	High-quality	100	High-quality draft genome	AAI-based (high-confidence)			
DF14_D8_k141_133585	0.993	dsDNAphage	No	Complete	100	High-quality draft genome	DTR (medium-confidence)	*Myoviridae*		
TM27_26wk_k141_78970	1	dsDNAphage	No	High-quality	100	High-quality draft genome	AAI-based (high-confidence)		*Firmicutes*	*Ruminococcus bromii*
TM18_12wk_k141_53544	1	dsDNAphage	No	High-quality	97.31	High-quality draft genome	AAI-based (high-confidence)	*Myoviridae*		
TF34_6wk_k141_187095	1	dsDNAphage	Yes	High-quality	98.68	High-quality draft genome	AAI-based (high-confidence)			
TF10_6wk_k141_109108	1	dsDNAphage	No	High-quality	97.87	High-quality draft genome	AAI-based (high-confidence)	*Microviridae*	*Bacteroidetes*	*Prevotella disiens*
DF14_D4_k141_111545	1	dsDNAphage	No	High-quality	100	High-quality draft genome	AAI-based (high-confidence)			
TM25_26wk_k141_47442	1	dsDNAphage	No	High-quality	96.98	High-quality draft genome	AAI-based (high-confidence)		*Firmicutes*	*Blautia hansenii*
TM34_BL_k141_155966	1	dsDNAphage	No	High-quality	98.96	High-quality draft genome	AAI-based (high-confidence)			
TF50_BL_k141_127135	0.987	dsDNAphage	No	High-quality	100	High-quality draft genome	AAI-based (high-confidence)			
DF16_D3_k141_162318	0.947	dsDNAphage	No	High-quality	100	High-quality draft genome	AAI-based (high-confidence)			
TF20_BL_k141_157662	1	dsDNAphage	No	High-quality	91.55	High-quality draft genome	AAI-based (high-confidence)			
TF01_12wk_k141_9254	0.993	dsDNAphage	No	High-quality	100	High-quality draft genome	AAI-based (high-confidence)		*Bacteroidetes*	*Bacteroides vulgatus*
TM27_6wk_k141_20975	0.987	dsDNAphage	No	High-quality	100	High-quality draft genome	AAI-based (medium-confidence)		*Firmicutes*	*Ruminococcus bromii*
TM16_12wk_k141_25432[illegible]	0.993	dsDNAphage	No	Complete	100	High-quality draft genome	DTR (high-confidence)		*Firmicutes*	*Ruminococcus bromii*
TF01_26wk_k141_102933	0.993	dsDNAphage	No	High-quality	99.06	High-quality draft genome	AAI-based (high-confidence)		*Bacteroidetes*	*Prevotella timonensis*
DM12_D3_k141_25161	0.987	dsDNAphage	Yes	High-quality	100	High-quality draft genome	AAI-based (high-confidence)			
TF32_12wk_k141_75936	1	dsDNAphage	No	High-quality	100	High-quality draft genome	AAI-based (high-confidence)			
TF13_BL_k141_121504	0.993	dsDNAphage	No	High-quality	100	High-quality draft genome	AAI-based (high-confidence)	*Myoviridae*		
TF25_BL_k141_91908	1	dsDNAphage	No	High-quality	100	High-quality draft genome	AAI-based (high-confidence)	*Microviridae*		
TM10_BL_k141_11520	0.987	dsDNAphage	No	High-quality	100	High-quality draft genome	AAI-based (high-confidence)	*Myoviridae*		
TF20_26wk_k141_144503	0.927	dsDNAphage	No	High-quality	100	High-quality draft genome	AAI-based (high-confidence)		*Firmicutes*	*Ruminococcus champanellensis*
TM24_12wk_k141_14735	0.987	dsDNAphage	No	High-quality	100	High-quality draft genome	AAI-based (high-confidence)			
TF35_6wk_k141_38885	0.993	dsDNAphage	No	High-quality	100	High-quality draft genome	AAI-based (high-confidence)		*Firmicutes*	*Ruminococcus lactaris*
DF17_D8_k141_98	0.967	dsDNAphage	Yes	High-quality	100	High-quality draft genome	AAI-based (medium-confidence)	*Microviridae*		
TM20_26wk_k141_13428[illegible]	1	dsDNAphage	No	High-quality	92.67	High-quality draft genome	AAI-based (high-confidence)			
TF33_BL_k141_89761	1	dsDNAphage	No	Complete	100	High-quality draft genome	DTR (high-confidence)			
TF11_26wk_k141_46397	0.993	dsDNAphage	Yes	High-quality	100	High-quality draft genome	AAI-based (medium-confidence)			
TF44_BL_k141_11163	1	dsDNAphage	No	High-quality	100	High-quality draft genome	AAI-based (high-confidence)	*Crass_like*		
TM21_12wk_k141_71824	0.993	dsDNAphage	No	High-quality	100	High-quality draft genome	AAI-based (high-confidence)			
TM10_26wk_k141_89564	0.987	dsDNAphage	No	High-quality	100	High-quality draft genome	AAI-based (high-confidence)		*Firmicutes*	*Ruminococcus champanellensis*
TF01_26wk_k141_71959	0.567	dsDNAphage	No	High-quality	100	High-quality draft genome	AAI-based (medium-confidence)			
TF40_BL_k141_104397	0.927	dsDNAphage	No	High-quality	90.16	High-quality draft genome	AAI-based (high-confidence)			
TM13_26wk_k141_18002[illegible]	1	dsDNAphage	No	High-quality	99.82	High-quality draft genome	AAI-based (high-confidence)			
DM07_D4_k141_106094	0.987	dsDNAphage	No	High-quality	94.55	High-quality draft genome	AAI-based (high-confidence)			
DM08_D1_k141_142557	0.993	dsDNAphage	No	High-quality	100	High-quality draft genome	AAI-based (high-confidence)			
DF17_D6_k141_113288	0.987	dsDNAphage	No	High-quality	100	High-quality draft genome	AAI-based (high-confidence)			
TF01_26wk_k141_20546	0.987	dsDNAphage	Yes	High-quality	100	High-quality draft genome	AAI-based (high-confidence)	*Microviridae*		
TF49_12wk_k141_23422	0.9	ssDNA	No	High-quality	100	High-quality draft genome	HMM-based (lower-bound)			
TM07_12wk_k141_45722	1	dsDNAphage	No	High-quality	100	High-quality draft genome	AAI-based (high-confidence)			
TM12_26wk_k141_13140[illegible]	0.987	dsDNAphage	No	High-quality	100	High-quality draft genome	AAI-based (high-confidence)	*Myoviridae*	*Firmicutes*	*Roseburia intestinalis*
TF07_12wk_k141_30645	0.94	ssDNA	No	High-quality	100	High-quality draft genome	HMM-based (lower-bound)			
TM13_BL_k141_92789	1	dsDNAphage	No	Complete	100	High-quality draft genome	DTR (high-confidence)	*Crass_like*		
TM09_26wk_k141_20519	1	dsDNAphage	No	High-quality	100	High-quality draft genome	AAI-based (high-confidence)		*Firmicutes*	*Coprococcus comes*
TM04_26wk_k141_11233[illegible]	1	dsDNAphage	No	High-quality	100	High-quality draft genome	AAI-based (high-confidence)		*Actinobacteria*	*Bifidobacterium adolescentis*
TF45_12wk_k141_88106	0.987	dsDNAphage	No	Complete	100	High-quality draft genome	DTR (high-confidence)		*Firmicutes*	*Roseburia intestinalis*
TF21_26wk_k141_207047	0.993	dsDNAphage	No	High-quality	100	High-quality draft genome	AAI-based (high-confidence)	*Myoviridae*	*Firmicutes*	*Roseburia inulinivorans*
TF25_12wk_k141_69517	1	dsDNAphage	Yes	High-quality	91.66	High-quality draft genome	AAI-based (high-confidence)		*Firmicutes*	*Faecalibacterium prausnitzii*
DF16a_D4_k141_170832	1	dsDNAphage	No	High-quality	100	High-quality draft genome	AAI-based (high-confidence)			
TM23_12wk_k141_9581	0.993	dsDNAphage	No	High-quality	100	High-quality draft genome	AAI-based (high-confidence)		*Bacteroidetes*	*Bacteroides uniformis*
TF05_BL_k141_93097	0.993	dsDNAphage	No	High-quality	100	High-quality draft genome	AAI-based (high-confidence)			
TM08_12wk_k141_18578[illegible]	1	dsDNAphage	No	High-quality	100	High-quality draft genome	AAI-based (high-confidence)	*Myoviridae*		
TF09_BL_k141_245662	1	dsDNAphage	No	High-quality	100	High-quality draft genome	AAI-based (high-confidence)			
TM22_6wk_k141_66523	1	dsDNAphage	No	Complete	100	High-quality draft genome	DTR (high-confidence)	*Crass_like*	*Bacteroidetes*	*Parabacteroides johnsonii*
TF34_12wk_k141_168414	0.993	dsDNAphage	No	Complete	100	High-quality draft genome	DTR (high-confidence)		*Firmicutes*	*Faecalibacterium prausnitzii*
TF41_BL_k141_181721	1	dsDNAphage	No	Complete	100	High-quality draft genome	DTR (high-confidence)			
TM39_6wk_k141_94819	0.993	dsDNAphage	No	High-quality	100	High-quality draft genome	AAI-based (high-confidence)		*Firmicutes*	*Coprococcus comes*
TM23_6wk_k141_109742	1	dsDNAphage	No	Complete	100	High-quality draft genome	DTR (high-confidence)	*Microviridae*		
TF41_BL_k141_149250	0.993	dsDNAphage	No	Complete	100	High-quality draft genome	DTR (high-confidence)			
TM08_12wk_k141_14577[illegible]	0.993	dsDNAphage	No	High-quality	100	High-quality draft genome	AAI-based (high-confidence)		*Bacteroidetes*	*Bacteroides caccae*
TF32_12wk_k141_46327	1	dsDNAphage	No	High-quality	100	High-quality draft genome	AAI-based (high-confidence)		*Firmicutes*	*Ruminococcus bromii*
TF26_26wk_k141_101416	0.993	dsDNAphage	Yes	High-quality	95.64	High-quality draft genome	AAI-based (high-confidence)		*Actinobacteria*	*Bifidobacterium adolescentis*
TF32_BL_k141_103279	0.947	dsDNAphage	No	High-quality	100	High-quality draft genome	AAI-based (high-confidence)			
TF30_12wk_k141_149558	0.967	dsDNAphage	No	High-quality	90.85	High-quality draft genome	AAI-based (high-confidence)			
TM04_6wk_k141_53940	1	dsDNAphage	No	High-quality	91.76	High-quality draft genome	AAI-based (medium-confidence)	*Podoviridae*		
TF25_26wk_k141_130479	0.887	dsDNAphage	No	High-quality	100	High-quality draft genome	HMM-based (lower-bound)			
TF51_26wk_k141_34861	1	dsDNAphage	No	Complete	100	High-quality draft genome	DTR (high-confidence)			
TM35_BL_k141_45190	0.96	ssDNA	No	High-quality	100	High-quality draft genome	HMM-based (lower-bound)			
DF14_D5_k141_71033	0.993	dsDNAphage	No	High-quality	100	High-quality draft genome	AAI-based (high-confidence)			
TF19_BL_k141_202732	0.993	dsDNAphage	No	Complete	100	High-quality draft genome	DTR (high-confidence)			
TF31_12wk_k141_143969	1	dsDNAphage	No	High-quality	100	High-quality draft genome	AAI-based (high-confidence)		*Firmicutes*	*Ruminococcus bromii*
TM13_12wk_k141_231955	0.993	dsDNAphage	No	Complete	100	High-quality draft genome	DTR (high-confidence)			
TF39_BL_k141_6810	0.987	dsDNAphage	No	High-quality	92.29	High-quality draft genome	AAI-based (high-confidence)			
TF17_BL_k141_70197	1	dsDNAphage	No	High-quality	97.69	High-quality draft genome	AAI-based (high-confidence)	*Myoviridae*		
TM03_12wk_k141_37259	1	dsDNAphage	No	High-quality	100	High-quality draft genome	AAI-based (high-confidence)	*Myoviridae*		
TF31_26wk_k141_121452	1	dsDNAphage	Yes	High-quality	100	High-quality draft genome	AAI-based (high-confidence)		*Firmicutes*	*Faecalibacterium prausnitzii*
TF37_12wk_k141_87188	1	dsDNAphage	No	High-quality	98	High-quality draft genome	AAI-based (high-confidence)	*Podoviridae*	*Proteobacteria*	*Escherichia coli*
TF24_26wk_k141_92860	0.973	dsDNAphage	Yes	High-quality	100	High-quality draft genome	AAI-based (high-confidence)			
TF11_6wk_k141_134814	0.98	dsDNAphage	No	High-quality	100	High-quality draft genome	AAI-based (high-confidence)			
TF03_26wk_k141_2242	1	dsDNAphage	No	Complete	100	High-quality draft genome	DTR (high-confidence)		*Firmicutes*	*Staphylococcus aureus*
TF23_6wk_k141_124286	1	dsDNAphage	No	High-quality	100	High-quality draft genome	AAI-based (high-confidence)	*Siphoviridae*	*Firmicutes*	*Faecalibacterium prausnitzii*
TF51_12wk_k141_15106	1	dsDNAphage	No	High-quality	100	High-quality draft genome	AAI-based (high-confidence)		*Firmicutes*	*Tyzzerella nexilis*
TM32_6WK_k141_94902	0.987	dsDNAphage	No	High-quality	100	High-quality draft genome	AAI-based (high-confidence)	*Siphoviridae*	*Firmicutes*	*Lactobacillus ruminis*
TM09_BL_k141_91984	0.993	dsDNAphage	No	Complete	100	High-quality draft genome	DTR (medium-confidence)			
TF27_BL_k141_24768	0.98	dsDNAphage	No	High-quality	98.68	High-quality draft genome	AAI-based (high-confidence)			
TF38_12wk_k141_37896	0.993	dsDNAphage	No	Complete	100	High-quality draft genome	DTR (high-confidence)		*Firmicutes*	*Faecalibacterium prausnitzii*
TM11_BL_k141_76349	1	dsDNAphage	No	High-quality	100	High-quality draft genome	AAI-based (high-confidence)			
TF35_12wk_k141_96622	0.947	dsDNAphage	No	High-quality	93.17	High-quality draft genome	AAI-based (high-confidence)		*Firmicutes*	*Faecalibacterium prausnitzii*
TM13_6wk_k141_165640	0.993	dsDNAphage	No	Complete	100	High-quality draft genome	DTR (high-confidence)	*Microviridae*		
TM27_12wk_k141_10366[illegible]	1	dsDNAphage	No	Complete	100	High-quality draft genome	DTR (high-confidence)	*Siphoviridae*		
DF14_D3_k141_122457	1	dsDNAphage	No	Complete	100	High-quality draft genome	DTR (high-confidence)	*Myoviridae*		
TF33_6wk_k141_75749	0.933	dsDNAphage	No	High-quality	100	High-quality draft genome	AAI-based (high-confidence)			
TF47_26wk_k141_32256	0.993	dsDNAphage	No	High-quality	100	High-quality draft genome	AAI-based (high-confidence)	*Microviridae*		
TF45_26wk_k141_4414	1	dsDNAphage	No	High-quality	100	High-quality draft genome	AAI-based (high-confidence)		*Bacteroidetes*	*Bacteroides ovatus*
TF50_BL_k141_9558	0.993	dsDNAphage	No	High-quality	100	High-quality draft genome	AAI-based (high-confidence)			
TF20_BL_k141_53180	0.94	ssDNA	No	High-quality	100	High-quality draft genome	HMM-based (lower-bound)	*Circoviridae*		
TF09_BL_k141_41779	0.94	ssDNA	No	High-quality	100	High-quality draft genome	AAI-based (medium-confidence)			
TM33_6wk_k141_2615	0.993	dsDNAphage	No	High-quality	100	High-quality draft genome	AAI-based (high-confidence)		*Firmicutes*	*Faecalibacterium prausnitzii*
TF13_BL_k141_164829	0.98	dsDNAphage	Yes	High-quality	99.21	High-quality draft genome	AAI-based (high-confidence)			
TM35_BL_k141_126246	0.98	dsDNAphage	No	High-quality	100	High-quality draft genome	AAI-based (high-confidence)			
TF02_6wk_k141_20695	0.94	dsDNAphage	No	High-quality	93.01	High-quality draft genome	AAI-based (high-confidence)		*Firmicutes*	*Faecalibacterium prausnitzii*
TF01_6wk_k141_52681	0.987	dsDNAphage	Yes	High-quality	93.42	High-quality draft genome	AAI-based (high-confidence)			
TF10_6wk_k141_8115	0.993	dsDNAphage	Yes	High-quality	94.16	High-quality draft genome	AAI-based (high-confidence)		*Firmicutes*	*Faecalibacterium prausnitzii*

UViG	VirSorter2 score	Predicted genome type	Provirus	Completeness quality	Completeness (%)	Assembly quality	Completeness approach	Family	Host phylum	Host species
DM12_D6_k141_145981	1	dsDNAphage	No	High-quality	93.68	High-quality draft genome	AAI-based (high-confidence)			
TF16_12wk_k141_100185	1	dsDNAphage	No	High-quality	97.18	High-quality draft genome	AAI-based (high-confidence)		*Bacteroidetes*	*Prevotella copri*
TF51_BL_k141_62319	0.973	dsDNAphage	No	High-quality	100	High-quality draft genome	AAI-based (high-confidence)			
DM08_D4_k141_226715	1	dsDNAphage	No	Complete	100	High-quality draft genome	DTR (high-confidence)			
DM07_D2_k141_106996	1	dsDNAphage	No	High-quality	94.1	High-quality draft genome	AAI-based (high-confidence)	*Siphoviridae*		
TF48_6wk_k141_134178	0.987	dsDNAphage	No	High-quality	100	High-quality draft genome	AAI-based (high-confidence)			
TM23_BL_k141_127508	1	dsDNAphage	No	Complete	100	High-quality draft genome	DTR (high-confidence)	*Microviridae*		
TF01_12wk_k141_46864	0.833	dsDNAphage	No	High-quality	98.01	High-quality draft genome	AAI-based (medium-confidence)			
TF47_26wk_k141_215682	1	dsDNAphage	No	High-quality	100	High-quality draft genome	AAI-based (high-confidence)			
TF39_12wk_k141_6572	0.673	dsDNAphage	No	High-quality	100	High-quality draft genome	AAI-based (high-confidence)			
TF25_12wk_k141_19951	0.9	ssDNA	No	High-quality	100	High-quality draft genome	HMM-based (lower-bound)			
TF03_BL_k141_15633	0.993	dsDNAphage	No	High-quality	93.94	High-quality draft genome	AAI-based (medium-confidence)	*Myoviridae*		
TF17_12wk_k141_47652	0.98	dsDNAphage	No	Complete	100	High-quality draft genome	DTR (high-confidence)			
TF41_26wk_k141_114132	0.933	ssDNA	No	High-quality	100	High-quality draft genome	AAI-based (medium-confidence)			
DM03_D3_k141_52828	0.987	dsDNAphage	Yes	High-quality	93.4	High-quality draft genome	AAI-based (high-confidence)	*Siphoviridae*		
TF04_BL_k141_130514	1	dsDNAphage	No	High-quality	95.99	High-quality draft genome	AAI-based (high-confidence)	*Podoviridae*		
TM18_BL_k141_81350	0.987	dsDNAphage	No	High-quality	100	High-quality draft genome	AAI-based (high-confidence)			
TF23_6wk_k141_128569	1	dsDNAphage	No	Complete	100	High-quality draft genome	DTR (high-confidence)	*Siphoviridae*		
TF29_BL_k141_12258	0.993	dsDNAphage	No	High-quality	100	High-quality draft genome	AAI-based (high-confidence)			
TF47_26wk_k141_214781	0.993	dsDNAphage	No	High-quality	100	High-quality draft genome	AAI-based (high-confidence)		*Actinobacteria*	*Bifidobacterium adolescentis*
TF38_6wk_k141_122910	1	dsDNAphage	No	High-quality	98.2	High-quality draft genome	AAI-based (high-confidence)			
DM05_D1_k141_6144	0.987	dsDNAphage	Yes	High-quality	100	High-quality draft genome	AAI-based (high-confidence)			
TF20_BL_k141_197090	1	dsDNAphage	No	Complete	100	High-quality draft genome	DTR (high-confidence)			
TM14_BL_k141_174462	1	dsDNAphage	No	Complete	100	High-quality draft genome	DTR (high-confidence)		*Bacteroidetes*	*Bacteroides vulgatus*
TM27_6wk_k141_9396	0.967	dsDNAphage	No	High-quality	90.73	High-quality draft genome	AAI-based (high-confidence)	*Siphoviridae*	*Firmicutes*	*Faecalibacterium prausnitzii*
TF40_BL_k141_63536	0.993	dsDNAphage	No	High-quality	100	High-quality draft genome	AAI-based (high-confidence)			
DF17_D8_k141_63223	0.987	dsDNAphage	No	High-quality	96.96	High-quality draft genome	AAI-based (high-confidence)			
TF41_6wk_k141_160329	0.72	dsDNAphage	No	High-quality	100	High-quality draft genome	AAI-based (high-confidence)			
TM16_12wk_k141_254288	0.993	dsDNAphage	No	Complete	100	High-quality draft genome	DTR (high-confidence)		*Firmicutes*	*Roseburia intestinalis*
TF03_26wk_k141_185363	0.993	dsDNAphage	No	High-quality	100	High-quality draft genome	AAI-based (medium-confidence)			
TM37_BL_k141_41808	1	dsDNAphage	No	High-quality	100	High-quality draft genome	AAI-based (high-confidence)			
DF16_D3_k141_58498	0.987	dsDNAphage	No	High-quality	100	High-quality draft genome	AAI-based (high-confidence)			
TM36_12wk_k141_23814	0.933	dsDNAphage	No	High-quality	96.97	High-quality draft genome	AAI-based (medium-confidence)		*Bacteroidetes*	*Prevotella histicola*
TF47_26wk_k141_98701	1	dsDNAphage	No	High-quality	100	High-quality draft genome	AAI-based (high-confidence)			
TF03_BL_k141_88338	1	dsDNAphage	No	Complete	100	High-quality draft genome	DTR (high-confidence)	*Crass_like*		
TF40_6wk_k141_127126	0.987	dsDNAphage	No	High-quality	96.9	High-quality draft genome	AAI-based (medium-confidence)		*Bacteroidetes*	*Bacteroides uniformis*
DF14_D3_k141_85899	1	dsDNAphage	No	High-quality	100	High-quality draft genome	AAI-based (high-confidence)			
DM12_D4_k141_33828	1	dsDNAphage	No	High-quality	99.17	High-quality draft genome	AAI-based (high-confidence)			
TM24_26wk_k141_26287	0.907	dsDNAphage	No	High-quality	100	High-quality draft genome	AAI-based (high-confidence)			
TF42_26wk_k141_66774	0.993	dsDNAphage	No	High-quality	97.81	High-quality draft genome	AAI-based (high-confidence)	*Myoviridae*		
TF39_BL_k141_182776	0.993	dsDNAphage	No	Complete	100	High-quality draft genome	DTR (high-confidence)			
TM39_26wk_k141_55535	1	dsDNAphage	No	High-quality	100	High-quality draft genome	AAI-based (high-confidence)			
TF15_12wk_k141_55494	0.967	dsDNAphage	No	High-quality	100	High-quality draft genome	HMM-based (lower-bound)		*Firmicutes*	*Ruminococcus bromii*
TF36_6wk_k141_162450	1	dsDNAphage	No	High-quality	100	High-quality draft genome	AAI-based (high-confidence)	*Myoviridae*	*Firmicutes*	*Coprococcus catus*
TF03_12wk_k141_21995	0.973	dsDNAphage	No	High-quality	94.66	High-quality draft genome	AAI-based (medium-confidence)			
DF16_D5_k141_23413	1	dsDNAphage	No	High-quality	90.45	High-quality draft genome	AAI-based (medium-confidence)			
TF14_6wk_k141_110916	1	dsDNAphage	No	Complete	100	High-quality draft genome	DTR (high-confidence)	*Siphoviridae*	*Firmicutes*	*Staphylococcus aureus*
TM33_6wk_k141_120657	0.973	dsDNAphage	No	High-quality	100	High-quality draft genome	AAI-based (medium-confidence)		*Firmicutes*	*Ruminococcus bromii*
TM09_26wk_k141_4254	1	dsDNAphage	No	Complete	100	High-quality draft genome	DTR (high-confidence)		*Firmicutes*	*Staphylococcus aureus*
TF24_6WK_k141_14748	0.987	dsDNAphage	No	High-quality	94	High-quality draft genome	AAI-based (medium-confidence)		*Bacteroidetes*	*Prevotella denticola*
TM12_BL_k141_89394	0.98	dsDNAphage	No	Complete	100	High-quality draft genome	DTR (high-confidence)			
TF09_6WK_k141_56624	1	dsDNAphage	No	High-quality	97.02	High-quality draft genome	AAI-based (high-confidence)	*Crass_like*	*Bacteroidetes*	*Parabacteroides distasonis*
TF07_12wk_k141_54118	0.973	dsDNAphage	Yes	Complete	100	High-quality draft genome	Provirus (high-confidence)			
TF50_6wk_k141_140722	0.993	dsDNAphage	No	High-quality	91.44	High-quality draft genome	AAI-based (high-confidence)		*Actinobacteria*	*Bifidobacterium pseudocatenulatum*
TM16_12wk_k141_254323	1	dsDNAphage	No	Complete	100	High-quality draft genome	DTR (high-confidence)	*Microviridae*		
TM35_BL_k141_37764	1	dsDNAphage	No	High-quality	99.87	High-quality draft genome	AAI-based (high-confidence)			
TM01_26wk_k141_111926	0.987	dsDNAphage	No	High-quality	100	High-quality draft genome	AAI-based (high-confidence)		*Actinobacteria*	*Bifidobacterium adolescentis*
TM34_12wk_k141_177144	0.987	dsDNAphage	No	Complete	100	High-quality draft genome	DTR (high-confidence)		*Firmicutes*	*Coprococcus comes*
TF01_BL_k141_18041	1	dsDNAphage	No	Complete	100	High-quality draft genome	DTR (high-confidence)			
TM30_12wk_k141_1453	0.993	dsDNAphage	No	Complete	100	High-quality draft genome	DTR (high-confidence)		*Firmicutes*	*Streptococcus pyogenes*
TM06_12wk_k141_3662	0.987	dsDNAphage	No	High-quality	100	High-quality draft genome	AAI-based (high-confidence)			
TF01_6wk_k141_90254	0.993	dsDNAphage	No	High-quality	100	High-quality draft genome	AAI-based (high-confidence)			
DM12_D5_k141_127278	0.993	dsDNAphage	No	High-quality	97.71	High-quality draft genome	AAI-based (high-confidence)			
DF16_D2_k141_159604	0.993	dsDNAphage	No	High-quality	100	High-quality draft genome	AAI-based (high-confidence)			
TM39_26wk_k141_56750	0.993	dsDNAphage	No	Complete	100	High-quality draft genome	DTR (high-confidence)	*Crass_like*	*Bacteroidetes*	*Parabacteroides distasonis*
TF09_6WK_k141_63588	1	dsDNAphage	Yes	High-quality	97.28	High-quality draft genome	AAI-based (high-confidence)	*Myoviridae*		
DM03_D6_k141_36196	0.993	dsDNAphage	No	Complete	100	High-quality draft genome	DTR (high-confidence)			
TM39_12wk_k141_38545	0.993	dsDNAphage	No	High-quality	100	High-quality draft genome	AAI-based (high-confidence)			
TM14_BL_k141_106576	1	dsDNAphage	No	High-quality	99.99	High-quality draft genome	AAI-based (medium-confidence)			
TF50_6wk_k141_37733	0.993	dsDNAphage	No	High-quality	100	High-quality draft genome	AAI-based (high-confidence)	*Siphoviridae*		
TM05_26wk_k141_22040[illegible]	0.8	dsDNAphage	No	High-quality	98.65	High-quality draft genome	AAI-based (medium-confidence)			
TM39_6wk_k141_80309	0.953	dsDNAphage	No	High-quality	95.95	High-quality draft genome	AAI-based (high-confidence)		*Firmicutes*	*Ruminococcus champanellensis*
TF31_BL_k141_2337	0.967	dsDNAphage	No	High-quality	100	High-quality draft genome	AAI-based (high-confidence)			
TF07_6wk_k141_165759	0.907	ssDNA	No	High-quality	100	High-quality draft genome	AAI-based (high-confidence)			
TM02_12wk_k141_34573	1	dsDNAphage	No	High-quality	93.33	High-quality draft genome	AAI-based (high-confidence)		*Firmicutes*	*Faecalibacterium prausnitzii*
TM23_BL_k141_121447	0.993	dsDNAphage	No	High-quality	100	High-quality draft genome	AAI-based (high-confidence)			
TF19_6WK_k141_168875	0.973	dsDNAphage	Yes	High-quality	97.07	High-quality draft genome	AAI-based (high-confidence)		*Firmicutes*	*Faecalibacterium prausnitzii*
TM02_26wk_k141_29555	0.993	dsDNAphage	No	High-quality	100	High-quality draft genome	AAI-based (high-confidence)		*Firmicutes*	*Blautia obeum*
TM07_BL_k141_121955	0.993	dsDNAphage	No	High-quality	100	High-quality draft genome	AAI-based (high-confidence)			
DF16a_D4_k141_35454	1	dsDNAphage	No	High-quality	100	High-quality draft genome	AAI-based (high-confidence)			
TM01_26wk_k141_150957	0.613	dsDNAphage	No	Complete	100	High-quality draft genome	DTR (high-confidence)			
TM20_12wk_k141_86293	0.973	dsDNAphage	No	Complete	100	High-quality draft genome	DTR (high-confidence)			
TM22_6wk_k141_1255	0.94	dsDNAphage	No	High-quality	100	High-quality draft genome	AAI-based (high-confidence)			
TF32_BL_k141_7610	1	dsDNAphage	No	High-quality	100	High-quality draft genome	AAI-based (high-confidence)	*Siphoviridae*		
TF20_6WK_k141_160151	1	dsDNAphage	No	Complete	100	High-quality draft genome	ITR (high-confidence)	*Podoviridae*	*Actinobacteria*	*Bifidobacterium pseudocatenulatum*
TF32_12wk_k141_8461	1	dsDNAphage	No	High-quality	100	High-quality draft genome	AAI-based (high-confidence)		*Firmicutes*	*Anaerobutyricum hallii*
TF02_6wk_k141_270153	1	dsDNAphage	No	Complete	100	High-quality draft genome	DTR (high-confidence)	*Crass_like*	*Firmicutes*	*Staphylococcus aureus*
TF19_6WK_k141_62835	0.973	dsDNAphage	Yes	Complete	100	High-quality draft genome	Provirus (high-confidence)			
TM28_BL_k141_234449	0.987	dsDNAphage	No	Complete	100	High-quality draft genome	DTR (high-confidence)			
TF18_12wk_k141_46559	0.98	dsDNAphage	No	High-quality	100	High-quality draft genome	AAI-based (high-confidence)	*Siphoviridae*	*Firmicutes*	*Faecalibacterium prausnitzii*
TM19_BL_k141_26001	0.993	dsDNAphage	No	High-quality	90.62	High-quality draft genome	AAI-based (high-confidence)			
TM16_26wk_k141_113398	0.98	dsDNAphage	No	High-quality	100	High-quality draft genome	AAI-based (high-confidence)		*Bacteroidetes*	*Prevotella multiformis*
TF51_BL_k141_27449	1	dsDNAphage	No	High-quality	100	High-quality draft genome	AAI-based (high-confidence)			
TM28_6wk_k141_73645	1	dsDNAphage	No	High-quality	91.68	High-quality draft genome	AAI-based (high-confidence)			
DF14_D8_k141_31191	1	dsDNAphage	No	High-quality	99.52	High-quality draft genome	AAI-based (high-confidence)			
DF14_D8_k141_77022	1	dsDNAphage	No	High-quality	94.33	High-quality draft genome	AAI-based (medium-confidence)			
TM05_BL_k141_211466	1	dsDNAphage	No	High-quality	100	High-quality draft genome	AAI-based (medium-confidence)			
DM12_D3_k141_105979	0.993	dsDNAphage	No	High-quality	100	High-quality draft genome	AAI-based (high-confidence)			
TF08_6wk_k141_40443	0.993	dsDNAphage	Yes	High-quality	100	High-quality draft genome	AAI-based (high-confidence)			
TM03_BL_k141_68743	1	dsDNAphage	No	High-quality	100	High-quality draft genome	AAI-based (high-confidence)			
TM15_BL_k141_65363	0.873	dsDNAphage	No	High-quality	100	High-quality draft genome	AAI-based (high-confidence)			
TF45_BL_k141_23009	0.953	dsDNAphage	No	High-quality	100	High-quality draft genome	AAI-based (medium-confidence)			
TF27_6wk_k141_20475	1	dsDNAphage	No	Complete	100	High-quality draft genome	DTR (high-confidence)			
TF05_12wk_k141_99575	0.993	dsDNAphage	No	Complete	100	High-quality draft genome	DTR (high-confidence)		*Firmicutes*	*Faecalibacterium prausnitzii*
TM14_26wk_k141_12648[illegible]	1	dsDNAphage	No	Complete	100	High-quality draft genome	DTR (high-confidence)		*Firmicutes*	*Coprococcus comes*

UViG	VirSorter2 score	Predicted genome type	Provirus	Completeness quality	Completeness (%)	Assembly quality	Completeness approach	Family	Host phylum	Host species
TF35_BL_k141_68394	1	dsDNAphage	No	High-quality	91.64	High-quality draft genome	AAI-based (high-confidence)			
TF17_BL_k141_37790	1	dsDNAphage	No	High-quality	100	High-quality draft genome	AAI-based (high-confidence)	*Myoviridae*		
TM13_BL_k141_42765	1	dsDNAphage	No	High-quality	96.85	High-quality draft genome	AAI-based (medium-confidence)			
TM24_BL_k141_81302	1	dsDNAphage	No	High-quality	92.17	High-quality draft genome	AAI-based (high-confidence)			
TM05_26wk_k141_219781	1	dsDNAphage	No	High-quality	99.73	High-quality draft genome	AAI-based (high-confidence)		*Firmicutes*	*Faecalibacterium prausnitzii*
TM14_BL_k141_127016	0.993	dsDNAphage	No	Complete	100	High-quality draft genome	DTR (high-confidence)			
TF32_6wk_k141_35588	0.987	dsDNAphage	No	High-quality	99.12	High-quality draft genome	AAI-based (high-confidence)			
TM05_BL_k141_112731	0.993	dsDNAphage	No	High-quality	100	High-quality draft genome	AAI-based (medium-confidence)			
DM12_D4_k141_55424	1	dsDNAphage	No	High-quality	100	High-quality draft genome	AAI-based (high-confidence)			
TM05_26wk_k141_187736	0.987	dsDNAphage	No	High-quality	100	High-quality draft genome	AAI-based (high-confidence)		*Firmicutes*	*Tyzzerella nexilis*
TF33_BL_k141_137790	1	dsDNAphage	No	High-quality	90.52	High-quality draft genome	AAI-based (high-confidence)		*Bacteroidetes*	*Bacteroides vulgatus*
TF19_BL_k141_195246	1	dsDNAphage	No	High-quality	100	High-quality draft genome	AAI-based (high-confidence)			
TF14_BL_k141_198259	1	dsDNAphage	No	Complete	100	High-quality draft genome	DTR (high-confidence)			
TM39_12wk_k141_167671	0.987	dsDNAphage	No	Complete	100	High-quality draft genome	DTR (high-confidence)			
TF27_BL_k141_127685	0.993	dsDNAphage	No	High-quality	100	High-quality draft genome	AAI-based (high-confidence)			
TF36_6wk_k141_138313	0.98	dsDNAphage	No	High-quality	100	High-quality draft genome	AAI-based (high-confidence)			
DF12_D5_k141_6854	1	dsDNAphage	No	High-quality	95.91	High-quality draft genome	AAI-based (high-confidence)			
TF47_BL_k141_64390	1	dsDNAphage	No	Complete	100	High-quality draft genome	DTR (high-confidence)	*Crass_like*		
TF01_BL_k141_83586	0.993	dsDNAphage	No	High-quality	91.72	High-quality draft genome	AAI-based (high-confidence)			
DM12_D5_k141_94251	0.88	ssDNA	No	High-quality	100	High-quality draft genome	HMM-based (lower-bound)			
DM07_D6_k141_114616	0.993	dsDNAphage	No	High-quality	100	High-quality draft genome	AAI-based (medium-confidence)			
TM01_6WK_k141_131383	1	dsDNAphage	No	High-quality	100	High-quality draft genome	AAI-based (medium-confidence)			
TF02_6wk_k141_166652	0.993	dsDNAphage	No	High-quality	100	High-quality draft genome	AAI-based (medium-confidence)		*Firmicutes*	*Staphylococcus epidermidis*
TF11_26wk_k141_64422	0.98	dsDNAphage	Yes	High-quality	98.48	High-quality draft genome	AAI-based (high-confidence)		*Firmicutes*	*Dorea longicatena*
TF24_26wk_k141_145003	0.893	dsDNAphage	No	High-quality	100	High-quality draft genome	AAI-based (high-confidence)	*Microviridae*		
DM12_D6_k141_20507	0.987	dsDNAphage	No	High-quality	97.21	High-quality draft genome	AAI-based (high-confidence)			
TF40_BL_k141_53225	0.973	dsDNAphage	No	High-quality	100	High-quality draft genome	AAI-based (high-confidence)			
TF32_26wk_k141_75324	0.987	dsDNAphage	No	High-quality	96.96	High-quality draft genome	AAI-based (high-confidence)			
TM06_6wk_k141_20637	0.987	dsDNAphage	No	High-quality	91.7	High-quality draft genome	AAI-based (high-confidence)		*Firmicutes*	*Blautia obeum*
TF37_6wk_k141_124012	0.993	dsDNAphage	No	High-quality	100	High-quality draft genome	AAI-based (high-confidence)			
TF17_26wk_k141_23920	1	dsDNAphage	No	High-quality	100	High-quality draft genome	AAI-based (high-confidence)			
TF18_BL_k141_96694	0.987	dsDNAphage	No	High-quality	100	High-quality draft genome	AAI-based (high-confidence)			
TF40_6wk_k141_5774	1	dsDNAphage	No	High-quality	94.41	High-quality draft genome	AAI-based (high-confidence)		*Bacteroidetes*	*Bacteroides uniformis*
TF41_26wk_k141_133752	0.993	dsDNAphage	No	Complete	100	High-quality draft genome	DTR (high-confidence)	*Myoviridae*		
TF45_26wk_k141_41927	0.927	ssDNA	No	High-quality	100	High-quality draft genome	HMM-based (lower-bound)			
TF34_12wk_k141_168526	1	dsDNAphage	No	Complete	100	High-quality draft genome	DTR (high-confidence)		*Firmicutes*	*Tyzzerella nexilis*
TF42_BL_k141_109215	0.993	dsDNAphage	No	Complete	100	High-quality draft genome	ITR (high-confidence)			
TM24_26wk_k141_37422	0.993	dsDNAphage	No	Complete	100	High-quality draft genome	DTR (high-confidence)			
TM17_BL_k141_22008	1	dsDNAphage	No	Complete	100	High-quality draft genome	DTR (high-confidence)			
TF35_6wk_k141_37114	1	dsDNAphage	No	High-quality	100	High-quality draft genome	AAI-based (high-confidence)		*Firmicutes*	*Tyzzerella nexilis*
TM09_BL_k141_3239	0.98	dsDNAphage	No	High-quality	93.34	High-quality draft genome	AAI-based (high-confidence)			
TM27_12wk_k141_62643	1	dsDNAphage	No	High-quality	96.87	High-quality draft genome	AAI-based (high-confidence)		*Firmicutes*	*Roseburia intestinalis*
TM35_BL_k141_222994	1	dsDNAphage	No	High-quality	100	High-quality draft genome	AAI-based (high-confidence)			
TF50_26wk_k141_148583	0.993	dsDNAphage	No	Complete	100	High-quality draft genome	DTR (high-confidence)		*Actinobacteria*	*Bifidobacterium adolescentis*
TF22_BL_k141_122122	1	dsDNAphage	No	High-quality	100	High-quality draft genome	AAI-based (high-confidence)	*Myoviridae*		
TF37_12wk_k141_161552	1	dsDNAphage	No	Complete	100	High-quality draft genome	DTR (high-confidence)			
DM03_D3_k141_86702	1	dsDNAphage	Yes	High-quality	100	High-quality draft genome	AAI-based (high-confidence)			
TM36_BL_k141_23034	0.98	dsDNAphage	No	High-quality	98.54	High-quality draft genome	AAI-based (high-confidence)	*Myoviridae*		
TF36_6wk_k141_98280	0.993	dsDNAphage	No	High-quality	100	High-quality draft genome	AAI-based (high-confidence)			
TM01_12wk_k141_21084	0.993	dsDNAphage	No	Complete	100	High-quality draft genome	DTR (high-confidence)		*Firmicutes*	*Megamonas funiformis*
TF41_6wk_k141_208863	1	dsDNAphage	No	Complete	100	High-quality draft genome	DTR (high-confidence)	*Microviridae*		
TM03_26wk_k141_4974	1	dsDNAphage	No	High-quality	98.16	High-quality draft genome	AAI-based (high-confidence)		*Bacteroidetes*	*Prevotella copri*
DF12_D9_k141_64418	1	dsDNAphage	No	High-quality	97.7	High-quality draft genome	AAI-based (medium-confidence)			
TF17_26wk_k141_84228	0.993	dsDNAphage	No	High-quality	100	High-quality draft genome	AAI-based (high-confidence)			
TF36_6wk_k141_189960	0.98	dsDNAphage	Yes	High-quality	97.31	High-quality draft genome	AAI-based (high-confidence)		*Firmicutes*	*Coprococcus comes*
TM27_6wk_k141_19145	0.987	dsDNAphage	No	High-quality	96.41	High-quality draft genome	AAI-based (high-confidence)		*Firmicutes*	*Flavonifractor plautii*
TM05_26wk_k141_74482	1	dsDNAphage	No	High-quality	100	High-quality draft genome	AAI-based (high-confidence)	*Myoviridae*	*Firmicutes*	*Faecalibacterium prausnitzii*
TM30_BL_k141_65637	0.993	dsDNAphage	No	High-quality	94.31	High-quality draft genome	AAI-based (high-confidence)			
TF23_12wk_k141_91970	0.993	dsDNAphage	No	High-quality	92.14	High-quality draft genome	AAI-based (high-confidence)			
TF20_12wk_k141_160505	1	dsDNAphage	Yes	High-quality	99.71	High-quality draft genome	AAI-based (high-confidence)		*Bacteroidetes*	*Prevotella copri*
TM10_12wk_k141_93744	1	dsDNAphage	No	High-quality	100	High-quality draft genome	AAI-based (high-confidence)	*Myoviridae*	*Firmicutes*	*Faecalibacterium prausnitzii*
TM28_26wk_k141_136017	1	dsDNAphage	No	Complete	100	High-quality draft genome	DTR (high-confidence)			
TF35_6wk_k141_185803	0.987	dsDNAphage	No	High-quality	92.6	High-quality draft genome	AAI-based (high-confidence)			
TF06_6WK_k141_83677	1	dsDNAphage	No	Complete	100	High-quality draft genome	DTR (high-confidence)		*Bacteroidetes*	*Bacteroides xylanisolvens*
TF05_12wk_k141_48035	0.987	dsDNAphage	No	High-quality	93.08	High-quality draft genome	AAI-based (high-confidence)		*Firmicutes*	*Blautia obeum*
TF42_12wk_k141_137319	0.987	dsDNAphage	No	High-quality	98.05	High-quality draft genome	AAI-based (high-confidence)	*Myoviridae*	*Firmicutes*	*Faecalibacterium prausnitzii*
TF41_BL_k141_111562	0.993	dsDNAphage	No	Complete	100	High-quality draft genome	DTR (high-confidence)	*Siphoviridae*		
TM15_6wk_k141_63231	0.84	dsDNAphage	No	High-quality	100	High-quality draft genome	AAI-based (high-confidence)			
TF12_6wk_k141_74424	1	dsDNAphage	No	High-quality	100	High-quality draft genome	AAI-based (high-confidence)	*Myoviridae*		
TM33_12wk_k141_64841	0.98	dsDNAphage	No	High-quality	100	High-quality draft genome	AAI-based (medium-confidence)		*Firmicutes*	*Megamonas hypermegale*
TF25_BL_k141_12814	0.993	dsDNAphage	No	Complete	100	High-quality draft genome	DTR (high-confidence)			
TF08_BL_k141_73109	1	dsDNAphage	No	High-quality	93.32	High-quality draft genome	AAI-based (high-confidence)			
DM12_D3_k141_44693	0.987	dsDNAphage	No	High-quality	100	High-quality draft genome	AAI-based (medium-confidence)	*Myoviridae*		
TF20_12wk_k141_144594	0.993	dsDNAphage	No	High-quality	93.8	High-quality draft genome	AAI-based (medium-confidence)			
TM08_26wk_k141_63987	0.54	dsDNAphage	No	High-quality	100	High-quality draft genome	HMM-based (lower-bound)			
TF45_6wk_k141_28625	0.987	dsDNAphage	No	High-quality	100	High-quality draft genome	AAI-based (high-confidence)			
TM35_BL_k141_211301	1	dsDNAphage	No	Complete	100	High-quality draft genome	DTR (high-confidence)			
TF04_BL_k141_169754	1	dsDNAphage	No	Complete	100	High-quality draft genome	DTR (high-confidence)			
TF42_6wk_k141_209285	1	dsDNAphage	No	Complete	100	High-quality draft genome	DTR (high-confidence)		*Firmicutes*	*Blautia obeum*
TF06_BL_k141_51221	1	dsDNAphage	No	High-quality	90.71	High-quality draft genome	AAI-based (high-confidence)			
TM18_12wk_k141_41591	0.987	dsDNAphage	No	High-quality	91.4	High-quality draft genome	AAI-based (high-confidence)			
TF04_BL_k141_39365	0.993	dsDNAphage	No	Complete	100	High-quality draft genome	DTR (high-confidence)			
TF45_12wk_k141_123180	0.953	dsDNAphage	No	High-quality	100	High-quality draft genome	AAI-based (high-confidence)			
TF06_12wk_k141_86417	1	dsDNAphage	No	High-quality	92.67	High-quality draft genome	AAI-based (high-confidence)		*Firmicutes*	*Ruminococcus bromii*
TM16_BL_k141_129376	0.6	dsDNAphage	No	High-quality	100	High-quality draft genome	AAI-based (medium-confidence)			
TF32_26wk_k141_123813	0.987	dsDNAphage	Yes	High-quality	96.95	High-quality draft genome	AAI-based (high-confidence)		*Firmicutes*	*Faecalibacterium prausnitzii*
TM14_BL_k141_50481	0.953	dsDNAphage	No	High-quality	100	High-quality draft genome	AAI-based (medium-confidence)			
TF01_26wk_k141_114883	0.987	dsDNAphage	No	High-quality	98.38	High-quality draft genome	AAI-based (high-confidence)			
TF23_12wk_k141_222111	1	dsDNAphage	Yes	High-quality	94.35	High-quality draft genome	AAI-based (high-confidence)			
TM16_BL_k141_122284	1	dsDNAphage	No	High-quality	100	High-quality draft genome	AAI-based (high-confidence)			
TM14_BL_k141_9153	1	dsDNAphage	No	Complete	100	High-quality draft genome	ITR (high-confidence)	*Podoviridae*		
TF35_12wk_k141_196315	1	dsDNAphage	No	High-quality	100	High-quality draft genome	AAI-based (high-confidence)			
TM16_26wk_k141_142559	0.993	dsDNAphage	No	High-quality	97.91	High-quality draft genome	AAI-based (high-confidence)	*Crass_like*	*Firmicutes*	*Staphylococcus aureus*
TF11_6wk_k141_83592	1	dsDNAphage	No	Complete	100	High-quality draft genome	DTR (high-confidence)		*Actinobacteria*	*Bifidobacterium longum*
TF01_26wk_k141_173443	1	dsDNAphage	No	Complete	100	High-quality draft genome	DTR (high-confidence)	*Microviridae*		
TM32_12wk_k141_4432	0.987	dsDNAphage	No	High-quality	100	High-quality draft genome	AAI-based (high-confidence)		*Firmicutes*	*Blautia obeum*
DM03_D5_k141_82861	1	dsDNAphage	No	High-quality	99.28	High-quality draft genome	AAI-based (high-confidence)	*Siphoviridae*		
TF28_26wk_k141_50239	1	dsDNAphage	No	High-quality	99.23	High-quality draft genome	AAI-based (high-confidence)			
TM21_12wk_k141_10022	1	dsDNAphage	No	High-quality	99.39	High-quality draft genome	AAI-based (high-confidence)		*Firmicutes*	*Anaerobutyricum hallii*
TF34_12wk_k141_125985	0.967	dsDNAphage	No	High-quality	94.7	High-quality draft genome	AAI-based (high-confidence)		*Bacteroidetes*	*Alistipes putredinis*
TF03_BL_k141_13648	1	dsDNAphage	Yes	High-quality	100	High-quality draft genome	AAI-based (high-confidence)			
DF16a_D4_k141_81188	1	ssDNA	No	High-quality	100	High-quality draft genome	HMM-based (lower-bound)			
TM34_26wk_k141_96011	0.987	dsDNAphage	No	High-quality	100	High-quality draft genome	AAI-based (high-confidence)		*Firmicutes*	*Faecalibacterium prausnitzii*
TM09_26wk_k141_82006	0.987	dsDNAphage	No	High-quality	100	High-quality draft genome	AAI-based (high-confidence)	*Myoviridae*	*Firmicutes*	*Faecalibacterium prausnitzii*
TF49_BL_k141_20012	0.993	dsDNAphage	No	High-quality	91.1	High-quality draft genome	AAI-based (high-confidence)			

UViG	VirSorter2 score	Predicted genome type	Provirus	Completeness quality	Completeness (%)	Assembly quality	Completeness approach	Family	Host phylum	Host species
TM04_12wk_k141_18903	1	dsDNAphage	No	Complete	100	High-quality draft genome	DTR (high-confidence)		*Actinobacteria*	*Bifidobacterium pseudocatenulatum*
TF40_6wk_k141_5999	1	dsDNAphage	No	High-quality	100	High-quality draft genome	AAI-based (high-confidence)		*Bacteroidetes*	*Bacteroides xylanisolvens*
TF06_BL_k141_174128	1	dsDNAphage	No	Complete	100	High-quality draft genome	DTR (high-confidence)			
TM21_12wk_k141_129166	0.987	dsDNAphage	No	High-quality	96.62	High-quality draft genome	AAI-based (high-confidence)		*Firmicutes*	*Ruminococcus bromii*
TF12_6wk_k141_64713	0.993	dsDNAphage	No	High-quality	97.39	High-quality draft genome	AAI-based (high-confidence)			
TF02_6wk_k141_164916	1	dsDNAphage	No	High-quality	100	High-quality draft genome	AAI-based (high-confidence)			
TM30_26wk_k141_18709	0.987	dsDNAphage	No	High-quality	100	High-quality draft genome	AAI-based (high-confidence)		*Firmicutes*	*Faecalibacterium prausnitzii*
TF07_26wk_k141_143495	0.98	dsDNAphage	No	High-quality	100	High-quality draft genome	AAI-based (high-confidence)		*Firmicutes*	*Tyzzerella nexilis*
TF39_26wk_k141_42665	0.927	dsDNAphage	No	High-quality	100	High-quality draft genome	AAI-based (high-confidence)		*Firmicutes*	*Ruminococcus bromii*
TM13_6wk_k141_75078	0.993	dsDNAphage	No	Complete	100	High-quality draft genome	DTR (high-confidence)		*Firmicutes*	*Blautia obeum*
TF03_26wk_k141_148633	0.96	dsDNAphage	No	High-quality	92.45	High-quality draft genome	AAI-based (high-confidence)	*Myoviridae*	*Firmicutes*	*Faecalibacterium prausnitzii*
TF03_26wk_k141_266027	1	dsDNAphage	No	High-quality	100	High-quality draft genome	AAI-based (high-confidence)		*Firmicutes*	*Ruminococcus lactaris*
TM18_6wk_k141_114813	0.993	dsDNAphage	No	Complete	100	High-quality draft genome	DTR (high-confidence)	*Microviridae*		
TF30_6wk_k141_202339	1	dsDNAphage	No	High-quality	91.58	High-quality draft genome	AAI-based (high-confidence)		*Bacteroidetes*	*Bacteroides dorei*
TF26_6wk_k141_27552	1	dsDNAphage	No	Complete	100	High-quality draft genome	DTR (high-confidence)		*Actinobacteria*	*Bifidobacterium pseudocatenulatum*
TM07_26wk_k141_147337	0.987	dsDNAphage	No	High-quality	100	High-quality draft genome	AAI-based (high-confidence)			
DF16_D7_k141_140613	0.96	dsDNAphage	No	High-quality	100	High-quality draft genome	AAI-based (medium-confidence)			
TM24_BL_k141_218959	1	dsDNAphage	No	High-quality	92.39	High-quality draft genome	AAI-based (medium-confidence)			
TM09_26wk_k141_37902	1	dsDNAphage	No	High-quality	91	High-quality draft genome	AAI-based (medium-confidence)		*Firmicutes*	*Anaerostipes hadrus*
TF05_6wk_k141_123632	1	dsDNAphage	No	High-quality	98.93	High-quality draft genome	AAI-based (high-confidence)		*Firmicutes*	*Faecalibacterium prausnitzii*
TF12_12wk_k141_152325	1	dsDNAphage	No	Complete	100	High-quality draft genome	DTR (high-confidence)			
TF19_BL_k141_93493	0.52	dsDNAphage	No	High-quality	94.35	High-quality draft genome	AAI-based (high-confidence)			
TM01_BL_k141_70138	0.993	dsDNAphage	No	High-quality	100	High-quality draft genome	AAI-based (high-confidence)			
TF42_12wk_k141_4892	1	dsDNAphage	No	High-quality	100	High-quality draft genome	AAI-based (medium-confidence)			
DF16_D3_k141_247867	0.967	dsDNAphage	No	High-quality	95.08	High-quality draft genome	AAI-based (high-confidence)			
TF01_BL_k141_77709	0.953	dsDNAphage	No	High-quality	91.01	High-quality draft genome	AAI-based (high-confidence)		*Bacteroidetes*	*Bacteroides vulgatus*
TF43_26wk_k141_107901	0.987	dsDNAphage	No	High-quality	100	High-quality draft genome	AAI-based (high-confidence)			
DF17_D3_k141_123490	0.973	ssDNA	No	High-quality	100	High-quality draft genome	HMM-based (lower-bound)			
TF42_6wk_k141_108513	0.987	dsDNAphage	No	High-quality	100	High-quality draft genome	AAI-based (medium-confidence)			
TF04_BL_k141_40649	1	dsDNAphage	No	High-quality	100	High-quality draft genome	AAI-based (high-confidence)	*Myoviridae*		
DM03_D5_k141_17080	0.987	dsDNAphage	Yes	High-quality	100	High-quality draft genome	AAI-based (high-confidence)	*Microviridae*		
TF48_BL_k141_152754	1	dsDNAphage	No	High-quality	100	High-quality draft genome	AAI-based (high-confidence)	*Crass_like*		
TF04_BL_k141_17372	1	dsDNAphage	No	High-quality	90.96	High-quality draft genome	AAI-based (high-confidence)			
TM14_BL_k141_34805	0.993	dsDNAphage	No	Complete	100	High-quality draft genome	DTR (high-confidence)			
DF17b_D2_k141_12489	1	dsDNAphage	No	Complete	100	High-quality draft genome	DTR (high-confidence)			
TF41_BL_k141_54512	0.987	dsDNAphage	No	High-quality	100	High-quality draft genome	AAI-based (high-confidence)			
TF29_BL_k141_29916	1	dsDNAphage	No	High-quality	100	High-quality draft genome	AAI-based (high-confidence)	*Siphoviridae*		
TF22_26wk_k141_44673	0.987	dsDNAphage	No	Complete	100	High-quality draft genome	DTR (high-confidence)			
TF02_6wk_k141_270701	0.987	dsDNAphage	No	Complete	100	High-quality draft genome	DTR (high-confidence)	*Crass_like*	*Firmicutes*	*Staphylococcus epidermidis*
TF45_26wk_k141_69517	0.993	dsDNAphage	No	High-quality	100	High-quality draft genome	AAI-based (medium-confidence)		*Bacteroidetes*	*Bacteroides intestinalis*
TF36_BL_k141_125732	1	dsDNAphage	No	High-quality	97.21	High-quality draft genome	AAI-based (medium-confidence)		*Bacteroidetes*	*Bacteroides fragilis*
TF25_6WK_k141_69492	1	dsDNAphage	No	High-quality	100	High-quality draft genome	AAI-based (high-confidence)	*Myoviridae*	*Proteobacteria*	*Escherichia coli*
TF34_26wk_k141_180812	1	dsDNAphage	No	High-quality	100	High-quality draft genome	AAI-based (high-confidence)			
TM24_BL_k141_59765	1	dsDNAphage	No	High-quality	92.89	High-quality draft genome	AAI-based (high-confidence)			
TF01_BL_k141_45634	0.993	dsDNAphage	No	Complete	100	High-quality draft genome	DTR (high-confidence)			
DF16_D6_k141_260134	1	dsDNAphage	No	High-quality	95.18	High-quality draft genome	AAI-based (high-confidence)			
DF12_D2_k141_8632	1	dsDNAphage	Yes	High-quality	100	High-quality draft genome	AAI-based (high-confidence)			
TF19_26wk_k141_164496	0.993	dsDNAphage	Yes	High-quality	91.12	High-quality draft genome	AAI-based (medium-confidence)		*Firmicutes*	*Roseburia inulinivorans*
TM30_26wk_k141_10145	0.993	dsDNAphage	No	Complete	100	High-quality draft genome	DTR (high-confidence)		*Firmicutes*	*Faecalibacterium prausnitzii*
TM13_12wk_k141_61983	1	dsDNAphage	No	High-quality	96.47	High-quality draft genome	AAI-based (high-confidence)		*Bacteroidetes*	*Prevotella copri*
TF40_12wk_k141_7234	1	dsDNAphage	No	High-quality	100	High-quality draft genome	AAI-based (high-confidence)			
TM32_BL_k141_12693	1	dsDNAphage	No	High-quality	100	High-quality draft genome	AAI-based (high-confidence)			
TF12_6wk_k141_140624	0.973	dsDNAphage	No	High-quality	100	High-quality draft genome	AAI-based (medium-confidence)		*Firmicutes*	*Roseburia intestinalis*
TF38_6wk_k141_7534	0.993	dsDNAphage	No	High-quality	100	High-quality draft genome	AAI-based (high-confidence)			
TF16_BL_k141_82309	0.98	dsDNAphage	Yes	High-quality	97.3	High-quality draft genome	AAI-based (high-confidence)			
TF40_BL_k141_90947	0.98	dsDNAphage	No	High-quality	100	High-quality draft genome	AAI-based (high-confidence)			
TF25_26wk_k141_72520	1	dsDNAphage	No	High-quality	100	High-quality draft genome	AAI-based (high-confidence)		*Actinobacteria*	*Bifidobacterium pseudocatenulatum*
TF41_26wk_k141_21634	0.993	dsDNAphage	No	Complete	100	High-quality draft genome	DTR (high-confidence)	*Crass_like*		
TM18_BL_k141_69432	1	dsDNAphage	No	High-quality	98.7	High-quality draft genome	AAI-based (high-confidence)			
TF28_BL_k141_5465	1	dsDNAphage	No	High-quality	100	High-quality draft genome	AAI-based (high-confidence)			
TM18_26wk_k141_36774	1	dsDNAphage	No	High-quality	100	High-quality draft genome	AAI-based (high-confidence)			
DM08_D2_k141_117788	0.993	dsDNAphage	No	High-quality	100	High-quality draft genome	AAI-based (high-confidence)			
TM05_26wk_k141_115102	1	dsDNAphage	No	High-quality	100	High-quality draft genome	AAI-based (high-confidence)			
DM08_D4_k141_132874	1	dsDNAphage	No	Complete	100	High-quality draft genome	DTR (high-confidence)			
TF26_BL_k141_43273	0.74	dsDNAphage	No	High-quality	100	High-quality draft genome	HMM-based (lower-bound)			
DM12_D3_k141_18221	0.993	dsDNAphage	No	High-quality	100	High-quality draft genome	AAI-based (high-confidence)			
TM16_26wk_k141_18044	1	dsDNAphage	No	High-quality	100	High-quality draft genome	AAI-based (high-confidence)		*Firmicutes*	*Ruminococcus bromii*
TM34_6wk_k141_115380	1	dsDNAphage	No	High-quality	100	High-quality draft genome	AAI-based (medium-confidence)			
TM27_6wk_k141_177973	1	dsDNAphage	No	High-quality	100	High-quality draft genome	AAI-based (high-confidence)			
TF20_BL_k141_193097	0.993	dsDNAphage	No	Complete	100	High-quality draft genome	DTR (high-confidence)			
TF15_12wk_k141_95029	0.98	dsDNAphage	No	High-quality	98.47	High-quality draft genome	AAI-based (high-confidence)	*Siphoviridae*	*Firmicutes*	*Ruminococcus bromii*
TF15_26wk_k141_73975	0.987	dsDNAphage	No	High-quality	97.39	High-quality draft genome	AAI-based (high-confidence)			
TF34_BL_k141_20299	0.993	dsDNAphage	No	High-quality	98.84	High-quality draft genome	AAI-based (high-confidence)	*Siphoviridae*		
TM20_26wk_k141_19622	0.987	dsDNAphage	No	Complete	100	High-quality draft genome	DTR (high-confidence)			
TM24_6wk_k141_101680	0.993	dsDNAphage	No	Complete	100	High-quality draft genome	DTR (high-confidence)	*Crass_like*	*Bacteroidetes*	*Parabacteroides distasonis*
DM12_D4_k141_44761	1	dsDNAphage	No	Complete	100	High-quality draft genome	DTR (high-confidence)			
DM07_D6_k141_69013	0.993	dsDNAphage	No	High-quality	97.55	High-quality draft genome	AAI-based (high-confidence)	*Crass_like*		
TF18_26wk_k141_4359	0.993	dsDNAphage	Yes	High-quality	100	High-quality draft genome	AAI-based (high-confidence)		*Firmicutes*	*Faecalibacterium prausnitzii*
TM22_BL_k141_99182	0.993	dsDNAphage	No	Complete	100	High-quality draft genome	DTR (medium-confidence)			
TM16_26wk_k141_34299	1	dsDNAphage	No	Complete	100	High-quality draft genome	DTR (high-confidence)	*Siphoviridae*	*Bacteroidetes*	*Bacteroides clarus*
TF01_BL_k141_27375	0.987	dsDNAphage	No	High-quality	100	High-quality draft genome	AAI-based (medium-confidence)			
TF30_26wk_k141_119382	0.987	dsDNAphage	No	High-quality	100	High-quality draft genome	AAI-based (high-confidence)			
TM33_6wk_k141_33998	0.993	dsDNAphage	No	High-quality	94.83	High-quality draft genome	AAI-based (high-confidence)		*Actinobacteria*	*Bifidobacterium adolescentis*
TF16_26wk_k141_7919	1	dsDNAphage	No	High-quality	96.27	High-quality draft genome	AAI-based (high-confidence)	*Siphoviridae*	*Firmicutes*	*Streptococcus anginosus*
TM16_26wk_k141_231153	1	dsDNAphage	No	Complete	100	High-quality draft genome	DTR (high-confidence)	*Crass_like*	*Firmicutes*	*Staphylococcus aureus*
TF05_BL_k141_94355	0.993	dsDNAphage	No	High-quality	97.42	High-quality draft genome	AAI-based (high-confidence)	*Siphoviridae*		
TF37_12wk_k141_62943	0.98	dsDNAphage	No	High-quality	100	High-quality draft genome	AAI-based (high-confidence)			
TF39_26wk_k141_133657	0.92	dsDNAphage	No	High-quality	100	High-quality draft genome	AAI-based (high-confidence)		*Firmicutes*	*Staphylococcus aureus*
TF07_6wk_k141_132234	0.94	ssDNA	No	High-quality	100	High-quality draft genome	HMM-based (lower-bound)			
TF06_26wk_k141_164813	0.993	dsDNAphage	No	High-quality	100	High-quality draft genome	AAI-based (medium-confidence)	*Podoviridae*		
TM24_26wk_k141_15180	0.993	dsDNAphage	No	High-quality	100	High-quality draft genome	AAI-based (high-confidence)			
TM30_6wk_k141_27613	1	dsDNAphage	No	High-quality	98.23	High-quality draft genome	AAI-based (high-confidence)	*Siphoviridae*	*Bacteroidetes*	*Bacteroides massiliensis*
DF14_D5_k141_126398	1	dsDNAphage	No	Complete	100	High-quality draft genome	DTR (high-confidence)			
TM13_6wk_k141_49548	0.847	dsDNAphage	No	High-quality	100	High-quality draft genome	AAI-based (high-confidence)			
TF29_6WK_k141_113157	0.98	dsDNAphage	No	High-quality	100	High-quality draft genome	AAI-based (high-confidence)		*Firmicutes*	*Butyrivibrio crossotus*
DM03_D4_k141_30619	0.98	dsDNAphage	No	High-quality	100	High-quality draft genome	AAI-based (high-confidence)			
DM03_D1_k141_93346	0.94	dsDNAphage	No	Complete	100	High-quality draft genome	DTR (high-confidence)			
TM21_6wk_k141_88983	0.993	dsDNAphage	No	High-quality	94.55	High-quality draft genome	AAI-based (high-confidence)			
DM07_D2_k141_98811	1	dsDNAphage	No	High-quality	100	High-quality draft genome	AAI-based (high-confidence)			
TM05_6wk_k141_6893	0.993	dsDNAphage	No	High-quality	99.54	High-quality draft genome	AAI-based (high-confidence)			
DF16_D9_k141_99627	1	dsDNAphage	No	Complete	100	High-quality draft genome	DTR (high-confidence)			
TM16_12wk_k141_110359	1	dsDNAphage	No	High-quality	100	High-quality draft genome	AAI-based (high-confidence)	*Myoviridae*		
DF16_D6_k141_138752	0.993	dsDNAphage	Yes	Complete	100	High-quality draft genome	Provirus (high-confidence)			
TM13_BL_k141_26623	0.987	dsDNAphage	No	High-quality	90.51	High-quality draft genome	AAI-based (high-confidence)			
TF49_BL_k141_188979	0.993	dsDNAphage	No	High-quality	100	High-quality draft genome	AAI-based (medium-confidence)	*Myoviridae*		

UViG	VirSorter2 score	Predicted genome type	Provirus	Completeness quality	Completeness (%)	Assembly quality	Completeness approach	Family	Host phylum	Host species
TF36_BL_k141_26183	0.98	dsDNAphage	Yes	High-quality	100	High-quality draft genome	AAI-based (high-confidence)			
TF06_12wk_k141_137063	1	dsDNAphage	No	High-quality	96.91	High-quality draft genome	AAI-based (high-confidence)		*Bacteroidetes*	*Bacteroides xylanisolvens*
DM03_D5_k141_34702	0.993	dsDNAphage	Yes	High-quality	100	High-quality draft genome	AAI-based (high-confidence)			
TF06_26wk_k141_58487	0.993	dsDNAphage	No	High-quality	97.76	High-quality draft genome	AAI-based (high-confidence)			
TF41_BL_k141_1239	0.98	dsDNAphage	No	High-quality	95.11	High-quality draft genome	AAI-based (high-confidence)			
TF28_BL_k141_54429	1	dsDNAphage	No	High-quality	100	High-quality draft genome	AAI-based (high-confidence)	*Myoviridae*		
TM23_12wk_k141_61369	0.96	ssDNA	No	High-quality	100	High-quality draft genome	HMM-based (lower-bound)			
TF17_26wk_k141_27412	0.98	dsDNAphage	Yes	High-quality	99.86	High-quality draft genome	AAI-based (high-confidence)	*Myoviridae*	*Firmicutes*	*Phascolarctobacterium succinatutens*
TM06_12wk_k141_147377	1	dsDNAphage	No	High-quality	94.19	High-quality draft genome	AAI-based (high-confidence)		*Firmicutes*	*Phascolarctobacterium succinatutens*
TF14_6wk_k141_212491	1	dsDNAphage	No	Complete	100	High-quality draft genome	DTR (high-confidence)			
TM03_BL_k141_76794	1	dsDNAphage	No	High-quality	100	High-quality draft genome	AAI-based (high-confidence)			
TF35_6wk_k141_20828	1	dsDNAphage	No	Complete	100	High-quality draft genome	DTR (high-confidence)			
TF19_12wk_k141_45850	0.96	dsDNAphage	No	Complete	100	High-quality draft genome	DTR (high-confidence)			
TF49_6wk_k141_156915	1	dsDNAphage	No	High-quality	95.16	High-quality draft genome	AAI-based (medium-confidence)		*Firmicutes*	*Staphylococcus aureus*
DM07_D1_k141_809	0.98	dsDNAphage	No	High-quality	100	High-quality draft genome	AAI-based (high-confidence)			
TF50_6wk_k141_220701	1	dsDNAphage	No	Complete	100	High-quality draft genome	DTR (high-confidence)	*Siphoviridae*		
TF50_6wk_k141_72099	1	dsDNAphage	No	High-quality	92.85	High-quality draft genome	AAI-based (high-confidence)		*Firmicutes*	*Coprococcus comes*
TM15_6wk_k141_93895	0.987	dsDNAphage	No	High-quality	93.56	High-quality draft genome	AAI-based (high-confidence)	*Siphoviridae*	*Firmicutes*	*Faecalibacterium prausnitzii*
TF35_BL_k141_86399	0.993	dsDNAphage	No	High-quality	100	High-quality draft genome	AAI-based (high-confidence)			
TF45_6wk_k141_25007	0.973	dsDNAphage	No	High-quality	100	High-quality draft genome	AAI-based (high-confidence)		*Bacteroidetes*	*Bacteroides xylanisolvens*
TF34_6wk_k141_60407	0.993	dsDNAphage	No	High-quality	100	High-quality draft genome	AAI-based (medium-confidence)			
TM24_26wk_k141_18632	1	dsDNAphage	No	High-quality	99.31	High-quality draft genome	AAI-based (high-confidence)			
TF24_12wk_k141_24571	0.987	dsDNAphage	Yes	High-quality	100	High-quality draft genome	AAI-based (high-confidence)		*Firmicutes*	*Ruminococcus bromii*
TF04_BL_k141_152263	0.993	dsDNAphage	No	High-quality	100	High-quality draft genome	AAI-based (high-confidence)			
TM01_26wk_k141_116885	1	dsDNAphage	No	High-quality	99.63	High-quality draft genome	AAI-based (high-confidence)		*Firmicutes*	*Ruminococcus bromii*
TF12_6wk_k141_156256	0.987	dsDNAphage	No	Complete	100	High-quality draft genome	DTR (high-confidence)	*Microviridae*		
TM05_BL_k141_137060	1	dsDNAphage	No	Complete	100	High-quality draft genome	DTR (high-confidence)	*Myoviridae*		
TM10_BL_k141_2577	1	dsDNAphage	No	High-quality	100	High-quality draft genome	AAI-based (high-confidence)	*Myoviridae*		
TM08_BL_k141_90468	0.993	dsDNAphage	No	High-quality	95.01	High-quality draft genome	AAI-based (medium-confidence)			
TM36_12wk_k141_48778	0.993	dsDNAphage	No	High-quality	92.23	High-quality draft genome	AAI-based (high-confidence)		*Firmicutes*	*Butyrivibrio crossotus*
TM08_12wk_k141_163892	1	dsDNAphage	No	High-quality	100	High-quality draft genome	AAI-based (high-confidence)			
TF18_12wk_k141_156214	1	dsDNAphage	Yes	High-quality	99.21	High-quality draft genome	AAI-based (high-confidence)			
TF02_BL_k141_249967	1	dsDNAphage	Yes	Complete	100	High-quality draft genome	DTR (medium-confidence)			
TM03_12wk_k141_52605	1	dsDNAphage	No	High-quality	100	High-quality draft genome	AAI-based (high-confidence)		*Firmicutes*	*Tyzzerella nexilis*
TF07_6wk_k141_155442	1	dsDNAphage	No	High-quality	92.42	High-quality draft genome	AAI-based (high-confidence)	*Siphoviridae*	*Firmicutes*	*Faecalibacterium prausnitzii*
DF12_D3_k141_14657	1	dsDNAphage	No	High-quality	100	High-quality draft genome	AAI-based (high-confidence)			
TF09_6WK_k141_59142	1	dsDNAphage	No	High-quality	100	High-quality draft genome	AAI-based (high-confidence)		*Bacteroidetes*	*Prevotella multiformis*
TM39_26wk_k141_97164	1	dsDNAphage	No	High-quality	99.22	High-quality draft genome	AAI-based (high-confidence)		*Firmicutes*	*Ruminococcus bromii*
TF29_26wk_k141_58048	1	dsDNAphage	No	High-quality	100	High-quality draft genome	AAI-based (high-confidence)		*Actinobacteria*	*Bifidobacterium longum*
TF27_6wk_k141_222548	1	dsDNAphage	No	Complete	100	High-quality draft genome	DTR (medium-confidence)	*Podoviridae*	*Firmicutes*	*Clostridium sporogenes*
TF22_BL_k141_22678	0.993	dsDNAphage	No	High-quality	95.69	High-quality draft genome	AAI-based (high-confidence)		*Firmicutes*	*Coprococcus comes*
DF16_D2_k141_127400	0.98	dsDNAphage	No	High-quality	100	High-quality draft genome	AAI-based (high-confidence)			
DF17b_D2_k141_18619	1	dsDNAphage	Yes	Complete	100	High-quality draft genome	Provirus (medium-confidence)	*Myoviridae*		
TF41_BL_k141_50668	1	dsDNAphage	No	High-quality	95.41	High-quality draft genome	AAI-based (high-confidence)			
TM34_6wk_k141_26193	0.993	dsDNAphage	No	Complete	100	High-quality draft genome	DTR (high-confidence)		*Firmicutes*	*Blautia obeum*
TF44_6wk_k141_7681	1	dsDNAphage	No	Complete	100	High-quality draft genome	DTR (high-confidence)		*Bacteroidetes*	*Prevotella copri*
DM07_D4_k141_185516	0.993	dsDNAphage	Yes	High-quality	100	High-quality draft genome	AAI-based (high-confidence)			
TF25_12wk_k141_102159	0.987	dsDNAphage	No	High-quality	100	High-quality draft genome	AAI-based (high-confidence)			
TM14_12wk_k141_42089	1	dsDNAphage	No	High-quality	100	High-quality draft genome	AAI-based (medium-confidence)			
TM20_12wk_k141_18780	1	dsDNAphage	No	Complete	100	High-quality draft genome	DTR (high-confidence)		*Firmicutes*	*Blautia obeum*
TF18_26wk_k141_72469	1	dsDNAphage	No	High-quality	100	High-quality draft genome	AAI-based (high-confidence)		*Firmicutes*	*Roseburia intestinalis*
TM30_12wk_k141_61193	1	dsDNAphage	No	High-quality	100	High-quality draft genome	AAI-based (high-confidence)		*Firmicutes*	*Blautia obeum*
TF09_6WK_k141_197715	1	dsDNAphage	No	Complete	100	High-quality draft genome	DTR (high-confidence)		*Firmicutes*	*Roseburia intestinalis*
TF44_6wk_k141_74504	0.867	dsDNAphage	No	High-quality	95.5	High-quality draft genome	AAI-based (high-confidence)		*Firmicutes*	*Anaerobutyricum hallii*
TF48_12wk_k141_41655	1	dsDNAphage	No	High-quality	95.45	High-quality draft genome	AAI-based (high-confidence)			
TF21_BL_k141_91460	1	dsDNAphage	Yes	High-quality	100	High-quality draft genome	AAI-based (high-confidence)			
TM25_26wk_k141_73028	0.9	dsDNAphage	No	High-quality	100	High-quality draft genome	AAI-based (medium-confidence)			
TF37_26wk_k141_78985	1	dsDNAphage	Yes	High-quality	91.99	High-quality draft genome	AAI-based (high-confidence)		*Firmicutes*	*Dorea longicatena*
TF37_BL_k141_107475	1	dsDNAphage	Yes	High-quality	98.49	High-quality draft genome	AAI-based (high-confidence)			
TF37_BL_k141_134872	1	dsDNAphage	Yes	High-quality	97.74	High-quality draft genome	AAI-based (high-confidence)		*Firmicutes*	*Anaerostipes hadrus*
TF37_BL_k141_70116	0.987	dsDNAphage	Yes	High-quality	99.94	High-quality draft genome	AAI-based (high-confidence)			
TF37_BL_k141_76854	0.993	dsDNAphage	Yes	High-quality	98.98	High-quality draft genome	AAI-based (high-confidence)			
TF38_6wk_k141_146440	1	dsDNAphage	Yes	High-quality	90.63	High-quality draft genome	AAI-based (high-confidence)			
TF40_12wk_k141_106649	0.993	dsDNAphage	Yes	High-quality	100	High-quality draft genome	AAI-based (high-confidence)			
TF40_6wk_k141_91768	0.987	dsDNAphage	Yes	High-quality	95.99	High-quality draft genome	AAI-based (high-confidence)		*Firmicutes*	*Faecalibacterium prausnitzii*
TF41_12wk_k141_144134	0.987	dsDNAphage	Yes	High-quality	100	High-quality draft genome	AAI-based (high-confidence)			
TF41_26wk_k141_12202	0.993	dsDNAphage	Yes	High-quality	100	High-quality draft genome	AAI-based (high-confidence)			
TF42_12wk_k141_71595	0.993	dsDNAphage	Yes	High-quality	96.88	High-quality draft genome	AAI-based (high-confidence)			
TF42_26wk_k141_125867	1	dsDNAphage	Yes	High-quality	100	High-quality draft genome	AAI-based (high-confidence)			
TF42_26wk_k141_166229	1	dsDNAphage	Yes	High-quality	98.56	High-quality draft genome	AAI-based (high-confidence)		*Firmicutes*	*Eubacterium ventriosum*
TF42_6wk_k141_127766	0.993	dsDNAphage	Yes	High-quality	100	High-quality draft genome	AAI-based (high-confidence)		*Firmicutes*	*Staphylococcus aureus*
TF42_BL_k141_19766	1	ssDNA	Yes	High-quality	100	High-quality draft genome	AAI-based (high-confidence)	*Circoviridae*		
TF43_12wk_k141_95207	0.993	dsDNAphage	Yes	Complete	100	High-quality draft genome	DTR (medium-confidence)		*Firmicutes*	*Ruminococcus bromii*
TF43_26wk_k141_95639	0.973	dsDNAphage	Yes	High-quality	100	High-quality draft genome	AAI-based (high-confidence)			
TF43_6wk_k141_148312	1	dsDNAphage	Yes	High-quality	100	High-quality draft genome	AAI-based (high-confidence)			
TF43_BL_k141_35049	0.987	dsDNAphage	Yes	High-quality	100	High-quality draft genome	AAI-based (high-confidence)	*Siphoviridae*		
TF44_12wk_k141_28497	0.993	dsDNAphage	Yes	High-quality	93.94	High-quality draft genome	AAI-based (medium-confidence)		*Firmicutes*	*Roseburia intestinalis*
TF44_12wk_k141_43901	0.993	dsDNAphage	Yes	High-quality	100	High-quality draft genome	AAI-based (high-confidence)		*Actinobacteria*	*Bifidobacterium adolescentis*
TF46_12wk_k141_89080	0.973	dsDNAphage	Yes	High-quality	92.14	High-quality draft genome	AAI-based (high-confidence)		*Firmicutes*	*Dorea longicatena*
TF46_6wk_k141_102721	0.993	dsDNAphage	Yes	High-quality	100	High-quality draft genome	AAI-based (high-confidence)		*Actinobacteria*	*Bifidobacterium longum*
TF46_BL_k141_110370	0.993	dsDNAphage	Yes	High-quality	100	High-quality draft genome	AAI-based (high-confidence)	*Myoviridae*	*Firmicutes*	*Faecalibacterium prausnitzii*
TF46_BL_k141_118276	0.993	dsDNAphage	Yes	High-quality	100	High-quality draft genome	AAI-based (high-confidence)			
TF46_BL_k141_38989	0.993	dsDNAphage	Yes	Complete	100	High-quality draft genome	Provirus (medium-confidence)			
TF46_BL_k141_4714	1	dsDNAphage	Yes	High-quality	100	High-quality draft genome	AAI-based (high-confidence)			
TF47_12wk_k141_47082	1	dsDNAphage	Yes	High-quality	91.74	High-quality draft genome	AAI-based (high-confidence)		*Actinobacteria*	*Bifidobacterium bifidum*
TF47_6wk_k141_133679	0.98	dsDNAphage	Yes	High-quality	97.37	High-quality draft genome	AAI-based (high-confidence)		*Firmicutes*	*Dorea formicigenerans*
TF47_BL_k141_41904	0.98	dsDNAphage	Yes	Complete	100	High-quality draft genome	Provirus (high-confidence)	*Myoviridae*		
TF48_26wk_k141_71318	0.993	dsDNAphage	Yes	High-quality	100	High-quality draft genome	AAI-based (high-confidence)			
TF48_6wk_k141_3254	0.96	dsDNAphage	Yes	High-quality	92.33	High-quality draft genome	AAI-based (high-confidence)			
TF48_BL_k141_133798	0.987	dsDNAphage	Yes	High-quality	98.33	High-quality draft genome	AAI-based (high-confidence)			
TF49_6wk_k141_30886	0.993	dsDNAphage	Yes	High-quality	100	High-quality draft genome	AAI-based (high-confidence)	*Myoviridae*	*Firmicutes*	*Ruminococcus bromii*
TF49_6wk_k141_7353	0.987	dsDNAphage	Yes	High-quality	90.45	High-quality draft genome	AAI-based (high-confidence)		*Firmicutes*	*Faecalibacterium prausnitzii*
TF49_6wk_k141_93399	0.993	dsDNAphage	Yes	High-quality	100	High-quality draft genome	AAI-based (high-confidence)			
TF49_BL_k141_80100	0.973	dsDNAphage	Yes	High-quality	100	High-quality draft genome	AAI-based (high-confidence)			
TF50_26wk_k141_164910	0.987	dsDNAphage	Yes	High-quality	95.98	High-quality draft genome	AAI-based (high-confidence)		*Firmicutes*	*Phascolarctobacterium succinatutens*
TF51_6wk_k141_120240	0.993	dsDNAphage	Yes	High-quality	95.67	High-quality draft genome	AAI-based (high-confidence)		*Firmicutes*	*Streptococcus anginosus*
TF51_6wk_k141_27821	0.987	dsDNAphage	Yes	High-quality	92.38	High-quality draft genome	AAI-based (high-confidence)			
TF51_BL_k141_4535	0.993	dsDNAphage	Yes	High-quality	100	High-quality draft genome	AAI-based (high-confidence)			
TF51_BL_k141_83967	0.987	dsDNAphage	Yes	High-quality	100	High-quality draft genome	AAI-based (high-confidence)			
TM03_BL_k141_187643	0.993	dsDNAphage	Yes	High-quality	90.11	High-quality draft genome	AAI-based (high-confidence)			
TM04_12wk_k141_112676	0.993	dsDNAphage	Yes	High-quality	100	High-quality draft genome	AAI-based (high-confidence)	*Myoviridae*		
TM04_12wk_k141_121875	0.98	dsDNAphage	Yes	High-quality	100	High-quality draft genome	AAI-based (high-confidence)		*Firmicutes*	*Streptococcus anginosus*
TM04_12wk_k141_1739	0.987	dsDNAphage	Yes	High-quality	100	High-quality draft genome	AAI-based (high-confidence)		*Bacteroidetes*	*Bacteroides caccae*
TM04_26wk_k141_153365	0.973	dsDNAphage	Yes	High-quality	100	High-quality draft genome	AAI-based (high-confidence)		*Firmicutes*	*Faecalibacterium prausnitzii*
TM04_6wk_k141_93255	0.987	dsDNAphage	Yes	High-quality	90.37	High-quality draft genome	AAI-based (high-confidence)		*Firmicutes*	*Ruminococcus bromii*

UViG	VirSorter2 score	Predicted genome type	Provirus	Completeness quality	Completeness (%)	Assembly quality	Completeness approach	Family	Host phylum	Host species
TM04_BL_k141_51317	0.987	dsDNAphage	Yes	High-quality	100	High-quality draft genome	AAI-based (high-confidence)			
TM05_6wk_k141_170751	1	dsDNAphage	Yes	High-quality	100	High-quality draft genome	AAI-based (high-confidence)		*Firmicutes*	*Lactobacillus ruminis*
TM06_12wk_k141_56887	0.98	dsDNAphage	Yes	High-quality	100	High-quality draft genome	AAI-based (high-confidence)		*Firmicutes*	*Faecalibacterium prausnitzii*
TM06_26wk_k141_40999	0.993	dsDNAphage	Yes	High-quality	100	High-quality draft genome	AAI-based (high-confidence)			
TM06_26wk_k141_54758	0.993	dsDNAphage	Yes	High-quality	96.71	High-quality draft genome	AAI-based (high-confidence)		*Firmicutes*	*Staphylococcus epidermidis*
TM06_26wk_k141_80864	0.993	dsDNAphage	Yes	High-quality	100	High-quality draft genome	AAI-based (high-confidence)			
TM06_6wk_k141_23281	1	dsDNAphage	Yes	High-quality	100	High-quality draft genome	AAI-based (medium-confidence)	*Siphoviridae*	*Firmicutes*	*Staphylococcus aureus*
TM06_6wk_k141_81743	0.927	dsDNAphage	Yes	High-quality	100	High-quality draft genome	AAI-based (medium-confidence)			
TM07_12wk_k141_52208	0.987	dsDNAphage	Yes	Complete	100	High-quality draft genome	Provirus (high-confidence)	*Siphoviridae*	*Firmicutes*	*Faecalibacterium prausnitzii*
TM07_6wk_k141_201457	0.987	dsDNAphage	Yes	High-quality	97.67	High-quality draft genome	AAI-based (high-confidence)	*Myoviridae*		
TM08_26wk_k141_11949	0.993	dsDNAphage	Yes	High-quality	91.5	High-quality draft genome	AAI-based (high-confidence)	*Microviridae*		
TM08_6wk_k141_75625	1	dsDNAphage	Yes	High-quality	100	High-quality draft genome	AAI-based (high-confidence)	*Myoviridae*		
TM08_BL_k141_118205	0.98	dsDNAphage	Yes	High-quality	96.39	High-quality draft genome	AAI-based (high-confidence)			
TM09_6wk_k141_88720	0.973	ssDNA	Yes	High-quality	100	High-quality draft genome	HMM-based (lower-bound)			
TM09_BL_k141_80919	0.993	dsDNAphage	Yes	High-quality	98.51	High-quality draft genome	AAI-based (high-confidence)			
TM10_BL_k141_2265	0.987	dsDNAphage	Yes	High-quality	100	High-quality draft genome	AAI-based (medium-confidence)		*Bacteroidetes*	*Bacteroides xylanisolvens*
TM12_12wk_k141_97202	0.98	dsDNAphage	Yes	High-quality	94.33	High-quality draft genome	AAI-based (high-confidence)		*Firmicutes*	*Faecalibacterium prausnitzii*
TM12_BL_k141_14288	0.98	dsDNAphage	Yes	High-quality	92.25	High-quality draft genome	AAI-based (high-confidence)			
TM12_BL_k141_60911	1	dsDNAphage	Yes	High-quality	94.51	High-quality draft genome	AAI-based (high-confidence)		*Firmicutes*	*Dorea formicigenerans*
TM13_26wk_k141_61821	0.867	dsDNAphage	Yes	High-quality	100	High-quality draft genome	AAI-based (high-confidence)	*Microviridae*		
TM13_BL_k141_123531	0.9	dsDNAphage	Yes	High-quality	100	High-quality draft genome	AAI-based (high-confidence)	*Microviridae*		
TM13_BL_k141_15337	0.987	dsDNAphage	Yes	High-quality	100	High-quality draft genome	AAI-based (high-confidence)		*Firmicutes*	*Faecalibacterium prausnitzii*
TM13_BL_k141_81986	1	dsDNAphage	Yes	High-quality	100	High-quality draft genome	AAI-based (high-confidence)		*Firmicutes*	*Faecalibacterium prausnitzii*
TM14_12wk_k141_53427	1	dsDNAphage	Yes	High-quality	98.66	High-quality draft genome	AAI-based (high-confidence)	*Siphoviridae*	*Firmicutes*	*Faecalibacterium prausnitzii*
TM14_26wk_k141_10916	0.987	dsDNAphage	Yes	Complete	100	High-quality draft genome	Provirus (high-confidence)	*Siphoviridae*		
TM14_6wk_k141_135968	0.993	dsDNAphage	Yes	High-quality	100	High-quality draft genome	AAI-based (high-confidence)		*Firmicutes*	*Blautia obeum*
TM16_6WK_k141_39087	1	dsDNAphage	Yes	High-quality	100	High-quality draft genome	AAI-based (high-confidence)	*Myoviridae*		
TM18_12wk_k141_28700	0.987	dsDNAphage	Yes	High-quality	99.74	High-quality draft genome	AAI-based (high-confidence)	*Podoviridae*		
TM18_26wk_k141_69502	0.993	dsDNAphage	Yes	Complete	100	High-quality draft genome	DTR (high-confidence)		*Firmicutes*	*Roseburia inulinivorans*
TM18_BL_k141_68032	0.993	dsDNAphage	Yes	High-quality	98.88	High-quality draft genome	AAI-based (high-confidence)			
TM18_BL_k141_71605	0.993	dsDNAphage	Yes	High-quality	98	High-quality draft genome	AAI-based (high-confidence)			
TM19_BL_k141_104926	1	dsDNAphage	Yes	High-quality	100	High-quality draft genome	AAI-based (high-confidence)	*Myoviridae*		
TM20_12wk_k141_92644	0.94	dsDNAphage	Yes	High-quality	100	High-quality draft genome	AAI-based (high-confidence)	*Microviridae*		
TM20_26wk_k141_10025	1	dsDNAphage	Yes	High-quality	100	High-quality draft genome	AAI-based (high-confidence)		*Firmicutes*	*Coprococcus comes*
TM20_26wk_k141_16787	1	dsDNAphage	Yes	High-quality	90.92	High-quality draft genome	AAI-based (high-confidence)		*Bacteroidetes*	*Prevotella disiens*
TM20_26wk_k141_59709	0.993	dsDNAphage	Yes	High-quality	95.4	High-quality draft genome	AAI-based (high-confidence)			
TM20_6wk_k141_80689	1	dsDNAphage	Yes	High-quality	100	High-quality draft genome	AAI-based (high-confidence)	*Myoviridae*	*Firmicutes*	*Roseburia inulinivorans*
TM20_BL_k141_177337	1	dsDNAphage	Yes	High-quality	100	High-quality draft genome	AAI-based (high-confidence)	*Siphoviridae*	*Firmicutes*	*Streptococcus pyogenes*
TM22_6wk_k141_125897	0.993	dsDNAphage	Yes	High-quality	94.41	High-quality draft genome	AAI-based (high-confidence)		*Bacteroidetes*	*Prevotella disiens*
TM23_12wk_k141_9965	0.98	dsDNAphage	Yes	High-quality	100	High-quality draft genome	AAI-based (high-confidence)		*Actinobacteria*	*Bifidobacterium longum*
TM23_26wk_k141_15002	0.993	dsDNAphage	Yes	High-quality	100	High-quality draft genome	AAI-based (medium-confidence)			
TM23_26wk_k141_67534	1	dsDNAphage	Yes	High-quality	100	High-quality draft genome	AAI-based (high-confidence)		*Bacteroidetes*	*Bacteroides vulgatus*
TM24_12wk_k141_15888	1	dsDNAphage	Yes	High-quality	99.21	High-quality draft genome	AAI-based (high-confidence)	*Siphoviridae*	*Firmicutes*	*Staphylococcus aureus*
TM24_12wk_k141_16378	1	dsDNAphage	Yes	Complete	100	High-quality draft genome	DTR (high-confidence)	*Siphoviridae*	*Firmicutes*	*Streptococcus pyogenes*
TM24_12wk_k141_52913	0.993	dsDNAphage	Yes	High-quality	94.09	High-quality draft genome	AAI-based (medium-confidence)		*Firmicutes*	*Ruminococcus bromii*
TM24_12wk_k141_53955	0.987	dsDNAphage	Yes	High-quality	100	High-quality draft genome	AAI-based (high-confidence)		*Firmicutes*	*Ruminococcus lactaris*
TM25_12wk_k141_14296	0.987	dsDNAphage	Yes	High-quality	100	High-quality draft genome	AAI-based (high-confidence)		*Firmicutes*	*Roseburia intestinalis*
TM25_12wk_k141_69979	1	dsDNAphage	Yes	High-quality	100	High-quality draft genome	AAI-based (medium-confidence)		*Firmicutes*	*Faecalibacterium prausnitzii*
TM25_26wk_k141_13191	1	dsDNAphage	Yes	High-quality	100	High-quality draft genome	AAI-based (high-confidence)	*Myoviridae*	*Proteobacteria*	*Escherichia coli*
TM25_6wk_k141_12323	0.993	dsDNAphage	Yes	High-quality	91.36	High-quality draft genome	AAI-based (high-confidence)		*Firmicutes*	*Coprococcus comes*
TM25_BL_k141_22837	0.993	dsDNAphage	Yes	High-quality	93.14	High-quality draft genome	AAI-based (high-confidence)		*Firmicutes*	*Faecalibacterium prausnitzii*
TM25_BL_k141_33361	0.993	dsDNAphage	Yes	High-quality	96.61	High-quality draft genome	AAI-based (high-confidence)		*Firmicutes*	*Tyzzerella nexilis*
TM25_BL_k141_70406	0.98	dsDNAphage	Yes	Complete	100	High-quality draft genome	Provirus (medium-confidence)	*Siphoviridae*	*Firmicutes*	*Faecalibacterium prausnitzii*
TM27_12wk_k141_69175	0.987	dsDNAphage	Yes	High-quality	100	High-quality draft genome	AAI-based (high-confidence)			
TM27_26wk_k141_73681	0.993	dsDNAphage	Yes	Complete	100	High-quality draft genome	Provirus (high-confidence)			
TM27_BL_k141_67452	0.987	dsDNAphage	Yes	High-quality	100	High-quality draft genome	AAI-based (high-confidence)	*Siphoviridae*	*Firmicutes*	*Faecalibacterium prausnitzii*
TM28_26wk_k141_169172	1	dsDNAphage	Yes	Complete	100	High-quality draft genome	Provirus (high-confidence)			
TM28_BL_k141_111121	0.987	dsDNAphage	Yes	High-quality	91.46	High-quality draft genome	AAI-based (high-confidence)		*Firmicutes*	*Roseburia inulinivorans*
TM29_12wk_k141_24600	0.993	dsDNAphage	Yes	High-quality	100	High-quality draft genome	AAI-based (high-confidence)		*Firmicutes*	*Dorea longicatena*
TM30_12wk_k141_108182	1	dsDNAphage	Yes	High-quality	94.53	High-quality draft genome	AAI-based (high-confidence)		*Firmicutes*	*Faecalibacterium prausnitzii*
TM30_26wk_k141_20748	1	dsDNAphage	Yes	High-quality	100	High-quality draft genome	AAI-based (high-confidence)			
TM32_12wk_k141_1648	1	dsDNAphage	Yes	High-quality	99.23	High-quality draft genome	AAI-based (high-confidence)	*Siphoviridae*	*Firmicutes*	*Faecalibacterium prausnitzii*
TM32_BL_k141_103800	0.98	dsDNAphage	Yes	High-quality	100	High-quality draft genome	AAI-based (medium-confidence)	*Myoviridae*		
TM32_BL_k141_9783	0.987	dsDNAphage	Yes	Complete	100	High-quality draft genome	Provirus (high-confidence)		*Firmicutes*	*Tyzzerella nexilis*
TM33_BL_k141_85036	1	dsDNAphage	Yes	High-quality	100	High-quality draft genome	AAI-based (high-confidence)		*Actinobacteria*	*Bifidobacterium longum*
TM34_6wk_k141_18050	1	dsDNAphage	Yes	High-quality	100	High-quality draft genome	AAI-based (high-confidence)			
TM34_BL_k141_148153	1	dsDNAphage	Yes	High-quality	100	High-quality draft genome	AAI-based (high-confidence)		*Firmicutes*	*Faecalibacterium prausnitzii*
TM34_BL_k141_39027	0.987	dsDNAphage	Yes	High-quality	100	High-quality draft genome	AAI-based (high-confidence)	*Myoviridae*	*Firmicutes*	*Roseburia inulinivorans*
TM35_BL_k141_100928	0.993	dsDNAphage	Yes	High-quality	95.38	High-quality draft genome	AAI-based (high-confidence)	*Myoviridae*		
TM35_BL_k141_148296	0.987	ssDNA	Yes	High-quality	100	High-quality draft genome	AAI-based (high-confidence)	*Circoviridae*		
TM35_BL_k141_228064	0.993	dsDNAphage	Yes	High-quality	100	High-quality draft genome	AAI-based (high-confidence)			
TM36_12wk_k141_56187	0.993	dsDNAphage	Yes	High-quality	93.3	High-quality draft genome	AAI-based (high-confidence)	*Myoviridae*	*Firmicutes*	*Roseburia intestinalis*
TM37_BL_k141_55499	1	dsDNAphage	Yes	High-quality	100	High-quality draft genome	AAI-based (high-confidence)			
TM39_26wk_k141_99362	1	dsDNAphage	Yes	High-quality	100	High-quality draft genome	AAI-based (high-confidence)			
TM39_6wk_k141_23816	0.987	dsDNAphage	Yes	Complete	100	High-quality draft genome	Provirus (high-confidence)	*Siphoviridae*	*Firmicutes*	*Faecalibacterium prausnitzii*
TM39_BL_k141_105211	1	dsDNAphage	Yes	High-quality	100	High-quality draft genome	AAI-based (high-confidence)			
TM13_12wk_k141_23202	0.993	dsDNAphage	No	Complete	100	High-quality draft genome	DTR (high-confidence)			
TM13_26wk_k141_21568	0.993	dsDNAphage	No	Complete	100	High-quality draft genome	DTR (high-confidence)			
TF26_12wk_k141_95061	1	dsDNAphage	No	High-quality	98.01	High-quality draft genome	AAI-based (high-confidence)			
DF16_D6_k141_363535	0.993	dsDNAphage	No	Complete	100	High-quality draft genome	DTR (high-confidence)			
TF14_6wk_k141_89992	1	dsDNAphage	No	Complete	100	High-quality draft genome	DTR (high-confidence)	*Crass_like*		
TM05_6wk_k141_6620	1	dsDNAphage	No	Complete	100	High-quality draft genome	DTR (high-confidence)			
TM05_26wk_k141_11993	1	dsDNAphage	No	Complete	100	High-quality draft genome	DTR (high-confidence)			
DM07_D2_k141_214701	1	dsDNAphage	No	Complete	100	High-quality draft genome	DTR (high-confidence)			
DM07_D5_k141_222374	1	dsDNAphage	No	Complete	100	High-quality draft genome	DTR (high-confidence)			
TF42_12wk_k141_118416	0.98	dsDNAphage	No	Complete	100	High-quality draft genome	DTR (high-confidence)		*Firmicutes*	*Anaerobutyricum hallii*
TF41_12wk_k141_109208	0.993	dsDNAphage	No	Complete	100	High-quality draft genome	DTR (high-confidence)	*Microviridae*		
DM03_D4_k141_146730	0.993	dsDNAphage	No	Complete	100	High-quality draft genome	DTR (high-confidence)			
TF12_6wk_k141_40773	1	dsDNAphage	No	Complete	100	High-quality draft genome	DTR (high-confidence)		*Firmicutes*	*Roseburia inulinivorans*
TF36_26wk_k141_105312	1	dsDNAphage	No	Complete	100	High-quality draft genome	DTR (high-confidence)	*Microviridae*		
DF16_D7_k141_330256	1	dsDNAphage	No	Complete	100	High-quality draft genome	DTR (high-confidence)			
DF16_D8_k141_307561	1	dsDNAphage	No	Complete	100	High-quality draft genome	DTR (high-confidence)	*Microviridae*		
DF16_D6_k141_363379	1	dsDNAphage	No	Complete	100	High-quality draft genome	DTR (high-confidence)	*Microviridae*		
DF16_D5_k141_340608	1	dsDNAphage	No	Complete	100	High-quality draft genome	DTR (high-confidence)	*Microviridae*		
DF16a_D4_k141_290409	1	dsDNAphage	No	Complete	100	High-quality draft genome	DTR (high-confidence)	*Microviridae*		
DF16_D3_k141_292651	1	dsDNAphage	No	Complete	100	High-quality draft genome	DTR (high-confidence)	*Microviridae*		
DF16_D2_k141_356115	1	dsDNAphage	No	Complete	100	High-quality draft genome	DTR (high-confidence)	*Microviridae*		
TF03_12wk_k141_128199	1	dsDNAphage	No	Complete	100	High-quality draft genome	DTR (high-confidence)			
DF12_D5_k141_120251	0.993	dsDNAphage	No	Complete	100	High-quality draft genome	DTR (high-confidence)			
DF12_D3_k141_107020	0.993	dsDNAphage	No	Complete	100	High-quality draft genome	DTR (high-confidence)			
DF12_D4_k141_10216	0.98	dsDNAphage	No	Complete	100	High-quality draft genome	DTR (high-confidence)			
DF12_D2_k141_109468	0.993	dsDNAphage	No	Complete	100	High-quality draft genome	DTR (high-confidence)			
DF12_D6_k141_104099	0.993	dsDNAphage	No	Complete	100	High-quality draft genome	DTR (high-confidence)			
TF48_12wk_k141_216464	1	dsDNAphage	No	Complete	100	High-quality draft genome	DTR (high-confidence)			
TM27_12wk_k141_84521	0.933	dsDNAphage	No	Complete	100	High-quality draft genome	DTR (high-confidence)			

UViG	VirSorter2 score	Predicted genome type	Provirus	Completeness quality	Completeness (%)	Assembly quality	Completeness approach	Family	Host phylum	Host species
TM07_12wk_k141_250564	0.993	dsDNAphage	No	Complete	100	High-quality draft genome	DTR (high-confidence)	*Microviridae*		
TM13_12wk_k141_98681	1	dsDNAphage	No	Complete	100	High-quality draft genome	DTR (high-confidence)			
TF27_6wk_k141_142122	0.993	dsDNAphage	Yes	High-quality	91.76	High-quality draft genome	AAI-based (high-confidence)			
DF16_D7_k141_330302	0.587	dsDNAphage	No	Complete	100	High-quality draft genome	DTR (medium-confidence)			
TF29_6WK_k141_204308	0.587	dsDNAphage	No	Complete	100	High-quality draft genome	DTR (medium-confidence)			
DF16_D5_k141_340650	0.56	dsDNAphage	No	Complete	100	High-quality draft genome	DTR (medium-confidence)			
TM18_6wk_k141_74364	0.98	dsDNAphage	No	Complete	100	High-quality draft genome	DTR (high-confidence)			
TM16_12wk_k141_254349	0.993	dsDNAphage	No	Complete	100	High-quality draft genome	DTR (high-confidence)			
TF49_12wk_k141_212026	1	dsDNAphage	No	Complete	100	High-quality draft genome	DTR (high-confidence)	*Microviridae*		
TF28_12wk_k141_46624	0.987	dsDNAphage	No	Complete	100	High-quality draft genome	DTR (high-confidence)		*Actinobacteria*	*Bifidobacterium longum*
TF28_26wk_k141_86575	0.987	dsDNAphage	No	Complete	100	High-quality draft genome	DTR (high-confidence)		*Actinobacteria*	*Bifidobacterium longum*
TF25_12wk_k141_97906	1	dsDNAphage	Yes	Complete	100	High-quality draft genome	Provirus (high-confidence)		*Firmicutes*	*Catenibacterium mitsuokai*
TF03_26wk_k141_54501	1	dsDNAphage	No	Complete	100	High-quality draft genome	DTR (high-confidence)			
TM28_12wk_k141_166975	1	dsDNAphage	No	Complete	100	High-quality draft genome	DTR (high-confidence)			
TF03_BL_k141_183968	1	dsDNAphage	No	Complete	100	High-quality draft genome	DTR (high-confidence)			
TF07_6wk_k141_50137	1	dsDNAphage	No	Complete	100	High-quality draft genome	DTR (high-confidence)			
TM33_12wk_k141_83144	1	dsDNAphage	No	Complete	100	High-quality draft genome	DTR (high-confidence)	*Microviridae*		
DM03_D4_k141_59668	1	dsDNAphage	No	Complete	100	High-quality draft genome	DTR (high-confidence)			
DM03_D3_k141_117151	1	dsDNAphage	No	Complete	100	High-quality draft genome	DTR (high-confidence)			
DM03_D1_k141_128571	1	dsDNAphage	No	Complete	100	High-quality draft genome	DTR (high-confidence)			
TF27_6wk_k141_28515	1	dsDNAphage	No	Complete	100	High-quality draft genome	DTR (high-confidence)			
DF16_D5_k141_191450	1	dsDNAphage	No	Complete	100	High-quality draft genome	DTR (high-confidence)			
DF16_D7_k141_11319	1	dsDNAphage	No	Complete	100	High-quality draft genome	DTR (high-confidence)	*Myoviridae*		
DF16_D3_k141_132500	1	dsDNAphage	No	Complete	100	High-quality draft genome	DTR (high-confidence)	*Myoviridae*		
DF16_D2_k141_355860	1	dsDNAphage	No	Complete	100	High-quality draft genome	DTR (high-confidence)			
DF14_D6_k141_163880	0.913	dsDNAphage	No	High-quality	100	High-quality draft genome	AAI-based (high-confidence)			
DF17_D4_k141_109549	0.913	dsDNAphage	No	High-quality	100	High-quality draft genome	AAI-based (high-confidence)			
DF14_D7_k141_177905	1	dsDNAphage	No	Complete	100	High-quality draft genome	DTR (high-confidence)	*Microviridae*		
DF17_D6_k141_159669	0.913	dsDNAphage	No	High-quality	100	High-quality draft genome	AAI-based (high-confidence)			
TF15_12wk_k141_105118	0.993	dsDNAphage	No	Complete	100	High-quality draft genome	DTR (high-confidence)		*Firmicutes*	*Blautia obeum*
TM28_26wk_k141_91299	1	dsDNAphage	No	Complete	100	High-quality draft genome	DTR (high-confidence)			
TF01_26wk_k141_80283	0.993	dsDNAphage	No	High-quality	100	High-quality draft genome	AAI-based (high-confidence)	*Microviridae*		
TF18_12wk_k141_156362	0.5	dsDNAphage	No	Complete	100	High-quality draft genome	DTR (high-confidence)			
TM24_6wk_k141_111847	0.993	dsDNAphage	No	Complete	100	High-quality draft genome	DTR (high-confidence)			
TM24_26wk_k141_162516	0.993	dsDNAphage	No	Complete	100	High-quality draft genome	DTR (high-confidence)			
TM09_12wk_k141_51901	1	dsDNAphage	No	Complete	100	High-quality draft genome	DTR (high-confidence)	*Crass_like*	*Firmicutes*	*Staphylococcus aureus*
DF17_D9_k141_67828	0.94	dsDNAphage	No	High-quality	100	High-quality draft genome	AAI-based (high-confidence)			
TM05_26wk_k141_95403	1	dsDNAphage	No	High-quality	99.4	High-quality draft genome	AAI-based (high-confidence)			
TF38_12wk_k141_60436	1	dsDNAphage	No	Complete	100	High-quality draft genome	DTR (high-confidence)			
DF16_D6_k141_353683	0.953	dsDNAphage	No	High-quality	100	High-quality draft genome	AAI-based (high-confidence)			
TM08_6wk_k141_183819	0.993	dsDNAphage	No	Complete	100	High-quality draft genome	DTR (high-confidence)		*Firmicutes*	*Roseburia intestinalis*
TF01_6wk_k141_156584	0.913	dsDNAphage	No	High-quality	100	High-quality draft genome	AAI-based (high-confidence)			
DF17_D7_k141_136533	0.913	dsDNAphage	No	High-quality	100	High-quality draft genome	AAI-based (high-confidence)			
DF14_D2_k141_165816	1	dsDNAphage	No	Complete	100	High-quality draft genome	DTR (high-confidence)			
TF16_12wk_k141_30168	0.993	dsDNAphage	No	Complete	100	High-quality draft genome	DTR (high-confidence)			
DF12_D3_k141_106946	1	dsDNAphage	No	Complete	100	High-quality draft genome	DTR (high-confidence)			
TM15_6wk_k141_779	1	dsDNAphage	No	Complete	100	High-quality draft genome	DTR (high-confidence)		*Firmicutes*	*Blautia obeum*
TF15_26wk_k141_58107	0.993	dsDNAphage	No	Complete	100	High-quality draft genome	DTR (high-confidence)		*Firmicutes*	*Blautia obeum*
TM27_26wk_k141_143177	1	dsDNAphage	No	Complete	100	High-quality draft genome	DTR (high-confidence)			
TF41_6wk_k141_209036	0.973	dsDNAphage	No	Complete	100	High-quality draft genome	DTR (high-confidence)			
TF41_BL_k141_183067	0.973	dsDNAphage	No	Complete	100	High-quality draft genome	DTR (high-confidence)			
TF30_26wk_k141_136064	0.987	dsDNAphage	No	Complete	100	High-quality draft genome	DTR (high-confidence)		*Firmicutes*	*Faecalibacterium prausnitzii*
DM07_D3_k141_218716	0.993	dsDNAphage	No	Complete	100	High-quality draft genome	DTR (high-confidence)			
DF12_D2_k141_71232	0.993	dsDNAphage	No	Complete	100	High-quality draft genome	DTR (high-confidence)			
DF12_D4_k141_52149	0.993	dsDNAphage	No	Complete	100	High-quality draft genome	DTR (high-confidence)			
TM28_6wk_k141_74786	1	dsDNAphage	No	Complete	100	High-quality draft genome	DTR (high-confidence)			
TM28_26wk_k141_18300[illegible]	1	dsDNAphage	No	Complete	100	High-quality draft genome	DTR (high-confidence)			
DF16_D8_k141_307179	0.993	dsDNAphage	No	Complete	100	High-quality draft genome	DTR (high-confidence)			
TM17_26wk_k141_153535	1	dsDNAphage	No	Complete	100	High-quality draft genome	DTR (high-confidence)	*Microviridae*		
TF34_12wk_k141_15495	1	ssDNA	No	Complete	100	High-quality draft genome	DTR (high-confidence)	*Microviridae*		
TF02_6wk_k141_11103	0.993	dsDNAphage	No	High-quality	97.61	High-quality draft genome	AAI-based (high-confidence)			
DF12_D8_k141_68431	1	dsDNAphage	No	Complete	100	High-quality draft genome	DTR (high-confidence)			
TF25_26wk_k141_24427	0.98	dsDNAphage	No	Complete	100	High-quality draft genome	DTR (high-confidence)			
TF28_6wk_k141_61207	0.987	dsDNAphage	No	Complete	100	High-quality draft genome	DTR (high-confidence)		*Actinobacteria*	*Bifidobacterium longum*
DF12_D4_k141_66543	1	dsDNAphage	Yes	High-quality	95.23	High-quality draft genome	AAI-based (high-confidence)			
DF12_D3_k141_37475	0.987	dsDNAphage	Yes	High-quality	95.23	High-quality draft genome	AAI-based (high-confidence)			
TM06_6wk_k141_139838	0.967	dsDNAphage	No	Complete	100	High-quality draft genome	DTR (high-confidence)			
TF12_26wk_k141_41279	0.98	dsDNAphage	No	Complete	100	High-quality draft genome	DTR (high-confidence)			
DF12_D5_k141_75317	0.993	dsDNAphage	No	High-quality	100	High-quality draft genome	AAI-based (high-confidence)	*Siphoviridae*		
TF30_12wk_k141_161613	0.913	dsDNAphage	No	High-quality	100	High-quality draft genome	AAI-based (high-confidence)			
TF29_26wk_k141_173757	0.913	dsDNAphage	No	High-quality	100	High-quality draft genome	AAI-based (high-confidence)			
TF29_12wk_k141_189927	0.913	dsDNAphage	No	High-quality	100	High-quality draft genome	AAI-based (high-confidence)			
TF50_26wk_k141_201912	0.78	dsDNAphage	No	Complete	100	High-quality draft genome	DTR (high-confidence)			
TF20_12wk_k141_18491	0.98	dsDNAphage	No	Complete	100	High-quality draft genome	DTR (high-confidence)			
TF02_12wk_k141_241533	1	dsDNAphage	No	Complete	100	High-quality draft genome	DTR (high-confidence)	*Microviridae*		
TM25_6wk_k141_132671	1	dsDNAphage	No	Complete	100	High-quality draft genome	DTR (high-confidence)		*Firmicutes*	*Blautia obeum*
TF29_12wk_k141_134741	1	dsDNAphage	No	Complete	100	High-quality draft genome	DTR (high-confidence)		*Firmicutes*	*Blautia obeum*
TF39_6wk_k141_78000	0.987	dsDNAphage	No	Complete	100	High-quality draft genome	DTR (high-confidence)		*Firmicutes*	*Faecalibacterium prausnitzii*
TF39_26wk_k141_97296	0.987	dsDNAphage	No	Complete	100	High-quality draft genome	DTR (high-confidence)		*Firmicutes*	*Faecalibacterium prausnitzii*
TM27_6wk_k141_182960	0.993	dsDNAphage	No	Complete	100	High-quality draft genome	DTR (high-confidence)		*Firmicutes*	*Faecalibacterium prausnitzii*
TF48_12wk_k141_216544	0.993	dsDNAphage	No	Complete	100	High-quality draft genome	DTR (high-confidence)		*Firmicutes*	*Faecalibacterium prausnitzii*
TF48_26wk_k141_239515	1	dsDNAphage	No	Complete	100	High-quality draft genome	DTR (high-confidence)		*Firmicutes*	*Faecalibacterium prausnitzii*
DF12_D5_k141_120229	0.993	dsDNAphage	No	Complete	100	High-quality draft genome	DTR (high-confidence)			
TF39_26wk_k141_80391	1	dsDNAphage	No	Complete	100	High-quality draft genome	DTR (high-confidence)			
DF14_D4_k141_58199	1	dsDNAphage	No	Complete	100	High-quality draft genome	DTR (high-confidence)			
TF29_26wk_k141_214396	1	dsDNAphage	No	Complete	100	High-quality draft genome	DTR (high-confidence)		*Firmicutes*	*Blautia obeum*
DM07_D5_k141_111780	1	dsDNAphage	No	Complete	100	High-quality draft genome	DTR (high-confidence)			
TF26_26wk_k141_103398	1	dsDNAphage	No	High-quality	98.01	High-quality draft genome	AAI-based (high-confidence)			
DM07_D2_k141_214782	1	dsDNAphage	No	Complete	100	High-quality draft genome	DTR (high-confidence)			
TF36_12wk_k141_144092	0.913	dsDNAphage	No	High-quality	100	High-quality draft genome	AAI-based (high-confidence)			
TF36_6wk_k141_208763	0.913	dsDNAphage	No	High-quality	100	High-quality draft genome	AAI-based (high-confidence)			
TF23_6wk_k141_84139	0.913	dsDNAphage	No	High-quality	100	High-quality draft genome	AAI-based (high-confidence)			
TF19_26wk_k141_222089	1	dsDNAphage	No	Complete	100	High-quality draft genome	DTR (high-confidence)	*Microviridae*		
DF16_D8_k141_262997	1	dsDNAphage	No	Complete	100	High-quality draft genome	DTR (high-confidence)	*Myoviridae*		
TF28_12wk_k141_170284	0.993	dsDNAphage	No	Complete	100	High-quality draft genome	DTR (high-confidence)		*Bacteroidetes*	*Prevotella copri*
TF03_26wk_k141_272766	0.987	dsDNAphage	No	Complete	100	High-quality draft genome	DTR (high-confidence)			
TF25_12wk_k141_133733	1	dsDNAphage	No	Complete	100	High-quality draft genome	DTR (high-confidence)	*Microviridae*		
TF39_26wk_k141_257019	0.987	dsDNAphage	No	Complete	100	High-quality draft genome	DTR (high-confidence)			
TF10_6wk_k141_30673	0.993	dsDNAphage	No	Complete	100	High-quality draft genome	DTR (high-confidence)	*Crass_like*		
DM07_D4_k141_217314	1	dsDNAphage	No	Complete	100	High-quality draft genome	DTR (high-confidence)			
TF20_6WK_k141_239691	0.98	dsDNAphage	No	Complete	100	High-quality draft genome	DTR (high-confidence)			
TM24_26wk_k141_8803	0.993	dsDNAphage	No	Complete	100	High-quality draft genome	DTR (high-confidence)		*Firmicutes*	*Faecalibacterium prausnitzii*
TF42_26wk_k141_72286	0.98	dsDNAphage	No	Complete	100	High-quality draft genome	DTR (high-confidence)		*Firmicutes*	*Anaerobutyricum hallii*
TF48_6wk_k141_128410	0.993	dsDNAphage	No	Complete	100	High-quality draft genome	DTR (high-confidence)		*Firmicutes*	*Faecalibacterium prausnitzii*
DF14_D2_k141_166028	1	dsDNAphage	No	Complete	100	High-quality draft genome	DTR (high-confidence)	*Microviridae*		

UViG	VirSorter2 score	Predicted genome type	Provirus	Completeness quality	Completeness (%)	Assembly quality	Completeness approach	Family	Host phylum	Host species
TF25_26wk_k141_121241	1	dsDNAphage	Yes	Complete	100	High-quality draft genome	Provirus (high-confidence)		*Firmicutes*	*Catenibacterium mitsuokai*
TF51_12wk_k141_58032	0.993	dsDNAphage	Yes	High-quality	92.91	High-quality draft genome	AAI-based (high-confidence)	*Siphoviridae*	*Firmicutes*	*Faecalibacterium prausnitzii*
TM06_6wk_k141_40054	1	dsDNAphage	Yes	High-quality	91.55	High-quality draft genome	AAI-based (high-confidence)		*Firmicutes*	*Faecalibacterium prausnitzii*
TM39_BL_k141_164480	0.993	dsDNAphage	No	High-quality	91.16	High-quality draft genome	AAI-based (high-confidence)			
TM37_BL_k141_27034	1	dsDNAphage	No	High-quality	99.93	High-quality draft genome	AAI-based (high-confidence)			
TM37_BL_k141_30158	1	dsDNAphage	No	High-quality	98.05	High-quality draft genome	AAI-based (high-confidence)		*Firmicutes*	*Dorea formicigenerans*
TM37_BL_k141_43650	0.993	dsDNAphage	No	High-quality	100	High-quality draft genome	AAI-based (high-confidence)			
TM37_BL_k141_120569	0.98	dsDNAphage	No	High-quality	100	High-quality draft genome	AAI-based (high-confidence)			
TM35_BL_k141_100454	0.993	dsDNAphage	No	High-quality	91.04	High-quality draft genome	AAI-based (high-confidence)			
TM34_BL_k141_103427	0.773	dsDNAphage	No	High-quality	96.64	High-quality draft genome	AAI-based (high-confidence)	*Circoviridae*		
TM34_26wk_k141_18996	0.98	ssDNA	No	High-quality	100	High-quality draft genome	AAI-based (high-confidence)			
TM32_6WK_k141_22930	0.993	dsDNAphage	No	High-quality	100	High-quality draft genome	AAI-based (high-confidence)	*Podoviridae*		
TM32_26wk_k141_31249	0.993	dsDNAphage	No	High-quality	98.12	High-quality draft genome	AAI-based (high-confidence)	*Crass_like*		
TM32_12wk_k141_72721	0.973	ssDNA	No	High-quality	99.32	High-quality draft genome	HMM-based (lower-bound)			
TM30_6wk_k141_21578	0.967	ssDNA	No	High-quality	100	High-quality draft genome	AAI-based (high-confidence)			
TM28_26wk_k141_92496	0.613	dsDNAphage	No	Complete	100	High-quality draft genome	DTR (high-confidence)			
TM25_26wk_k141_26515	0.533	dsDNAphage	No	High-quality	100	High-quality draft genome	HMM-based (lower-bound)		*Firmicutes*	*Tyzzerella nexilis*
TM25_26wk_k141_10967	0.92	dsDNAphage	No	High-quality	100	High-quality draft genome	AAI-based (medium-confidence)	*Microviridae*	*Bacteroidetes*	*Bacteroides finegoldii*
TM25_26wk_k141_15960	1	dsDNAphage	No	Complete	100	High-quality draft genome	DTR (high-confidence)	*Microviridae*		
TM25_26wk_k141_15980	1	dsDNAphage	No	Complete	100	High-quality draft genome	DTR (high-confidence)	*Microviridae*		
TM24_BL_k141_56009	0.947	dsDNAphage	No	High-quality	97.48	High-quality draft genome	AAI-based (medium-confidence)			
TM24_BL_k141_154851	1	dsDNAphage	No	High-quality	98.02	High-quality draft genome	AAI-based (high-confidence)			
TM24_26wk_k141_45193	1	dsDNAphage	No	High-quality	97.73	High-quality draft genome	AAI-based (high-confidence)			
TM24_26wk_k141_52951	1	dsDNAphage	No	High-quality	96.93	High-quality draft genome	AAI-based (high-confidence)	*Microviridae*		
TM24_26wk_k141_89335	1	dsDNAphage	No	High-quality	93.75	High-quality draft genome	AAI-based (high-confidence)	*Microviridae*		
TM24_26wk_k141_16245	1	dsDNAphage	No	Complete	100	High-quality draft genome	DTR (high-confidence)			
TM23_BL_k141_8924	1	dsDNAphage	No	High-quality	99.38	High-quality draft genome	AAI-based (high-confidence)	*Microviridae*		
TM23_BL_k141_91299	1	dsDNAphage	No	Complete	100	High-quality draft genome	DTR (high-confidence)	*Microviridae*		
TM23_6wk_k141_75867	1	dsDNAphage	No	High-quality	97.96	High-quality draft genome	AAI-based (high-confidence)			
TM22_BL_k141_81824	1	ssDNA	No	High-quality	94.51	High-quality draft genome	AAI-based (medium-confidence)			
TM22_6wk_k141_23618	0.947	dsDNAphage	No	High-quality	93.83	High-quality draft genome	AAI-based (high-confidence)			
TM22_6wk_k141_33082	0.973	dsDNAphage	No	High-quality	100	High-quality draft genome	AAI-based (high-confidence)	*Microviridae*		
TM22_6wk_k141_67646	1	dsDNAphage	No	High-quality	93.59	High-quality draft genome	AAI-based (high-confidence)			
TM22_6wk_k141_88888	1	dsDNAphage	No	High-quality	97.2	High-quality draft genome	AAI-based (high-confidence)			
TM22_26wk_k141_12379	1	ssDNA	No	Complete	100	High-quality draft genome	DTR (high-confidence)	*Microviridae*	*Bacteroidetes*	*Prevotella disiens*
TM21_6wk_k141_137784	1	dsDNAphage	No	High-quality	100	High-quality draft genome	AAI-based (high-confidence)		*Actinobacteria*	*Bifidobacterium bifidum*
TM19_BL_k141_96262	0.993	dsDNAphage	No	High-quality	98.94	High-quality draft genome	AAI-based (high-confidence)	*Microviridae*		
TM19_BL_k141_102104	0.993	ssDNA	No	High-quality	100	High-quality draft genome	AAI-based (high-confidence)	*Microviridae*		
TM19_BL_k141_154505	1	dsDNAphage	No	Complete	100	High-quality draft genome	DTR (high-confidence)			
TM17_6WK_k141_126947	0.953	dsDNAphage	No	High-quality	98.56	High-quality draft genome	AAI-based (high-confidence)			
TM17_26wk_k141_24353	1	dsDNAphage	No	High-quality	100	High-quality draft genome	AAI-based (high-confidence)			
TM17_26wk_k141_67207	1	dsDNAphage	No	High-quality	100	High-quality draft genome	AAI-based (high-confidence)		*Firmicutes*	*Intestinibacter bartlettii*
TM17_26wk_k141_31210	0.993	ssDNA	No	Complete	100	High-quality draft genome	DTR (high-confidence)	*Microviridae*		
TM17_26wk_k141_153615	1	dsDNAphage	No	Complete	100	High-quality draft genome	DTR (high-confidence)	*Microviridae*		
TM16_BL_k141_184790	0.9	dsDNAphage	No	High-quality	100	High-quality draft genome	AAI-based (high-confidence)			
TM16_26wk_k141_126584	1	dsDNAphage	No	High-quality	99.39	High-quality draft genome	AAI-based (high-confidence)			
TM16_12wk_k141_171603	0.993	dsDNAphage	No	High-quality	100	High-quality draft genome	AAI-based (high-confidence)		*Firmicutes*	*Staphylococcus epidermidis*
TM16_12wk_k141_30993	0.993	dsDNAphage	No	Complete	100	High-quality draft genome	DTR (high-confidence)			
TM16_12wk_k141_254319	0.787	dsDNAphage	No	Complete	100	High-quality draft genome	DTR (high-confidence)			
TM15_BL_k141_121071	0.933	dsDNAphage	No	Complete	100	High-quality draft genome	DTR (high-confidence)			
TM15_6wk_k141_66091	1	dsDNAphage	No	High-quality	92.76	High-quality draft genome	AAI-based (high-confidence)			
TM15_6wk_k141_96473	1	dsDNAphage	No	High-quality	100	High-quality draft genome	AAI-based (high-confidence)	*Microviridae*		
TM14_BL_k141_145828	1	dsDNAphage	No	High-quality	91.03	High-quality draft genome	AAI-based (high-confidence)			
TM14_BL_k141_91088	0.993	dsDNAphage	No	Complete	100	High-quality draft genome	DTR (high-confidence)			
TM14_6wk_k141_32965	0.993	dsDNAphage	No	High-quality	100	High-quality draft genome	AAI-based (high-confidence)			
TM14_26wk_k141_46317	0.9	ssDNA	No	High-quality	100	High-quality draft genome	HMM-based (lower-bound)			
TM13_12wk_k141_231815	1	dsDNAphage	No	Complete	100	High-quality draft genome	DTR (high-confidence)	*Podoviridae*		
TM12_6WK_k141_120422	0.993	dsDNAphage	No	Complete	100	High-quality draft genome	ITR (high-confidence)	*Podoviridae*	*Bacteroidetes*	*Prevotella disiens*
TM11_BL_k141_898	0.92	ssDNA	No	High-quality	100	High-quality draft genome	AAI-based (high-confidence)			
TM11_BL_k141_10988	1	dsDNAphage	No	High-quality	100	High-quality draft genome	AAI-based (high-confidence)	*Microviridae*		
TM11_BL_k141_72415	1	dsDNAphage	No	Complete	100	High-quality draft genome	DTR (high-confidence)	*Siphoviridae*		
TM11_BL_k141_113598	0.987	dsDNAphage	No	Complete	100	High-quality draft genome	DTR (high-confidence)	*Podoviridae*		
TM10_BL_k141_75400	1	dsDNAphage	No	Complete	100	High-quality draft genome	DTR (high-confidence)	*Microviridae*		
TM10_6wk_k141_2525	1	dsDNAphage	No	Complete	100	High-quality draft genome	DTR (high-confidence)			
TM09_12wk_k141_87196	0.867	dsDNAphage	No	High-quality	100	High-quality draft genome	AAI-based (medium-confidence)			
TM08_6wk_k141_130099	0.94	ssDNA	No	High-quality	100	High-quality draft genome	HMM-based (lower-bound)			
TM08_26wk_k141_18924	1	dsDNAphage	No	High-quality	100	High-quality draft genome	AAI-based (high-confidence)		*Firmicutes*	*Dialister invisus*
TM08_26wk_k141_23853	1	dsDNAphage	No	Complete	100	High-quality draft genome	DTR (high-confidence)			
TM07_26wk_k141_98131	0.987	dsDNAphage	No	High-quality	98.57	High-quality draft genome	AAI-based (medium-confidence)	*Myoviridae*	*Firmicutes*	*Blautia obeum*
TM07_26wk_k141_54199	0.993	dsDNAphage	No	Complete	100	High-quality draft genome	DTR (high-confidence)		*Firmicutes*	*Lactobacillus paracasei*
TM07_12wk_k141_14068	0.98	dsDNAphage	No	High-quality	100	High-quality draft genome	AAI-based (high-confidence)			
TM06_BL_k141_167295	1	dsDNAphage	No	High-quality	100	High-quality draft genome	AAI-based (medium-confidence)			
TM06_6wk_k141_141348	0.993	dsDNAphage	No	High-quality	93.57	High-quality draft genome	AAI-based (high-confidence)	*Siphoviridae*	*Firmicutes*	*Anaerobutyricum hallii*
TM05_BL_k141_226998	1	dsDNAphage	No	Complete	100	High-quality draft genome	DTR (high-confidence)			
TM05_BL_k141_227004	1	dsDNAphage	No	Complete	100	High-quality draft genome	DTR (high-confidence)			
TM05_12wk_k141_13307	1	dsDNAphage	No	High-quality	100	High-quality draft genome	AAI-based (medium-confidence)	*Podoviridae*		
TM05_12wk_k141_162119	0.747	dsDNAphage	No	High-quality	100	High-quality draft genome	HMM-based (lower-bound)	*Circoviridae*		
TM04_BL_k141_166734	0.987	dsDNAphage	No	High-quality	100	High-quality draft genome	AAI-based (high-confidence)			
TM04_12wk_k141_25554	1	dsDNAphage	No	High-quality	97.82	High-quality draft genome	AAI-based (high-confidence)	*Podoviridae*		
TM02_6WK_k141_87411	1	dsDNAphage	No	High-quality	91.7	High-quality draft genome	AAI-based (medium-confidence)			
TM01_BL_k141_12466	0.993	dsDNAphage	No	High-quality	91.77	High-quality draft genome	AAI-based (medium-confidence)			
TF51_12wk_k141_13075	0.993	dsDNAphage	No	High-quality	99.47	High-quality draft genome	AAI-based (high-confidence)			
TF51_12wk_k141_115105	0.993	dsDNAphage	No	Complete	100	High-quality draft genome	DTR (high-confidence)			
TF50_6wk_k141_4396	0.993	dsDNAphage	No	High-quality	100	High-quality draft genome	AAI-based (high-confidence)			
TF50_6wk_k141_36784	0.88	ssDNA	No	High-quality	100	High-quality draft genome	HMM-based (lower-bound)			
TF50_26wk_k141_77055	0.907	dsDNAphage	No	High-quality	100	High-quality draft genome	HMM-based (lower-bound)			
TF50_12wk_k141_167888	1	dsDNAphage	No	High-quality	100	High-quality draft genome	AAI-based (high-confidence)	*Podoviridae*		
TF49_BL_k141_704	0.993	dsDNAphage	No	High-quality	99.91	High-quality draft genome	AAI-based (high-confidence)	*Myoviridae*		
TF49_BL_k141_9149	0.913	ssDNA	No	High-quality	98.62	High-quality draft genome	HMM-based (lower-bound)			
TF49_BL_k141_163529	0.987	dsDNAphage	No	Complete	100	High-quality draft genome	DTR (medium-confidence)			
TF49_6wk_k141_210838	0.94	dsDNAphage	No	High-quality	100	High-quality draft genome	AAI-based (medium-confidence)		*Firmicutes*	*Blautia obeum*
TF49_26wk_k141_214480	1	dsDNAphage	No	High-quality	100	High-quality draft genome	AAI-based (high-confidence)		*Firmicutes*	*Dialister invisus*
TF49_26wk_k141_139971	0.987	dsDNAphage	No	Complete	100	High-quality draft genome	DTR (high-confidence)			
TF48_BL_k141_16267	1	dsDNAphage	No	High-quality	93.92	High-quality draft genome	AAI-based (high-confidence)		*Firmicutes*	*Intestinibacter bartlettii*
TF48_BL_k141_65679	0.993	dsDNAphage	No	High-quality	95.11	High-quality draft genome	AAI-based (high-confidence)	*Microviridae*		
TF48_BL_k141_13497	1	dsDNAphage	No	Complete	100	High-quality draft genome	DTR (high-confidence)	*Microviridae*		
TF48_26wk_k141_23935	1	dsDNAphage	No	Complete	100	High-quality draft genome	DTR (high-confidence)			
TF47_BL_k141_13475	0.993	dsDNAphage	No	Complete	100	High-quality draft genome	DTR (high-confidence)	*Microviridae*		
TF47_BL_k141_180785	1	dsDNAphage	No	Complete	100	High-quality draft genome	DTR (high-confidence)	*Microviridae*		
TF47_12wk_k141_81813	0.767	ssDNA	No	High-quality	99.97	High-quality draft genome	HMM-based (lower-bound)			
TF46_BL_k141_17880	1	dsDNAphage	No	High-quality	95.89	High-quality draft genome	AAI-based (high-confidence)	*Microviridae*		
TF46_BL_k141_110724	1	dsDNAphage	No	Complete	100	High-quality draft genome	DTR (high-confidence)			
TF45_BL_k141_138304	1	dsDNAphage	No	Complete	100	High-quality draft genome	DTR (high-confidence)	*Microviridae*		
TF45_6wk_k141_136558	0.82	dsDNAphage	No	High-quality	100	High-quality draft genome	HMM-based (lower-bound)			
TF45_26wk_k141_25561	0.987	dsDNAphage	No	High-quality	100	High-quality draft genome	AAI-based (high-confidence)	*Microviridae*		

UViG	VirSorter2 score	Predicted genome type	Provirus	Completeness quality	Completeness (%)	Assembly quality	Completeness approach	Family	Host phylum	Host species
TF44_BL_k141_38566	0.627	dsDNAphage	No	High-quality	100	High-quality draft genome	HMM-based (lower-bound)	*Circoviridae*		
TF44_6wk_k141_107989	0.98	dsDNAphage	No	High-quality	93.03	High-quality draft genome	AAI-based (high-confidence)		*Firmicutes*	*Faecalibacterium prausnitzii*
TF42_BL_k141_62593	1	dsDNAphage	No	High-quality	100	High-quality draft genome	AAI-based (high-confidence)	*Microviridae*		
TF42_26wk_k141_114658	1	dsDNAphage	No	High-quality	100	High-quality draft genome	AAI-based (high-confidence)		*Firmicutes*	*Filifactor alocis*
TF41_BL_k141_26780	0.987	dsDNAphage	No	High-quality	91.41	High-quality draft genome	AAI-based (medium-confidence)			
TF41_6wk_k141_126188	1	dsDNAphage	No	High-quality	99.13	High-quality draft genome	AAI-based (high-confidence)	*Microviridae*		
TF41_26wk_k141_179052	1	dsDNAphage	No	Complete	100	High-quality draft genome	DTR (high-confidence)	*Microviridae*		
TF41_26wk_k141_120233	0.967	ssDNA	No	High-quality	100	High-quality draft genome	HMM-based (lower-bound)			
TF40_BL_k141_78896	0.9	ssDNA	No	High-quality	100	High-quality draft genome	HMM-based (lower-bound)	*Circoviridae*		
TF40_12wk_k141_74375	1	dsDNAphage	No	High-quality	96.7	High-quality draft genome	AAI-based (high-confidence)	*Siphoviridae*		
TF39_BL_k141_31829	0.773	dsDNAphage	No	High-quality	100	High-quality draft genome	AAI-based (high-confidence)			
TF38_BL_k141_14896	1	dsDNAphage	No	High-quality	92.97	High-quality draft genome	AAI-based (high-confidence)	*Microviridae*		
TF38_BL_k141_43564	1	dsDNAphage	No	High-quality	100	High-quality draft genome	AAI-based (high-confidence)			
TF38_26wk_k141_26497	0.873	dsDNAphage	No	High-quality	100	High-quality draft genome	AAI-based (high-confidence)			
TF38_26wk_k141_28908	0.993	dsDNAphage	No	Complete	100	High-quality draft genome	DTR (high-confidence)	*Microviridae*	*Bacteroidetes*	*Prevotella melaninogenica*
TF37_BL_k141_4890	0.947	dsDNAphage	No	High-quality	100	High-quality draft genome	HMM-based (lower-bound)			
TF37_6wk_k141_64363	0.987	dsDNAphage	No	High-quality	100	High-quality draft genome	AAI-based (high-confidence)			
TF37_6wk_k141_73459	0.987	dsDNAphage	No	High-quality	100	High-quality draft genome	AAI-based (high-confidence)			
TF37_6wk_k141_147751	1	dsDNAphage	No	Complete	100	High-quality draft genome	DTR (high-confidence)			
TF37_26wk_k141_101200	1	dsDNAphage	No	High-quality	97.63	High-quality draft genome	AAI-based (high-confidence)			
TF37_26wk_k141_13701	0.993	dsDNAphage	No	Complete	100	High-quality draft genome	DTR (high-confidence)	*Crass_like*		
TF37_26wk_k141_58524	0.993	dsDNAphage	No	Complete	100	High-quality draft genome	DTR (high-confidence)	*Microviridae*		
TF37_12wk_k141_18942	0.993	dsDNAphage	No	High-quality	100	High-quality draft genome	AAI-based (high-confidence)			
TF36_BL_k141_107271	0.967	dsDNAphage	No	High-quality	100	High-quality draft genome	AAI-based (medium-confidence)	*Myoviridae*		
TF36_BL_k141_70518	0.947	ssDNA	No	High-quality	100	High-quality draft genome	HMM-based (lower-bound)			
TF35_12wk_k141_213321	0.973	ssDNA	No	Complete	100	High-quality draft genome	DTR (high-confidence)			
TF34_26wk_k141_21747	1	dsDNAphage	No	High-quality	100	High-quality draft genome	AAI-based (high-confidence)		*Actinobacteria*	*Bifidobacterium bifidum*
TF33_BL_k141_60141	1	dsDNAphage	No	High-quality	100	High-quality draft genome	AAI-based (high-confidence)	*Myoviridae*	*Proteobacteria*	*Escherichia coli*
TF33_6wk_k141_63824	0.96	dsDNAphage	No	Complete	100	High-quality draft genome	ITR (high-confidence)	*Myoviridae*	*Proteobacteria*	*Escherichia coli*
TF33_6wk_k141_89482	0.987	dsDNAphage	No	High-quality	95.24	High-quality draft genome	AAI-based (high-confidence)	*Microviridae*	*Bacteroidetes*	*Prevotella bivia*
TF33_6wk_k141_118252	0.993	dsDNAphage	No	High-quality	93.16	High-quality draft genome	AAI-based (high-confidence)	*Microviridae*		
TF32_6wk_k141_17627	1	dsDNAphage	No	High-quality	90.23	High-quality draft genome	AAI-based (high-confidence)		*Firmicutes*	*Faecalibacterium prausnitzii*
TF32_6wk_k141_84476	0.88	ssDNA	No	High-quality	100	High-quality draft genome	HMM-based (lower-bound)			
TF32_26wk_k141_64677	1	dsDNAphage	No	High-quality	99.73	High-quality draft genome	AAI-based (high-confidence)	*Microviridae*		
TF32_26wk_k141_69232	1	dsDNAphage	No	High-quality	90.15	High-quality draft genome	AAI-based (high-confidence)		*Firmicutes*	*Faecalibacterium prausnitzii*
TF32_26wk_k141_83340	1	dsDNAphage	No	High-quality	96.42	High-quality draft genome	AAI-based (high-confidence)			
TF31_26wk_k141_103853	0.593	dsDNAphage	No	High-quality	100	High-quality draft genome	AAI-based (high-confidence)			
TF31_12wk_k141_146830	1	dsDNAphage	No	Complete	100	High-quality draft genome	DTR (high-confidence)			
TF31_12wk_k141_146868	1	dsDNAphage	No	Complete	100	High-quality draft genome	DTR (high-confidence)			
TF30_BL_k141_145842	1	dsDNAphage	No	High-quality	100	High-quality draft genome	AAI-based (high-confidence)			
TF30_26wk_k141_66901	0.807	ssDNA	No	High-quality	100	High-quality draft genome	HMM-based (lower-bound)			
TF30_12wk_k141_22187	0.98	dsDNAphage	No	High-quality	96.91	High-quality draft genome	AAI-based (high-confidence)		*Firmicutes*	*Faecalibacterium prausnitzii*
TF28_12wk_k141_2575	1	dsDNAphage	No	High-quality	98.37	High-quality draft genome	AAI-based (high-confidence)	*Siphoviridae*	*Firmicutes*	*Streptococcus gallolyticus*
TF28_12wk_k141_137855	0.993	ssDNA	No	Complete	100	High-quality draft genome	DTR (high-confidence)			
TF27_BL_k141_206392	0.933	ssDNA	No	High-quality	100	High-quality draft genome	HMM-based (lower-bound)	*Circoviridae*		
TF27_6wk_k141_132810	1	dsDNAphage	No	High-quality	100	High-quality draft genome	AAI-based (high-confidence)			
TF27_6wk_k141_211595	0.973	dsDNAphage	No	High-quality	100	High-quality draft genome	AAI-based (high-confidence)	*Microviridae*		
TF27_6wk_k141_222623	1	dsDNAphage	No	Complete	100	High-quality draft genome	DTR (high-confidence)	*Microviridae*	*Bacteroidetes*	*Prevotella disiens*
TF27_6wk_k141_153318	0.967	ssDNA	No	High-quality	100	High-quality draft genome	HMM-based (lower-bound)			
TF27_26wk_k141_10767	0.9	dsDNAphage	No	High-quality	100	High-quality draft genome	AAI-based (high-confidence)			
TF27_26wk_k141_193710	0.853	dsDNAphage	No	High-quality	99.98	High-quality draft genome	AAI-based (high-confidence)			
TF27_12wk_k141_84208	0.9	ssDNA	No	High-quality	100	High-quality draft genome	HMM-based (lower-bound)			
TF26_6wk_k141_32366	0.993	dsDNAphage	No	High-quality	100	High-quality draft genome	AAI-based (high-confidence)			
TF26_26wk_k141_122718	1	dsDNAphage	No	High-quality	100	High-quality draft genome	AAI-based (high-confidence)	*Podoviridae*		
TF25_BL_k141_2711	1	dsDNAphage	No	High-quality	96.22	High-quality draft genome	AAI-based (high-confidence)	*Microviridae*		
TF25_26wk_k141_3449	1	dsDNAphage	No	High-quality	96.88	High-quality draft genome	AAI-based (high-confidence)	*Crass_like*	*Firmicutes*	*Staphylococcus aureus*
TF25_12wk_k141_133747	1	dsDNAphage	No	Complete	100	High-quality draft genome	DTR (high-confidence)	*Microviridae*		
TF24_BL_k141_128850	0.993	dsDNAphage	No	Complete	100	High-quality draft genome	DTR (high-confidence)			
TF24_12wk_k141_112342	0.613	dsDNAphage	No	High-quality	100	High-quality draft genome	HMM-based (lower-bound)			
TF23_BL_k141_92706	1	dsDNAphage	No	Complete	100	High-quality draft genome	DTR (high-confidence)			
TF23_BL_k141_92707	0.993	dsDNAphage	No	Complete	100	High-quality draft genome	DTR (high-confidence)	*Microviridae*		
TF22_BL_k141_85607	1	dsDNAphage	No	High-quality	92.19	High-quality draft genome	AAI-based (medium-confidence)			
TF22_12wk_k141_147790	0.993	dsDNAphage	No	High-quality	92.75	High-quality draft genome	AAI-based (medium-confidence)			
TF20_BL_k141_200746	0.933	dsDNAphage	No	High-quality	91.81	High-quality draft genome	AAI-based (high-confidence)			
TF20_BL_k141_3999	1	dsDNAphage	No	Complete	100	High-quality draft genome	DTR (high-confidence)	*Microviridae*		
TF20_26wk_k141_233647	1	dsDNAphage	No	Complete	100	High-quality draft genome	DTR (high-confidence)			
TF19_BL_k141_202808	1	dsDNAphage	No	Complete	100	High-quality draft genome	DTR (high-confidence)			
TF19_BL_k141_127090	0.987	ssDNA	No	High-quality	100	High-quality draft genome	HMM-based (lower-bound)			
TF19_6WK_k141_141110	0.607	dsDNAphage	No	High-quality	100	High-quality draft genome	HMM-based (lower-bound)			
TF19_6WK_k141_170573	1	dsDNAphage	No	Complete	100	High-quality draft genome	DTR (high-confidence)			
TF18_BL_k141_42110	0.62	dsDNAphage	No	High-quality	94.52	High-quality draft genome	AAI-based (high-confidence)			
TF18_BL_k141_163513	0.993	dsDNAphage	No	High-quality	100	High-quality draft genome	AAI-based (medium-confidence)			
TF18_BL_k141_170467	1	dsDNAphage	No	High-quality	98.98	High-quality draft genome	AAI-based (high-confidence)	*Siphoviridae*		
TF18_BL_k141_168027	0.9	ssDNA	No	High-quality	100	High-quality draft genome	HMM-based (lower-bound)	*Circoviridae*		
TF18_12wk_k141_54481	0.927	dsDNAphage	No	High-quality	94.85	High-quality draft genome	AAI-based (medium-confidence)		*Firmicutes*	*Anaerobutyricum hallii*
TF14_BL_k141_45314	0.52	dsDNAphage	No	High-quality	100	High-quality draft genome	AAI-based (high-confidence)	*Circoviridae*		
TF14_6wk_k141_58937	0.513	dsDNAphage	No	High-quality	100	High-quality draft genome	HMM-based (lower-bound)			
TF14_6wk_k141_212672	1	dsDNAphage	No	Complete	100	High-quality draft genome	DTR (high-confidence)	*Microviridae*		
TF13_6wk_k141_94654	1	dsDNAphage	No	High-quality	91.46	High-quality draft genome	AAI-based (high-confidence)			
TF13_6wk_k141_130362	0.973	dsDNAphage	No	High-quality	100	High-quality draft genome	AAI-based (high-confidence)	*Microviridae*		
TF12_6wk_k141_51350	0.987	dsDNAphage	No	High-quality	100	High-quality draft genome	AAI-based (high-confidence)	*Microviridae*		
TF12_6wk_k141_19426	0.893	ssDNA	No	High-quality	100	High-quality draft genome	HMM-based (lower-bound)			
TF12_26wk_k141_1260	0.72	dsDNAphage	No	High-quality	99.71	High-quality draft genome	AAI-based (high-confidence)	*Circoviridae*		
TF11_BL_k141_25374	1	dsDNAphage	No	High-quality	99.19	High-quality draft genome	AAI-based (high-confidence)			
TF11_6wk_k141_189853	1	dsDNAphage	No	Complete	100	High-quality draft genome	DTR (high-confidence)			
TF10_BL_k141_6998	0.927	ssDNA	No	High-quality	100	High-quality draft genome	HMM-based (lower-bound)	*Circoviridae*		
TF10_6wk_k141_12732	0.96	dsDNAphage	No	Complete	100	High-quality draft genome	DTR (high-confidence)		*Firmicutes*	*Blautia obeum*
TF10_26wk_k141_34775	1	dsDNAphage	No	High-quality	100	High-quality draft genome	AAI-based (high-confidence)		*Firmicutes*	*Dorea formicigenerans*
TF10_12wk_k141_105830	1	dsDNAphage	No	Complete	100	High-quality draft genome	ITR (high-confidence)	*Podoviridae*	*Actinobacteria*	*Bifidobacterium pseudocatenulatum*
TF09_6WK_k141_44928	1	dsDNAphage	No	High-quality	98.5	High-quality draft genome	AAI-based (high-confidence)	*Microviridae*		
TF09_6WK_k141_33108	0.953	dsDNAphage	No	Complete	100	High-quality draft genome	DTR (high-confidence)			
TF09_6WK_k141_151028	1	dsDNAphage	No	Complete	100	High-quality draft genome	DTR (high-confidence)			
TF09_6WK_k141_179770	0.987	dsDNAphage	No	Complete	100	High-quality draft genome	DTR (high-confidence)			
TF09_6WK_k141_197621	0.993	dsDNAphage	No	Complete	100	High-quality draft genome	DTR (high-confidence)			
TF08_BL_k141_106168	1	dsDNAphage	No	High-quality	100	High-quality draft genome	AAI-based (medium-confidence)			
TF08_BL_k141_107198	0.993	dsDNAphage	No	High-quality	99.5	High-quality draft genome	AAI-based (high-confidence)	*Microviridae*		
TF08_6wk_k141_22027	0.993	dsDNAphage	No	High-quality	100	High-quality draft genome	AAI-based (high-confidence)			
TF07_26wk_k141_60620	0.887	dsDNAphage	No	High-quality	99.29	High-quality draft genome	AAI-based (medium-confidence)		*Firmicutes*	*Ruminococcus bromii*
TF07_26wk_k141_172533	0.993	dsDNAphage	No	Complete	100	High-quality draft genome	DTR (high-confidence)	*Microviridae*		
TF06_6WK_k141_138425	1	dsDNAphage	No	High-quality	93.01	High-quality draft genome	AAI-based (high-confidence)		*Firmicutes*	*Staphylococcus epidermidis*
TF06_12wk_k141_223411	0.993	dsDNAphage	No	Complete	100	High-quality draft genome	DTR (medium-confidence)			
TF06_12wk_k141_64301	1	ssDNA	No	High-quality	100	High-quality draft genome	HMM-based (lower-bound)	*Circoviridae*	*Firmicutes*	*Ruminococcus bromii*
TF05_BL_k141_23701	0.993	dsDNAphage	No	High-quality	91.88	High-quality draft genome	AAI-based (high-confidence)	*Podoviridae*		
TF05_12wk_k141_99670	0.993	dsDNAphage	No	Complete	100	High-quality draft genome	DTR (high-confidence)		*Firmicutes*	*Blautia obeum*

UViG	VirSorter2 score	Predicted genome type	Provirus	Completeness quality	Completeness (%)	Assembly quality	Completeness approach	Family	Host phylum	Host species
TF04_BL_k141_120155	0.987	dsDNAphage	No	High-quality	92.6	High-quality draft genome	AAI-based (high-confidence)			
TF04_BL_k141_49760	1	dsDNAphage	No	High-quality	93.47	High-quality draft genome	AAI-based (high-confidence)	*Microviridae*		
TF04_BL_k141_17732	1	dsDNAphage	No	Complete	100	High-quality draft genome	DTR (high-confidence)			
TF04_BL_k141_52388	0.993	dsDNAphage	No	Complete	100	High-quality draft genome	DTR (high-confidence)			
TF04_BL_k141_170090	0.987	dsDNAphage	No	Complete	100	High-quality draft genome	DTR (high-confidence)	*Microviridae*		
TF03_6wk_k141_69214	1	dsDNAphage	No	High-quality	93.21	High-quality draft genome	AAI-based (high-confidence)	*Microviridae*		
TF03_26wk_k141_44390	0.993	dsDNAphage	No	Complete	100	High-quality draft genome	DTR (high-confidence)			
TF03_26wk_k141_296978	1	dsDNAphage	No	Complete	100	High-quality draft genome	DTR (high-confidence)		*Actinobacteria*	*Bifidobacterium longum*
TF03_26wk_k141_165433	0.893	ssDNA	No	High-quality	100	High-quality draft genome	HMM-based (lower-bound)			
TF02_BL_k141_182005	0.987	dsDNAphage	No	High-quality	90.44	High-quality draft genome	AAI-based (high-confidence)			
TF02_6wk_k141_53938	1	dsDNAphage	No	High-quality	94.75	High-quality draft genome	AAI-based (high-confidence)			
TF01_BL_k141_26495	0.987	dsDNAphage	No	High-quality	94.77	High-quality draft genome	AAI-based (high-confidence)	*Siphoviridae*		
TF01_BL_k141_84458	0.987	dsDNAphage	No	Complete	100	High-quality draft genome	DTR (high-confidence)			
TF01_6wk_k141_186464	1	dsDNAphage	No	Complete	100	High-quality draft genome	DTR (high-confidence)	*Microviridae*		
TF01_12wk_k141_22742	1	dsDNAphage	No	High-quality	91.59	High-quality draft genome	AAI-based (high-confidence)	*Microviridae*		
DM08_D5_k141_143815	1	dsDNAphage	No	High-quality	100	High-quality draft genome	AAI-based (high-confidence)			
DM08_D2_k141_182366	0.987	dsDNAphage	No	Complete	100	High-quality draft genome	DTR (high-confidence)			
DM08_D1_k141_116917	1	dsDNAphage	No	High-quality	90.04	High-quality draft genome	AAI-based (high-confidence)			
DM07_D6_k141_181298	1	dsDNAphage	No	High-quality	94.87	High-quality draft genome	AAI-based (high-confidence)			
DM07_D3_k141_218728	0.993	dsDNAphage	No	Complete	100	High-quality draft genome	DTR (high-confidence)	*Microviridae*		
DM07_D2_k141_21788	1	dsDNAphage	No	High-quality	96.17	High-quality draft genome	AAI-based (high-confidence)	*Crass_like*		
DM07_D2_k141_190278	0.987	dsDNAphage	No	High-quality	100	High-quality draft genome	AAI-based (high-confidence)			
DM05_D1_k141_93639	0.747	ssDNA	No	High-quality	100	High-quality draft genome	HMM-based (lower-bound)	*Circoviridae*		
DM05_D1_k141_34991	0.92	ssDNA	No	High-quality	100	High-quality draft genome	HMM-based (lower-bound)			
DM03_D6_k141_71870	1	dsDNAphage	No	High-quality	99.36	High-quality draft genome	AAI-based (high-confidence)			
DM03_D6_k141_130918	1	dsDNAphage	No	Complete	100	High-quality draft genome	DTR (high-confidence)			
DM03_D1_k141_40827	0.813	dsDNAphage	No	High-quality	100	High-quality draft genome	HMM-based (lower-bound)	*Circoviridae*		
DF17_D9_k141_160210	1	dsDNAphage	No	Complete	100	High-quality draft genome	DTR (high-confidence)	*Microviridae*		
DF17_D9_k141_160260	1	dsDNAphage	No	Complete	100	High-quality draft genome	DTR (high-confidence)			
DF17_D8_k141_15289	0.687	dsDNAphage	No	Complete	100	High-quality draft genome	DTR (high-confidence)			
DF17_D7_k141_161441	0.987	dsDNAphage	No	Complete	100	High-quality draft genome	DTR (high-confidence)			
DF16_D7_k141_68837	0.587	dsDNAphage	No	High-quality	100	High-quality draft genome	AAI-based (high-confidence)	*Circoviridae*		
DF16_D6_k141_173953	0.86	dsDNAphage	No	Complete	100	High-quality draft genome	DTR (high-confidence)	*Circoviridae*		
DF14_D9_k141_30325	0.993	dsDNAphage	No	High-quality	90.04	High-quality draft genome	AAI-based (high-confidence)			
DF14_D8_k141_126097	0.893	ssDNA	No	High-quality	100	High-quality draft genome	HMM-based (lower-bound)			
DF14_D7_k141_156058	0.933	dsDNAphage	No	High-quality	97.9	High-quality draft genome	AAI-based (high-confidence)			
DF14_D7_k141_29281	0.98	dsDNAphage	No	Complete	100	High-quality draft genome	DTR (high-confidence)	*Siphoviridae*		
TM24_BL_k141_226221	0.827	dsDNAphage	No	Complete	100	High-quality draft genome	DTR (medium-confidence)			
TM16_6WK_k141_244989	0.84	dsDNAphage	No	Complete	100	High-quality draft genome	DTR (medium-confidence)			
TM15_6wk_k141_100939	0.853	ssDNA	No	High-quality	100	High-quality draft genome	AAI-based (medium-confidence)			
TM14_12wk_k141_111444	0.56	dsDNAphage	No	High-quality	100	High-quality draft genome	AAI-based (high-confidence)			
TM13_26wk_k141_215705	0.947	dsDNAphage	No	Complete	100	High-quality draft genome	DTR (high-confidence)			
TF43_12wk_k141_135518	0.527	dsDNAphage	No	High-quality	99.72	High-quality draft genome	AAI-based (medium-confidence)			
TF42_26wk_k141_222350	0.66	dsDNAphage	No	Complete	100	High-quality draft genome	DTR (high-confidence)			
TF17_26wk_k141_22992	0.873	ssDNA	No	High-quality	100	High-quality draft genome	AAI-based (medium-confidence)			
TF05_26wk_k141_64923	0.52	dsDNAphage	No	High-quality	100	High-quality draft genome	AAI-based (high-confidence)			
TM29_BL_k141_27404	0.66	dsDNAphage	No	Complete	100	High-quality draft genome	DTR (high-confidence)			
DF12_D4_k141_19442	0.94	dsDNAphage	Yes	High-quality	100	High-quality draft genome	AAI-based (high-confidence)			
DF16_D5_k141_31488	0.98	dsDNAphage	Yes	High-quality	100	High-quality draft genome	AAI-based (high-confidence)			
DM07_D2_k141_203513	0.913	ssDNA	Yes	High-quality	100	High-quality draft genome	HMM-based (lower-bound)			
DM08_D5_k141_107386	0.933	ssDNA	Yes	High-quality	100	High-quality draft genome	HMM-based (lower-bound)			
TF02_6wk_k141_180773	0.913	ssDNA	Yes	High-quality	100	High-quality draft genome	HMM-based (lower-bound)			
TF03_6wk_k141_178762	0.94	dsDNAphage	Yes	High-quality	100	High-quality draft genome	AAI-based (high-confidence)	*Microviridae*		
TF10_BL_k141_167042	1	dsDNAphage	Yes	High-quality	100	High-quality draft genome	AAI-based (high-confidence)			
TF14_BL_k141_145060	1	dsDNAphage	Yes	High-quality	100	High-quality draft genome	AAI-based (high-confidence)	*Myoviridae*		
TF16_12wk_k141_46692	0.993	dsDNAphage	Yes	High-quality	96.16	High-quality draft genome	AAI-based (high-confidence)			
TF19_BL_k141_3399	0.993	dsDNAphage	Yes	High-quality	100	High-quality draft genome	AAI-based (high-confidence)			
TF20_26wk_k141_213384	0.927	dsDNAphage	Yes	High-quality	100	High-quality draft genome	AAI-based (high-confidence)	*Microviridae*		
TF21_BL_k141_2517	0.64	dsDNAphage	Yes	High-quality	100	High-quality draft genome	HMM-based (lower-bound)	*Circoviridae*		
TF25_26wk_k141_49146	1	dsDNAphage	Yes	High-quality	100	High-quality draft genome	AAI-based (high-confidence)			
TF25_6WK_k141_132125	0.993	dsDNAphage	Yes	High-quality	100	High-quality draft genome	AAI-based (high-confidence)		*Proteobacteria*	*Escherichia coli*
TF29_6WK_k141_191250	0.993	dsDNAphage	Yes	High-quality	100	High-quality draft genome	AAI-based (high-confidence)			
TF29_BL_k141_53177	0.98	dsDNAphage	Yes	High-quality	100	High-quality draft genome	AAI-based (medium-confidence)			
TF30_BL_k141_117602	0.993	dsDNAphage	Yes	High-quality	100	High-quality draft genome	AAI-based (high-confidence)			
TF33_BL_k141_20531	0.92	dsDNAphage	Yes	High-quality	100	High-quality draft genome	AAI-based (high-confidence)	*Microviridae*		
TF34_26wk_k141_91691	0.993	dsDNAphage	Yes	Complete	100	High-quality draft genome	Provirus (medium-confidence)	*Myoviridae*	*Firmicutes*	*Anaerostipes hadrus*
TF36_12wk_k141_57272	0.987	dsDNAphage	Yes	High-quality	96.54	High-quality draft genome	AAI-based (high-confidence)	*Myoviridae*	*Firmicutes*	*Faecalibacterium prausnitzii*
TF37_BL_k141_91034	0.56	dsDNAphage	Yes	High-quality	100	High-quality draft genome	AAI-based (medium-confidence)			
TF41_6wk_k141_92433	0.82	dsDNAphage	Yes	High-quality	100	High-quality draft genome	AAI-based (high-confidence)	*Microviridae*		
TF44_BL_k141_13693	0.993	dsDNAphage	Yes	High-quality	91.78	High-quality draft genome	AAI-based (high-confidence)			
TF45_12wk_k141_97145	0.987	dsDNAphage	Yes	Complete	100	High-quality draft genome	Provirus (medium-confidence)		*Firmicutes*	*Hungatella hathewayi*
TF48_BL_k141_71125	0.98	dsDNAphage	Yes	High-quality	98.02	High-quality draft genome	AAI-based (high-confidence)			
TF50_6wk_k141_162815	0.973	dsDNAphage	Yes	High-quality	100	High-quality draft genome	AAI-based (high-confidence)		*Firmicutes*	*Ruminococcus bromii*
TF51_6wk_k141_110554	1	dsDNAphage	Yes	High-quality	98.75	High-quality draft genome	AAI-based (high-confidence)		*Firmicutes*	*Coprococcus catus*
TF51_BL_k141_18208	1	dsDNAphage	Yes	High-quality	96.66	High-quality draft genome	AAI-based (high-confidence)			
TM07_26wk_k141_179554	1	dsDNAphage	Yes	High-quality	93.94	High-quality draft genome	AAI-based (high-confidence)		*Bacteroidetes*	*Prevotella bivia*
TM07_BL_k141_202197	1	dsDNAphage	Yes	High-quality	93.98	High-quality draft genome	AAI-based (high-confidence)		*Bacteroidetes*	*Prevotella bivia*
TM10_26wk_k141_126582	0.993	dsDNAphage	Yes	High-quality	92.29	High-quality draft genome	AAI-based (high-confidence)		*Firmicutes*	*Roseburia intestinalis*
TM23_26wk_k141_8253	1	dsDNAphage	Yes	High-quality	95.07	High-quality draft genome	AAI-based (high-confidence)		*Firmicutes*	*Coprococcus catus*
TM27_26wk_k141_29921	1	dsDNAphage	Yes	High-quality	100	High-quality draft genome	AAI-based (high-confidence)			
TM35_BL_k141_169923	1	dsDNAphage	Yes	High-quality	100	High-quality draft genome	AAI-based (medium-confidence)		*Firmicutes*	*Anaerobutyricum hallii*
TM39_BL_k141_47331	0.947	dsDNAphage	Yes	High-quality	100	High-quality draft genome	HMM-based (lower-bound)			
